# TGS-GapCloser: A fast and accurate gap closer for large genomes with low coverage of error-prone long reads

**DOI:** 10.1093/gigascience/giaa094

**Published:** 2020-09-07

**Authors:** Mengyang Xu, Lidong Guo, Shengqiang Gu, Ou Wang, Rui Zhang, Brock A Peters, Guangyi Fan, Xin Liu, Xun Xu, Li Deng, Yongwei Zhang

**Affiliations:** BGI-Qingdao, BGI-Shenzhen, 2 Hengyunshan Road, West Coast New Area, Qingdao, 266426, China; State Key Laboratory of Agricultural Genomics, BGI-Shenzhen, Building 11, Beishan Industrial Zone, Yantian District, Shenzhen, 518083, China; BGI-Shenzhen, Building 11, Beishan Industrial Zone, Yantian District, Shenzhen, 518083, China; BGI-Qingdao, BGI-Shenzhen, 2 Hengyunshan Road, West Coast New Area, Qingdao, 266426, China; BGI Education Center, University of Chinese Academy of Sciences, Building 11, Beishan Industrial Zone, Yantian District, Shenzhen, 518083, China; BGI-Qingdao, BGI-Shenzhen, 2 Hengyunshan Road, West Coast New Area, Qingdao, 266426, China; BGI Education Center, University of Chinese Academy of Sciences, Building 11, Beishan Industrial Zone, Yantian District, Shenzhen, 518083, China; BGI-Shenzhen, Building 11, Beishan Industrial Zone, Yantian District, Shenzhen, 518083, China; MGI, BGI-Shenzhen, Building 11, Beishan Industrial Zone, Yantian District, Shenzhen, 518083, China; BGI-Qingdao, BGI-Shenzhen, 2 Hengyunshan Road, West Coast New Area, Qingdao, 266426, China; BGI-Shenzhen, Building 11, Beishan Industrial Zone, Yantian District, Shenzhen, 518083, China; Complete Genomics Inc., 2904 Orchard Pkwy, San Jose, CA 95134, USA; BGI-Qingdao, BGI-Shenzhen, 2 Hengyunshan Road, West Coast New Area, Qingdao, 266426, China; BGI-Shenzhen, Building 11, Beishan Industrial Zone, Yantian District, Shenzhen, 518083, China; BGI-Qingdao, BGI-Shenzhen, 2 Hengyunshan Road, West Coast New Area, Qingdao, 266426, China; State Key Laboratory of Agricultural Genomics, BGI-Shenzhen, Building 11, Beishan Industrial Zone, Yantian District, Shenzhen, 518083, China; BGI-Shenzhen, Building 11, Beishan Industrial Zone, Yantian District, Shenzhen, 518083, China; China National GeneBank, BGI-Shenzhen, Jinsha Road, Dapeng New District, Shenzhen, 518120, China; BGI-Shenzhen, Building 11, Beishan Industrial Zone, Yantian District, Shenzhen, 518083, China; China National GeneBank, BGI-Shenzhen, Jinsha Road, Dapeng New District, Shenzhen, 518120, China; BGI-Qingdao, BGI-Shenzhen, 2 Hengyunshan Road, West Coast New Area, Qingdao, 266426, China; State Key Laboratory of Agricultural Genomics, BGI-Shenzhen, Building 11, Beishan Industrial Zone, Yantian District, Shenzhen, 518083, China; BGI-Shenzhen, Building 11, Beishan Industrial Zone, Yantian District, Shenzhen, 518083, China; BGI-Shenzhen, Building 11, Beishan Industrial Zone, Yantian District, Shenzhen, 518083, China; Complete Genomics Inc., 2904 Orchard Pkwy, San Jose, CA 95134, USA

**Keywords:** gap closure, third-generation sequencing, genome assembly, ginkgo, MHC

## Abstract

**Background:**

Analyses that use genome assemblies are critically affected by the contiguity, completeness, and accuracy of those assemblies. In recent years single-molecule sequencing techniques generating long-read information have become available and enabled substantial improvement in contig length and genome completeness, especially for large genomes (>100 Mb), although bioinformatic tools for these applications are still limited.

**Findings:**

We developed a software tool to close sequence gaps in genome assemblies, TGS-GapCloser, that uses low-depth (∼10×) long single-molecule reads. The algorithm extracts reads that bridge gap regions between 2 contigs within a scaffold, error corrects only the candidate reads, and assigns the best sequence data to each gap. As a demonstration, we used TGS-GapCloser to improve the scaftig NG50 value of 3 human genome assemblies by 24-fold on average with only ∼10× coverage of Oxford Nanopore or Pacific Biosciences reads, covering with sequence data up to 94.8% gaps with 97.7% positive predictive value. These improved assemblies achieve 99.998% (Q46) single-base accuracy with final inserted sequences having 99.97% (Q35) accuracy, despite the high raw error rate of single-molecule reads, enabling high-quality downstream analyses, including up to a 31-fold increase in the scaftig NGA50 and up to 13.1% more complete BUSCO genes. Additionally, we show that even in ultra-large genome assemblies, such as the ginkgo (∼12 Gb), TGS-GapCloser can cover 71.6% of gaps with sequence data.

**Conclusions:**

TGS-GapCloser can close gaps in large genome assemblies using raw long reads quickly and cost-effectively. The final assemblies generated by TGS-GapCloser have improved contiguity and completeness while maintaining high accuracy. The software is available at https://github.com/BGI-Qingdao/TGS-GapCloser.

## Findings

### Introduction

The cost and time necessary to sequence 1 Mb of DNA has been decreasing at a speed beyond Moore's Law over the past decade [1]. Databases of genetic sequences have been growing dramatically, with the size of completed genomes increasing from small bacterial and fungal genomes to very large eukaryotic genomes. In addition to short-read next-generation sequencing technologies (NGS) that have enabled this dramatic increase in genome sequencing, recent state-of-the-art techniques, such as third-generation single-molecule long reads (TGS) [2, 3], synthetic long read (SLR) libraries [4–6], Hi-C [7], and BioNano physical maps [8], have provided long-range genome information to help increase the contiguity of genome assemblies. However, the finished assemblies for most large genomes (>100 Mb) remain imperfect and contain numerous gaps of unknown nucleic acids (represented by N's) [9, 10]. These gaps are often due to repetitive or difficult DNA sequences, polymorphisms between individual genomes of the same species, limitations of sequencing platforms, and algorithmic trade‐offs. The process of gap closure or gap filling can recover these unknown bases and extend scaftigs (contigs within a scaffold without N's) [11] to completely or partially bridge these gaps, and there is a need for tools to enable this on existing assemblies, especially for large highly complex eukaryotic genomes.

The first efforts to close gaps in genome assemblies were made using Fosmid and bacterial artificial chromosome libraries combined with Sanger sequencing [12]. But the cost and labor associated with this manual to semi-automated gap-closing process were very high [10] and practically limited to only very well-funded genome programs (e.g., the Human Genome Project). As NGS technologies lowered sequencing costs, new paired-end and mate-pair libraries made processes and several bioinformatics tools were designed to help improve the gap-closing process [13–17]. These tools were based on *k*-mer extension or local reassembly algorithms but were hindered by large CPU and memory consumption. In addition, these strategies rarely spanned repetitive DNA regions and tended to cause more misassemblies due to the short read lengths of NGS.

Current single-molecule TGS technologies, such as those of Pacific Biosciences (PacBio) and Oxford Nanopore Technologies (ONT), have the potential to break through these limitations because their reads can exceed 100 kb and are typically longer than most DNA repeats [18]. Although the *de novo* genome assembly using TGS reads alone is possible, the lower raw read accuracy relative to NGS platforms generally requires sufficient sequencing coverage and high computational costs for error correction of the assembly [19]. This correction is necessary because these base-calling errors may cause frameshifts and other changes in the gene-coding or regulatory regions and thus cause inaccurate interpretation of the genome [20].

Recently, there have been several hybrid assemblers designed to take advantage of the combination of both TGS and NGS read data. Most construct a final assembly graph by mixing NGS contigs and TGS long reads based on the Overlap-Layout-Consensus or string graph algorithm [21], or connect the contigs generated by NGS with their alignments against long reads [22–24]. In contrast, the gap-closing algorithms provide a direct way to reduce the computing complexity and costs through improvements only in the missing regions and preservation of the majority of the existing assembly information. PBJelly [10] is the first tool to use PacBio reads to close gaps through local assembly of the long reads in gap regions. FGAP [25] selects the best matched pre-assembled contig to fill gaps based on BLAST [26] alignments. GMcloser [27] tries to increase the accuracy of gap closure using likelihood-based classifiers. Cobbler [28] uses new aligners to accelerate the build-up of the relationship between long high-quality sequences (usually scaftigs/contigs from other assemblies) and input scaffolds, and patches the gaps if the alignment of long sequence to the assembly meets a threshold score. Finally, LR_Gapcloser [29] reduces the computational costs of alignments by fragmenting long reads into tags and aligning the short tags against scaffolds instead of the whole long reads. These tools have been widely used to close gaps with TGS long reads, but their efficiencies and accuracies are very much dependent on the quality of the long reads used. PBJelly improves the quality of inserted long reads through local assembly but requires sufficient coverage. Other tools bypass the limitation of input quality and require or recommend pre–error-corrected long reads or pre-assembled contigs. However, the additional assembly or correction for all reads prior to gap closure necessitates adequate coverage of expensive long reads or additional short NGS reads. This requires extra time and memory consumption, especially for large genomes. In addition, the correction algorithms might trim ambiguous segments [30] and split long reads into short fragments [31] due to the undetermined bases, thus losing valuable length information.

Three key factors should be considered to develop a TGS gap-closing algorithm. First, use TGS data as little as possible. Although the cost has been decreasing [32], the gap-closing efficiency is still the first priority, particularly for small laboratories or small projects. As such, local reassembly or pre–error correction based on the long-read overlaps is not preferable. Another important factor is the accuracy and precision in the selection of long reads to fill the gaps. It has been demonstrated that the number of assembly errors caused by gap-closing tools is higher than that of *de novo* assembled scaftigs [27]. The misalignments of long reads against the scaffolds caused by base-calling errors or repeats may increase the probability of large misassembly events. An effective scoring mechanism can prevent the gap-closing tools from making some of these incorrect selections of reads. Finally, the filled sequences should not diminish the single-base level accuracy of the whole assembly and thus affect the quality of downstream analyses. There is still a need for error correction for the inserted raw long-read segments. It should be noted that recently PacBio improved its base-calling accuracy to 99.8% [33], which may simplify the problem; however, this improved accuracy comes at a significant cost to throughput and read length.

In this work, we describe a software tool, TGS-GapCloser, that uses low-coverage error-prone long reads to close gaps in large genomes more efficiently and accurately than other current gap-closing tools. Using only 10× coverage of ONT or PacBio long reads [34, 35] applied to 3 *de novo* assembled human genomes we demonstrate an increase in the scaftig NG50 by 11.0- to 45.0-fold and an increase in the scaftig NGA50 by 6.8- to 30.6-fold. Furthermore, we show that 71.6% of gaps in the ultra-large genome assembly of ginkgo can be closed using just 10.5× coverage of corrected PacBio reads, increasing the scaftig N50 from 57.1 to 364.8 kb. A hybrid strategy of updating a draft *de novo* genome assembly with TGS-GapCloser is an efficient and accurate strategy for improving the quality of gene annotation and structure variation detection. Ultimately this will help lead to high-quality downstream analyses of ontogeny, phylogeny, and evolution.

### Data description

Three datasets from 2 species containing large genomes were used to examine the gap-closing results by TGS-GapCloser: human, human Chr19, and ginkgo. We sequenced *Homo sapiens* (HG001/NA12878, Coriell Cat# GM12878, RRID:CVCL_7526) using the MGIEasy stLFR Library Prep Kit on the DNBSEQ-G50 platform (formerly known as BGISEQ-500) (DNBSEQ-G50, RRID:SCR_017979), generating a total 660 Gb of read data. Reads mapped to the Chr19 reference were also extracted for comparisons and further analysis. These short reads were assembled using MaSuRCA [23] (MaSuRCA, RRID:SCR_010691) version 3.3.1 or Mercedes (in-house tool) to obtain short but highly accurate contigs, and the SLR long-range (co-barcode/read cloud) and short-range (paired-end) information provided by the single-tube long fragment reads (stLFR) technique were exploited to do further scaffolding by SLR-superscaffolder [36] (version 1.0.0). In addition, Supernova [37] (version 2.1.1) (Supernova assembler, RRID:SCR_016756) was used to obtain draft scaffolds despite being originally designed to assemble 10X Genomics data. To test the potential application of TGS-GapCloser, we used newly generated data from both long-read platforms (ONT and PacBio) to close gaps in human genome assemblies: ONT MinION Rel3 dataset (Rel3) [34] and PacBio CCS HiFi dataset (HiFi) [35].

The genome assembly of a female *Ginkgo biloba* (estimated genome size ∼12 Gb) used in this study was obtained from Guan et al. [38] and was initially assembled with SOAPdenovo2 (SOAPdenovo2, RRID:SCR_014986) [13] and updated using Hi-C data [38]. The PacBio reads for ginkgo were sequenced on a PacBio Sequel using a Sequel Sequencing Kit 3.0 Bundle (4 rxn). A total of 256 Gb of read data with an average read length of 38,623 bp was generated. Error correction by Canu (Canu, RRID:SCR_015880) [30] reduced the data size to 126 Gb, with an average read length of 10,722 bp. Statistics for input assemblies and sequencing reads can be found in Supplementary Tables S1 and S4, respectively.

### Algorithm and implementation of TGS-GapCloser

**Figure 1:**
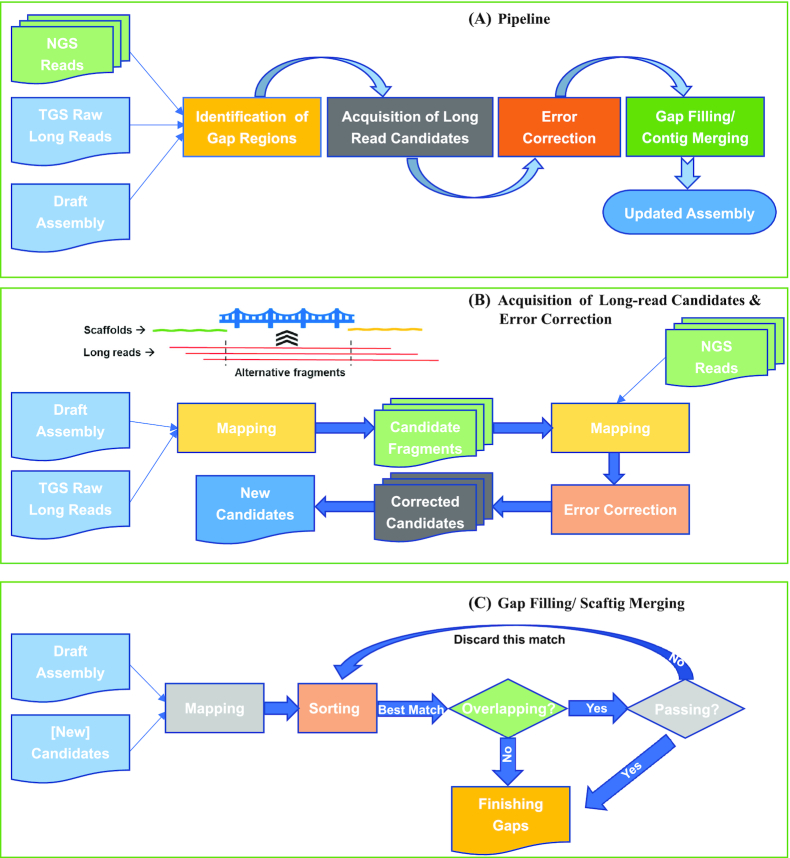
A schematic of TGS-GapCloser workflow. (A) A flow chart of the overall algorithm; (B) a schematic description on how gap regions are identified, the acquisition of candidate long-read fragments, and the error correction of alternative sub-long reads; (C) a detailed flow chart for gap filling or scaftig merging in a gap region with the most appropriate medium/long-range information provided by long reads.

TGS-GapCloser can accept as input any type of TGS long reads or other pre-assembled contigs to fill gaps in a draft assembly in the 4 steps as shown in Fig. 1: (i) identification of gap regions in the draft assembly; (ii) acquisition of candidates from the alignments of long reads against gaps; (iii) base-level error correction of alternative sub-long reads; and (iv) gap closure using the error-corrected candidates with the highest score for each gap or linkage of the neighboring scaftigs with overlaps.

The input scaffolds were first split into fragments called scaftigs from the observed N positions in the scaffolds, and each pair of neighboring scaftigs based upon their positions in the shared scaffold were defined as a gap to be filled. TGS-GapCloser retains the input scaffold information as the base-level accuracy and the order and orientation of scaftigs, but not the estimated gap size. This is caused by the lack of sufficient resolution in the long-range information provided by SLR, Hi-C, or BioNano to accurately predict the size of gaps below ∼10 kb.

We used minimap2 (Minimap2, RRID:SCR_018550) [39] to align long reads against each gap to obtain the corresponding candidate fragments. A candidate for a specific gap is defined as the segment truncated from the aligned long reads in the N region between 2 neighboring scaftigs plus 2-kb-long of aligned sequence on both sides of the gap. Each long read might provide several candidate sequences depending on the length spanned and base-calling accuracy but is limited to give at most 1 candidate for the same gap. This is to avoid redundant alignments induced by the alignment algorithm and the high error rate of TGS reads.

The quantity and quality of candidate reads determine the efficiency and accuracy of gap closure. Thus, we designed a scoring system of candidates for quality control and filtration based on the length and identity ratio (matched bases/aligned bases) of the alignment between a long-read candidate and flanking scaftig sequence next to the gap. The score QS is given by

$$\begin{equation*}
\mathrm{QS} = a \cdot \mathrm{log}{A_i} + b \cdot \mathrm{log}{I_i} + a \cdot \mathrm{log}{A_{i + 1}} + b \cdot \mathrm{log}{I_{i + 1}},
\end{equation*}$$

where *A* refers to the alignment length and *I* refers to the identity ratio for the *i*th and *i+1*th scaftigs, respectively; *a* and *b* are 2 arbitrary coefficients to distinguish *A* and *I*’s weights on the score and have been tuned to 1:6 for the ONT dataset as default. For each gap, a maximum of 10 candidates with the highest QS were chosen for error correction in order to limit the size of data for further analysis. To further reduce the complexity and requirements on computational resources, the overlapped candidates in the same long read were clipped and merged prior to the correction. Either Pilon (Pilon, RRID:SCR_014731) [31] or Racon (Racon, RRID:SCR_017642) [40] was used to enhance the base-level accuracy of merged sequences. Pilon is capable of correcting individual base errors, small indels, and local misassemblies with short but accurate NGS reads, while Racon corrects sequencing errors by constructing a SIMD-accelerated partial-order alignment graph from the overlap of long reads. The short reads were aligned to candidates by minimap2 with the option -k14 -w5 -n2 -m20 -s40 –sr –frag yes.

The corrected candidates were realigned to the gap and scored again, and finally the one with the highest QS was selected to fill the gap. The correction not only increased the single-base accuracy but also helped to find the best final candidate. We hypothesized that the QS of a candidate with higher-quality alignments would be increased due to the more precise mapping to the gap region after error correction, while the candidates with relatively lower-quality alignments tend to fail to be mapped. After final alignment to the gap region, those 2-kb sequences aligning to the scaftigs on either side of the gap were removed and only the bases filling the gap from the highest-scoring candidate were retained.

If the highest-scoring candidate resulted in a reduction in bases within the gap, then the gap would collapse to a single scaftig according to the alignment. A portion of scaftigs could have overlaps with other scaftigs because of incorrect paths during the initial assembly graph or over-aggressive scaftig extension. However, a TGS read spanning the gap has the ability to solve the overlap if 2 scaftigs can be mapped to the correct positions. Candidates resulting in a reduction in bases were selected only with more stringent criteria because large indels or homopolymeric repeats in long reads tend to cause incorrect overlaps. Gaps lacking any candidates could not be closed; in some cases this could be due to misassemblies in the draft assembly.

TGS-GapCloser is coded in the C++ programing language (requires GCC 4.4+). It uses minimap2 to obtain alignments, and Pilon (requires Java runtime 1.7+) or Racon (requires GCC 4.8+) to correct candidate fragments. The algorithm automatically identifies gaps and tries to find the best matched long-read fragments to close gaps or merge adjacent scaftigs. To accelerate the gap closure without losing efficiency and accuracy, TGS-GapCloser only selects a limited number of fragmented long reads as candidates for subsequent error correction and competition. This also reduces the computational complexity and improves the accuracy through a straightforward but efficient scoring system (Supplementary Table S3) and correction-enhanced mapping (Supplementary Table S4). In addition, the aligner, minimap2, shows noticeable improvements in speed and mapping accuracy for error-prone long reads [39], helping to shorten the time of sequence alignment and improve the overall quality of the final gap-closed sequence. The details of each step of this process, including gap identification, mapping, candidate identification, error correction, and final candidate selection, are recorded. The final output is reported in FASTA format, with a log file describing the detailed insertion/merging information.

### Gap closure in the human genome

Three assemblies and 2 TGS datasets were used to benchmark the utility of TGS-GapCloser in gap closure and scaftig merging in the human genome. Using the same co-barcoded short-read stLFR library, the whole genome was assembled by (i) MaSuRCA-assembled contigs + scaffolds from SLR-superscaffolder, (ii) Mercedes-assembled contigs + scaffolds from SLR-superscaffolder, and (iii) contigs and scaffolds assembled by Supernova using all of the barcoded long-range information. Although MaSuRCA itself can scaffold the contigs, the assembler does not utilize the SLR information and generates relatively short scaffolds. As such, it is necessary to use SLR-superscaffolder to obtain a scaffold NG50 comparable to Supernova.

**Figure 2:**
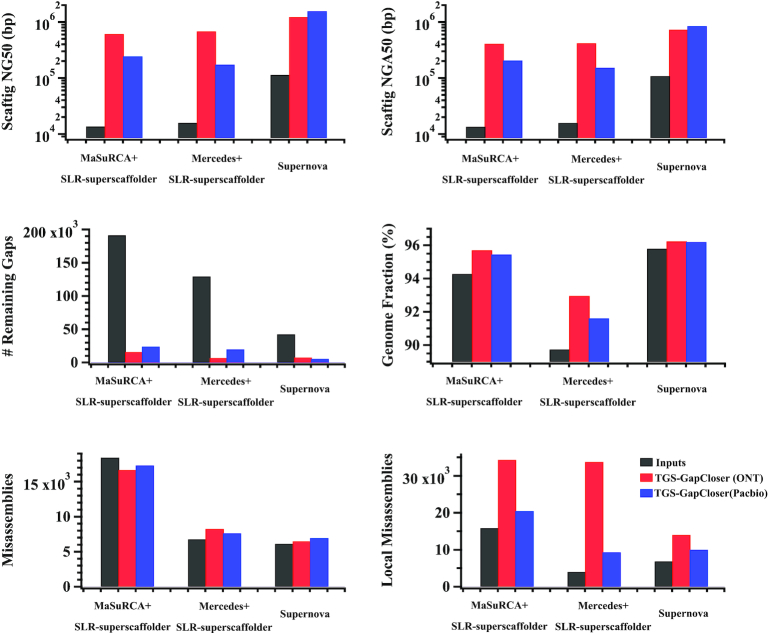
Gap-filling improvements and effects on the draft assemblies produced by TGS-GapCloser. (A) scaftig NG50, (B) scaftig NGA50, (C) number of remaining gaps, (D) genome fraction, (E) misassemblies, and (F) local misassemblies for the human genome were calculated by direct counting or reported by QUAST.

To assess the efficiency of TGS-GapCloser, we used ∼10× coverage of long reads from an ONT Rel3 dataset with a claimed mean read identity of 82.73% [34] and a PacBio HiFi dataset with the claimed mean read concordance of 99.8% [33]. The long-read fragments from ONT Rel3 were corrected by Pilon with NGS short reads while those from HiFi were corrected by Racon using the long reads themselves. Fig. 2 describes the improvements in the assembly evaluation given by QUAST (QUAST, RRID:SCR_001228) [41] after gap closure. Up to 91.8% of a total of 191,189; 94.8% of a total of 129,408; and 86.8% of a total of 42,359 gaps were successfully closed by TGS-GapCloser for 3 assemblies. The scaftig NG50 increased from 13.6 to 610.6 kb with the ONT Rel3 reads and to 243.7 kb with the PacBio HiFi reads for Assembly 1, 15.8 to 682.4 kb with the ONT Rel3 reads and to 173.7 kb with the PacBio HiFi reads for Assembly 2, and 113.0 to 1,229.2 kb with the ONT Rel3 reads and to 1,566.1 kb with the PacBio HiFi reads for Assembly 3. Additionally, the corresponding scaftig NGA50 was also improved from 13.4 to 411.1  and 205.9 kb for Assembly 1, 15.7 kb to 418.2 and 153.2 kb for Assembly 2, and 108.5 kb to 734.2 and 849.7 kb for Assembly 3 with ONT Rel3 and PacBio HiFi reads, respectively. Note that our current algorithm does not split or merge input scaffolds. However, the scaffold NG50 and NGA50 may change as a result of the replacement of N's and the combining of scaftigs. As listed in Supplementary Table S2, the genome fraction against the reference also increased by 1.4%, 3.2%, and 0.4% with ONT Rel3 reads and 1.2%, 1.9%, and 0.4% with PacBio HiFi reads for Assemblies 1–3, respectively, indicating that many of the sequence filled gaps in each assembly are mapped to the human reference assembly. The application of the ONT Rel3 read dataset increased the large-scale misassemblies (>1 kb) created by the filled sequences by 22.2% and 6.3% in Assemblies 2 and 3 but decreased misassemblies by 9.5% in Assembly 1 as a result of the updated scaffolds mapping more precisely to the reference. In addition, local misassemblies (<1 kb) increased by 1.2-, 7.4-, and 1.1-fold for Assemblies 1–3, respectively, despite the ONT Rel3 reads having undergone error correction. The PacBio HiFi dataset, with higher initial read accuracy, resulted in fewer induced misassemblies and local misassemblies: −6.1% and 0.3-fold for Assembly 1, 13.1% and 1.3-fold for Assembly 2, and 13.9% and 0.5-fold for Assembly 3. Overall, ONT Rel3 reads closed more gaps, resulting in better contiguity than PacBio HiFi reads, with the trade-off of inducing more assembly errors. This is because the ONT Rel3 dataset is composed of single long reads (the longest >500 kb) while the PacBio HiFi dataset produces ∼10-fold coverage of each single read followed by a read consensus process resulting in ∼13-kb final reads with higher single-base accuracy (Fig. S3). The performance of TGS-GapCloser is substantially dependent on both the length and the accuracy of input long reads, which are current balancing factors for single-molecule sequencing techniques.

After gap closure of the assemblies, BUSCO [42] (version 3.0.2) (BUSCO, RRID:SCR_015008) analysis indicated that there are possible improvements for bioinformatics analysis such as gene annotation. The assemblies were compared against the vertebrata_odb9 database. It revealed that 90.5%, 89.7%, and 94.1% of the expected vertebrate genes are complete for Assemblies 1–3, respectively, with ONT Rel3, and 90.4%, 85.3%, and 94.0% for Assemblies 1–3, respectively, with PacBio HiFi. A substantial improvement was observed from the original 86.2%, 76.6%, and 90.7% for Assemblies 1–3, respectively.

### Gap closure in the ultra-large genome of ginkgo

*G. biloba* is considered a living fossil, with its form and structure essentially unchanged for >270 million years. This makes it unique in the evolutionary tree of life [43]. We applied TGS-GapCloser to the chromosomal-level assembly of *G. biloba* [38] using ∼10.5× coverage of Canu-corrected PacBio reads. The input assembly has been assigned to 13 chromosomes totaling 9,570,195,624 bp of sequence interrupted by 613,821 gaps. TGS-GapCloser filled 71.6% of the gaps in the assembly and replaced N-containing regions by 411,608,879 bp of sequence. This resulted in the scaftig N50 increasing from 57.1 to 364.8 kb. Previously most gap-closing tools had only been used for bacterial and fungal genomes or small eukaryotic genomes [25, 27, 44]. This is the first example of using a gap-closing tool on an ultra-large genome with reasonable computational resources.

### Validation of gap-closing sequences

As a sanity check, we mapped the gaps in input scaffolds to the human reference assembly, generated filled sequences based on the reference assembly, and compared these to the filled long-read fragments created by TGS-GapCloser. Note that the statistics for the filled gaps described here are different from those given by direct counting (Fig. 2C) because the gaps closed with scaftig overlapping are not counted. The evaluation (Table 1) consists of 2 parts: long-read accuracy and single-base–level accuracy. For the selection of fragments inserted by TGS-GapCloser, the validated PPV ranges from 98.1% to 62.0% and the sensitivity from 96.4% to 51.2% for the 3 assemblies. Overall, gap-closing results with PacBio HiFi reads show relatively higher PPV due to its higher read accuracy but lower sensitivity due to its shorter read length. The accuracy of Assemblies 1 and 2 is better than that of Assembly 3, which has more small gaps. This result implies that TGS-GapCloser tends to fill large gaps.

**Table 1:** Gap-closing accuracy statistics and computational consumption for TGS-GapCloser

Input data	Accuracy in long-read selection						Accuracy in single-base level					
	No. of closed gaps	No. of closed gaps in theory	PPV (%)	Sensitivity (%)	Runtime (hours)	Peak memory usage (GB)	No. of filled bases (bp)	No. of filled bases in theory (bp)	QV of input raw long reads (Phred)	QV of input scaftigs (Phred)	QV of filled long reads with correction (Phred)	QV of output scaftigs (Phred)
MaSuRCA + SLR-superscaffolder + TGS-GapCloser (ONT)	75,629	74,353	96.6	96.3	259	50	335,541,557	353,352,038	7.63	40.51	23.24	36.06
MaSuRCA + SLR-superscaffolder + TGS-GapCloser (PacBio)	74,321	74,353	98.2	89.8	13	33	198,327,815	353,352,038	26.99	40.51	35.52	37.64
Mercedes + SLR-superscaffolder + TGS-GapCloser (ONT)	58,938	61,267	97.7	93.4	145	51	352,316,717	497,208,670	7.63	48.09	23.23	40.19
Mercedes + SLR-superscaffolder + TGS-GapCloser (PacBio)	52,116	61,267	98.4	75.6	11	32	146,148,151	497,208,670	26.99	48.09	36.25	42.29
Supernova + TGS-GapCloser (ONT)	22,563	24,760	62.0	51.2	163	74	49,669,581	38,276,270	7.63	48.72	23.15	46.11
Supernova + TGS-GapCloser (PacBio)	26,919	24,760	76.1	61.2	20	38	22,178,115	38,276,270	26.99	48.72	34.82	46.48

All datasets were run with 42 threads. Note that the peak memory consumption by Pilon or Racon is not counted. The higher speed of runs using the PacBio HiFi dataset mainly originates from the use of Racon to correct fragments with long reads. Note that QUAST accepts <10 continuous N's in the scaftig. PPV: positive predictive value; QV: quality value.

In terms of single-base–level accuracy, we calculated the Phred-like concordance quality value (QV) by the method described by Wenger et al. [33]. The QV of the inserted long-read fragments was improved after error correction. However, the overall QV of the assembly decreased: the scaftig QV was reduced from 45.8 to 40.8 with ONT Rel3 reads and to 42.1 with PacBio HiFi reads on average. Accuracy decline was less obvious with PacBio HiFi reads after error correction, which was consistent with the higher PPV in the long-read selection. That said, the final assemblies had >Q40 single-base quality, making them comparable to or even better than most *de novo* TGS assemblies with pre–error correction and polishing [33, 34].

### Performance of TGS-GapCloser for large genomes

TGS-GapCloser is relatively fast and accurate. For the human genome, it consumed as little as 155 CPU hours in total and 32 GB of peak memory. The algorithm design substantially reduced the time for read mapping and error correction. Gap closure using the NGS-based error correction for the inserted sequences (∼189 hours on average) was much slower than that with the TGS-based correction (∼15 hours on average). As a comparison, the *de novo* assembly for 30× coverage of long reads requires ∼40,000 CPU hours for ONT and ∼62,000 CPU hours for PacBio [34]. The computation can be further reduced by not using error correction. It only took 541 CPU hours for the ginkgo genome using pre-corrected PacBio reads. TGS-GapCloser requires low coverage of expensive long reads without pre–error correction, making this approach more cost-effective and suitable for research projects with limited budgets.

### Comparison with other gap-closing tools

We did not compare TGS-GapCloser to NGS gap-closing tools because the use of TGS read information can span the repetitive or other complicated regions in the assembly that *k*-mer–based extension approaches cannot. In this article, we used a variety of published long-read gap closers, including PBJelly (PBJelly, RRID:SCR_012091) [10], FGAP [25], GMcloser (GMcloser, RRID:SCR_000646) [27], Cobbler [28], and LR_Gapcloser [29], on the same Chr19 Mercedes + SLR-superscaffolder assembly with ONT Rel3 reads, and systematically compared their performances.

The comparison shows that TGS-GapCloser performed best overall among 6 tools with this combination of inputs (Table 2). Its gap-closing efficiency was considerably higher than that of other tools, reducing the number of gaps from 2,600 to 288, and increasing the scaftig NG50 from 9.6 to 194.5 kb. LR_Gapcloser, the next best performing tool for total gaps filled, was able to increase the scaftig NG50 to 157.2 kb. FGAP closed a similar number of gaps to LR_Gapcloser, leaving 458 gaps unfilled, and was able to increase the scaftig NG50 to 127.4 kb. The remaining tools, PBJelly, GMcloser, and Cobbler, left >1,000 gaps unresolved and did not show much increase in scaftig lengths.

**Table 2:** Gap-filling statistics for TGS-GapCloser and other gap-closing tools

Input data	Unfilled gaps	Misassembly	Local misassembly	Scaffold NG50 (bp)	Scaffold NGA50 (bp)	Scaftig NG50 (bp)	Scaftig NGA50 (bp)	Runtime (min)	Peak memory (GB)
Draft assemblies	2,600	176	126	1,561,142	196,307	9,687	9,464	–	–
TGS-GapCloser	288	187	324	1,426,438	383,995	194,512	149,166	12	16.37
PBJelly	1,730	664	741	1,240,439	83,803	29,715	19,247	3,137	9.93
FGAP	458	867	684	1,871,611	44,244	127,982	28,615	2,687	35.06
GMcloser	2,600	175	125	1,561,142	195,886	9,570	9,335	17,140	11.39
Cobbler	1,475	230	516	1,522,592	176,960	24,072	18,217	24	9.43
LR_Gapcloser	447	1,064	1,076	1,561,028	27,211	157,181	18,216	74	2.90

All datasets were run with 16 threads on the same computer. Note that QUAST accepts <10 continuous N's in the scaftig.

In terms of accuracy, TGS-GapCloser led to the largest increase (>5.2×) in the scaftig NGA50 (16.0 folds to input) with fewer misassemblies. Although FGAP and LR_Gapcloser extended the scaftig NG50 longer than 100 kb, both generated more misassemblies, resulting in a shorter scaftig NGA50. Most gap-closing tools were originally designed for error-corrected long reads or high-quality pre-assembled contigs, and as a result, their performances are mostly unsatisfactory with low-coverage raw ONT reads.

In addition, we analyzed the running time and memory consumption for each tool under the same operating conditions. TGS-GapCloser ran approximately 261-, 224-, 1,428-, 2-, and 6-fold faster than PBJelly, FGAP, GMcloser, Cobbler, and LR_Gapcloser, respectively. GMcloser and FGAP (based on BLAST [26]) and PBJelly (based on BLASR [45]) were the most time-consuming. The relatively higher memory requirement of TGS-GapCloser was due to the error correction needs. LR_Gapcloser used short-tag comparisons to avoid long-read alignments and thus required less memory than others.

### Effects of long-read coverage

It is worth noting the effects of long-read coverage on the gap closure. We randomly extracted 1×, 5×, 10×, 20×, and 29× coverages of mapped ONT Rel3 reads against the Chr19 reference and individually applied them to the same Chr19 MaSuRCA + SLR-superscaffolder assembly by TGS-GapCloser using the same default parameters. As shown in Supplementary Fig. S1A, the number of closed gaps and the total filled bases grew with the increasing coverage but saturated at ∼10× coverage, close to the level of theoretically filled gap numbers and bases. Surprisingly, the total time usage did not change much with the increasing coverage, but the peak memory showed an approximately linear growth (Supplementary Fig. S1B). With more long reads, the sensitivity of inserted sequences increased from 22.1% to 87.4% while the PPV remained similar (Supplementary Fig. S1C). In terms of single-base–level accuracy, the average concordance QV of inserted sequences decreased as more gaps were closed, but there was a negligible effect on that of scaftigs (Supplementary Fig. S1D). The result indicates that TGS-GapCloser closes a considerable number of gaps with high-quality sequence while using low-coverage error-prone long reads. In contrast, a high-quality long-read assembly requires ≥30× sequencing coverage [19].

### Improvements in the MHC region

TGS read data have been shown to be useful in the assembly of the human MHC region. This ∼6-Mb region in Chr6 is difficult to assemble with short reads only owing to high repetition and polymorphism [34]. It contains class I and II human leukocyte antigen genes, important to cancer and immunity studies [46]. We analyzed 3 assemblies before and after gap closure to investigate the contiguity and accuracy in this region as presented in Table 3. For Assembly 3, a portion of a single long scaffold (>29 Mb) completely covered the MHC region, while several portions of 2 or 3 scaffolds (0.6–27 Mb) covered the region for Assemblies 1 and 2. Gap closure with the ONT Rel3 dataset reduced the number of scaftigs in those scaffolds from 339, 271, and 76 to 31, 26, and 12 in Assemblies 1, 2, and 3, respectively. In addition, TGS-GapCloser reduced the percentage of N bases from 15.2% of the total assembly down to 3.7% on average while increasing the genome fraction mapped to the reference assembly from 81.52% to 91.17%. As a result, the scaftig NG50 and NGA50 improved from 46.7 to 585.1 kb and 41.0 to 300.4 kb. Importantly, this result would be expected to improve the gene annotations, structural variation detection, and single-nucleotide polymorphism calling in this region. Although TGS long reads resolved the MHC locus into 1 or several contigs, the relatively short contig NGA50 (52.6 kb), low genome fraction (59.86%), and numerous local misassemblies indicated that improving the accuracy in short-range information was still a challenge for TGS applications.

**Table 3:** Improved assemblies in the MHC region by TGS-GapCloser

Statistics	MaSuRCA + SLR-superscaffolder + TGS-GapCloser		Mercedes + SLR-superscaffolder + TGS-GapCloser		Supernova + TGS-GapCloser		Reference [33]	
	Draft	Updated	Draft	Updated	Draft	Updated	Rel3	Rel5
No. of scaffolds (>1 kb)	2	2	3	3	1	1	–	–
No. of scaftigs/contigs (>1 kb)	339	31	271	26	76	12	7	1
Non-N bases (bp)	5,293,785	5,907,069	4,134,156	5,445,373	5,831,980	5,988,090	5,739,339	5,628,041
No. of gaps	343	31	268	23	81	16	–	–
Scaffold NG50 (bp)	3,400,000	3,400,000	4,400,000	4,400,000	6,000,000	6,000,000	–	–
Scaffold NGA50 (bp)	232,462	396,537	182,662	429,613	649,591	534,616	–	–
Scaftig/contig NG50 (bp)	17,483	324,807	12,244	450,213	110,320	980,326	3,007,673	5,628,041
Scaftig/contig NGA50 (bp)	16,630	199,405	11,901	321,624	94,556	380,102	49,485	52,555
Genome fraction (%)	82.801	92.623	67.869	85.609	93.887	95.292	62.521	59.855
No. of misassemblies	11	25	13	22	15	17	20	53
No. of local misassemblies	34	101	11	122	29	42	546	484

The statistical results were generated by QUAST. Note that QUAST accepts <10 continuous N's in the scaftig/contig.

### Future direction

There are potential future improvements to consider for TGS-GapCloser. The selection of inserted sequences largely depends on the performance of the aligner. Although minimap2 performs well in most cases, the alignment results in errors if the pairwise sequences share small overlaps. We believe that this can be solved by using other aligners or additional parameter optimization. In addition, the computational consumption by error correctors or polishers is still significant, even with our efforts to reduce the input data size as much as possible. As error correction tools are updated and ideally become more efficient, TGS-GapCloser performance will benefit from these improvements. In addition, as long-read error rates continue to decrease, as promised by ONT and PacBio, it may be possible to eliminate this extra step of error correction. Finally, we use the input scaffolds including the orientation and order relations of scaftigs to retain the existing assembly information, ignoring possible assembly errors. As a future update we plan to use the information provided by TGS reads to correct scaftig errors within the same scaffold and link different scaffolds if sufficient overlapping is present. Nonetheless, in its current form, TGS-Gapcloser enables the combination of different genetic information with different lengths and resolutions and makes it possible to complete high-quality (ultra) large genome assemblies.

## Methods

### Gap closing with other tools

We compared the performance of TGS-GapCloser with that of 5 TGS gap-closing tools, including PBJelly (version PBSuite_15.8.24) (PBJelly, RRID:SCR_012091), FGAP (version 1.8.1), GMcloser (version 1.6.2) (GMcloser, RRID:SCR_000646), Cobbler (version 0.6.1), and LR_Gapcloser (no version information available) (LR_Gapcloser, RRID:SCR_016194). Some were unable to close gaps using the default parameters on low-coverage raw TGS reads. As a result, we needed to tune FGAP to be able to close large gaps (<100 kb,  default <500 kb). For GMcloser, we used the example parameters for long reads from the manual. In addition, the parameters for Cobbler were tuned according to the authors’ guidance on GitHub. All other tools were run using the default parameters for ONT data.

### Validation of gap-closing results

We evaluated the gap-closing accuracy at 2 levels: the selection of long reads and the single-base level. The former is determined by whether the algorithm can capture the best long read to close the corresponding gap. This will affect the detection of chromosomal variations, large relocations, and inversions. The quality of error correction and the size of inserted long-read bases determines the single-base–level accuracy. This affects single-nucleotide polymorphisms and small insertion/deletion calls.

QUAST [41] (version 5.0.2) (QUAST, RRID:SCR_001228) was used to determine length statistics for the assembly such as total length, scaffold NG50, and scaftig NG50, as well as alignment to the reference, including scaffold NGA50, scaftig NGA50, genome fraction, misassemblies, and local misassemblies. To further assess the efficiency and accuracy of TGS-GapCloser, we aligned the reference assembly against the input scaffolds to generate theoretically filled gap sequences using QUAST intermediate files and compared them to the filled sequences by TGS-GapCloser with minimap2 (-x map-ont). Gaps that were capable of being filled by the reference were chosen to evaluate the sensitivity and PPV. Note that gaps smaller than 100 bp were filtered out. The sensitivity is defined as the ratio of the number of TGS-GapCloser–filled gaps that the reference also successfully fills to the total number of gaps that the reference can fill. The PPV is defined as the ratio of the number of TGS-GapCloser–filled gaps that can be uniquely matched to the reference-filled gaps to the total number of filled gaps by both. Note that TGS-GapCloser also completes gaps that the reference cannot fill and as such the accuracy of these cannot be easily determined. The single-base–level accuracy was quantified by mapping the scaftigs in the assembly to the GIAB high-confidence regions in the reference genome GRCh37 to calculate the concordance QV with the method in Wenger et al. [33], where the scaftigs were split into bins of 100 kb, and those bins with >50% mapped length at >50% identity ratio were used to calculate the average concordance quality value. The QVs were expressed in Phred format.

### BUSCO

To quantify the possible improvements for downstream bioinformatics analyses, we ran BUSCO on all the human assemblies against the vertebrata_odb9 and the gingko assemblies against the embryophyta_odb9 database. Note that we directly input the whole human assemblies but split gingko ultra-long scaffolds (>1.1 Gb) into several portions at the position of large gaps (>1 kb) because the aligner tblastn [47] in BUSCO could not handle such long sequences. The additional random breakpoints in the original scaffolds would decrease the contiguity and affect the BUSCO benchmarking.

## Availability of Source Code and Requirements

Project name: TGS-GapCloser

Project home page: https://github.com/BGI-Qingdao/TGS-GapCloser

Operating system(s): Linux

Programming language: C++, shell

Other requirements: Racon, or SAMtools and Pilon are required to be pre-installed

License: GPLv3

RRID:SCR_017633

biotools ID: TGS-GapCloser

Conda access: conda install -c bioconda tgsgapcloser

## Availability of Supporting Data and Materials

The stLFR sequencing data for the human sample (HG001/NA12878) have been deposited in the CNGB under accession No. CNP0000066. We downloaded the ONT long reads of human from [48], and PacBio reads from GIAB [49]. The PacBio long reads for ginkgo genome have been deposited in the CNGB under accession No. CNP0000796 (PRJNA656117). All the evaluated assemblies of human and ginkgo generated by us can be obtained in the CNGB under accession No. CNP0000796. The genome assemblies and all supporting data can be accessed at the *GigaScience* GigaDB database [50].

## Additional Files

**Supplementary Figure S1:** Effects of long-read coverage on gap closure. (A) the number of filled gaps and bases, (B) wall-clock time and peak memory, (C) accuracy in long-read selection, and (D) accuracy at single-base level. All datasets were run with 16 threads.

**Supplementary Figure S2:** Length distribution of gaps in draft scaffolds and that of TGS-GapCloser–filled gap sequences.

**SupplementaryFigure S3:** Read length distribution for the input ONT Rel3 and PacBio HiFi reads.

**Supplementary Table S1:** Summary of the input assemblies in this work.

**Supplementary Table S2:** Summary of the updated assemblies in this work.

**Supplementary Table S3:** The effect of the scoring system on the candidate selection and the gap-closing performance.

**Supplementary Table S4:** The effect of error correction on the candidate selection and the gap-closing performance.

**SupplementaryTable S5:** The effect of long-read coverage on the TGS assemblies and gap-closing results.

**SupplementaryTable S6:** Genomics dataset source.

**Supplementary Table S7:** Control parameters used for different software tools.

giaa094_GIGA-D-20-00014_Original_Submission

giaa094_GIGA-D-20-00014_Revision_1

giaa094_GIGA-D-20-00014_Revision_2

giaa094_Response_to_Reviewer_Comments_Original_Submission

giaa094_Response_to_Reviewer_Comments_Revision_1

giaa094_Reviewer_1_Report_Original_Submission Chong Chu -- 3/19/2020 Reviewed

giaa094_Reviewer_1_Report_Revision_1 Chong Chu -- 6/7/2020 Reviewed

giaa094_Reviewer_2_Report_Original_Submission Justin Chu -- 3/20/2020 Reviewed

giaa094_Reviewer_2_Report_Revision_1 Justin Chu -- 6/10/2020 Reviewed

giaa094_Supplemental_File

## Abbreviations

BLAST: Basic Local Alignment Search Tool; bp: base pairs; BUSCO: Benchmarking Universal Single-Copy Orthologs; Chr19: Chromosome 19; Chr6: Chromosome 6; CNGB: China National GeneBank; CPU: central processing unit; Gb: gigabase pairs; GIAB: Genome in a Bottle; kb: kilobase pairs; Mb: megabase pairs; MHC: major histocompatibility complex; NGS: next-generation sequencing; ONT: Oxford Nanopore Technologies; PacBio: Pacific Biosciences; PPV: positive predictive value; QS: quality score; QV: quality value; SIMD: single-instruction-multiple-data; SLR: synthetic long reads; stLFR: single-tube long fragment reads; TGS: third-generation sequencing.

## Competing interests

All authors are employees of BGI or its subsidiaries. The authors declare that they have no other competing interests.

## Funding

This research was supported by the Shenzhen Municipal Government of China Peacock Plan (No. KQTD2015033017150531), the National Key Research and Development Program of China (Grant No. 2018YFD0900301-05), and the Qingdao Applied Basic Research Projects (Grant No. 19-6-2-33-cg).

## Authors' Contributions

M.X., L.G., and L.D. performed software design and implementation. M.X., L.G., S.G., O.W., and R.Z. contributed to data modeling, data curation, and assembler benchmarking. M.X. wrote the draft manuscript, and L.G., B.A.P., G.F., L.D., Y.Z., X.L., and X.X. contributed to manuscript editing. L.D. and Y.Z. supervised the project. M.X., G.F., and Y.Z. secured funding. All authors read and approved the final manuscript.
