# Supplementary material for: TGS-GapCloser: A fast and accurate gap closer for large genomes with low coverage of error-prone long reads
Source: Gigascience. 2020 Sep 7;9(9):giaa094. doi: 10.1093/gigascience/giaa094 (PMC7476103; doi:10.1093/gigascience/giaa094)
Supplement: giaa094_GIGA-D-20-00014_Original_Submission [file giaa094_giga-d-20-00014_original_submission.pdf]

GigaScience

TGS-GapCloser: A fast and accurate gap closer for large genomes with low coverage of error-prone long reads.

--Manuscript Draft--

**Manuscript Number:**	GIGA-D-20-00014
**Full Title:**	TGS-GapCloser: A fast and accurate gap closer for large genomes with low coverage of error-prone long reads.
**Article Type:**	Technical Note
**Funding Information:**	National Key Research and Development Program of China (2018YFD0900301-05) — Dr. Guangyi Fan<br>Qingdao Applied Basic Research Projects (19-6-2-33-cg) — Dr. Mengyang Xu
**Abstract:**	Background: The continuity, completeness and accuracy of genome assemblies determine the quality of subsequent bioinformatics analysis. Despite benefiting from the medium/long-range information of single molecule sequencing techniques, current gap-closing tools to enhance assemblies suffer multi-alignments and high error rates, resulting in huge time and money costs, especially for large genomes.<br>Findings: We developed a software tool, TGS-GapCloser that uses low-depth (~10×) long reads to close gaps for large genomes. The algorithm extracts long-read gap regions from the alignments against input scaffolds, corrects only the candidate fragments, and assigns the best sequences to each gap. We demonstrate that TGS-GapCloser improves the contig NG50 value of three human genome assemblies by 24-fold on average with only ~10× coverage of ONT or Pacbio reads, completing up to 94.8% gaps with 97.7% positive predictive value. Despite of high error rate of raw long reads, the improved assembly achieves Q46 (99.997%) single-base accuracy, which promises the high-quality downstream analysis, including that up to 13.1% more BUSCO genes are completed. Comparing with mainstream gap-closing tools for third-generation long reads, it finishes ~60% more gaps but runs ~29-fold faster. TGS-GapCloser also shows its power to fill gaps for the ultra large genome assembly of ginkgo (~12Gb) with 71.6% of gaps closed. The validation of inserted sequences was conducted with reference genomes and GIAB benchmark sets.<br>Conclusions: TGS-GapCloser can close gaps of large genome assemblies using long reads in a fast and cost-effective way, and improve the continuity, completeness without loss of accuracy.
**Corresponding Author:**	Mengyang Xu<br>BGI<br>CHINA
**Corresponding Author Secondary Information:**	
**Corresponding Author's Institution:**	BGI
**Corresponding Author's Secondary Institution:**	
**First Author:**	Mengyang Xu
**First Author Secondary Information:**	
**Order of Authors:**	Mengyang Xu<br>Lidong Guo<br>Shengqiang Gu<br>Ou Wang<br>Rui Zhang<br>Guangyi Fan

Powered by Editorial Manager® and ProduXion Manager® from Aries Systems Corporation

	Xun Xu
	Li Deng
	Xin Liu
Order of Authors Secondary Information:	
Additional Information:	
Question	Response
Are you submitting this manuscript to a special series or article collection?	No
**Experimental design and statistics**<br><br>Full details of the experimental design and statistical methods used should be given in the Methods section, as detailed in our Minimum Standards Reporting Checklist. Information essential to interpreting the data presented should be made available in the figure legends.<br><br>Have you included all the information requested in your manuscript?	Yes
**Resources**<br><br>A description of all resources used, including antibodies, cell lines, animals and software tools, with enough information to allow them to be uniquely identified, should be included in the Methods section. Authors are strongly encouraged to cite Research Resource Identifiers (RRIDs) for antibodies, model organisms and tools, where possible.<br><br>Have you included the information requested as detailed in our Minimum Standards Reporting Checklist?	Yes
**Availability of data and materials**<br><br>All datasets and code on which the conclusions of the paper rely must be either included in your submission or deposited in publicly available repositories (where available and ethically	Yes

appropriate), referencing such data using a unique identifier in the references and in the “Availability of Data and Materials” section of your manuscript.<br><br>Have you have met the above requirement as detailed in our Minimum Standards Reporting Checklist?	

Powered by Editorial Manager® and ProduXion Manager® from Aries Systems Corporation

Manuscript

Click here to access/download;Manuscript;TGS-GapCloser_GigaScience_version5.docx

TGS-GapCloser: A fast and accurate gap closer for large genomes with low coverage of error-prone long reads

Mengyang Xu[1,2,4], Lidong Guo[3,1], Shengqiang Gu[3,1], Ou Wang[4,6], Rui Zhang[1], Guangyi Fan[1,4], Xun Xu[4,5], Li Deng[1,2,4, *] & Xin Liu[1,2,4,5, *]

[1]BGI-Qingdao, BGI-Shenzhen, Qingdao 266555, China

[2]State Key Laboratory of Agricultural Genomics, BGI-Shenzhen, Shenzhen 518083, China

[3]BGI Education Center, University of Chinese Academy of Sciences, Shenzhen 518083, China

[4]BGI-Shenzhen, Shenzhen 518083, China

[5]China National GeneBank, BGI-Shenzhen, Shenzhen 518120, China

[6]MGI, BGI-Shenzhen, Shenzhen 518083, China

*Corresponding authors: Li Deng (dengli1@genomics.cn) and Xin Liu (liuxin@genomics.cn)

Abstract

Background: The continuity, completeness and accuracy of genome assemblies determine the quality of subsequent bioinformatics analysis. Despite benefiting from the medium/long-range information of single molecule sequencing techniques, current gap-closing tools to enhance assemblies suffer multi-alignments and high error rates, resulting in huge time and money costs, especially for large genomes.

Findings: We developed a software tool, TGS-GapCloser that uses low-depth (~10×) long reads to close gaps for large genomes. The algorithm extracts long-read gap regions from the alignments against input scaffolds, corrects only the candidate fragments, and assigns the best sequences to each gap. We demonstrate that TGS-GapCloser improves the contig NG50 value of three human genome assemblies by 24-fold on average with only ~10× coverage of ONT or Pacbio reads, completing up to 94.8% gaps with 97.7% positive predictive value. Despite of high error rate of raw long reads, the improved assembly achieves Q46 (99.997%) single-base accuracy, which promises the high-quality downstream analysis, including that up to 13.1% more BUSCO genes are completed. Comparing with mainstream gap-closing tools for third-generation long reads, it finishes ~60% more gaps but runs ~29-fold faster. TGS-GapCloser also shows its power to fill gaps for the ultra large genome assembly of ginkgo (~12Gb) with 71.6% of gaps closed. The validation of inserted sequences was conducted with reference genomes and GIAB benchmark sets.

Conclusions: TGS-GapCloser can close gaps of large genome assemblies using long reads in a fast and cost-effective way, and improve the continuity, completeness

without loss of accuracy.

Keywords: gap-closure, third-generation sequencing, genome assembly, ginkgo, MHC

Findings

Introduction

The development of genome sequencing techniques has been reducing the cost and improving the throughput at a speed beyond the Moore's Law over the last decade[1]. The genetic sequence databases have been drastically enriched, and progressively increasing focuses move from small bacterial and fungal genomes to large eukaryotes. The applications of state-of-the-art techniques, for instance, TGS long reads[2, 3], SLR libraries[4-8], Hi-C[9], and BioNano physical map[10], provide extra information on different length scales to increase negentropy of the system, resulting in the enhancements of genome assemblies. However, all the finished assemblies are imperfect, even for human and model organisms, which contain gaps of unknown nucleic acids (represented by Ns). The repetitiveness and polymorphism of the genomes, the limitation of sequencing techniques, and the trade-off of algorithms may lead to the gaps. Gap closure or gap filling can discover the sequences and extend contigs to entirely or partially missing gene-encoding area to bridge the gaps. Therefore, there has been a need to develop tools to close gaps in *de novo* assemblies

for more complete genomes, especially for large eukaryotic genomes with high complexity.

The first effort to finish gaps in draft genome assemblies was made using Fosmids, BACs libraries and Sanger reads for a large gap range of 1 to 100 kb[11]. But the manual or semi-automated processes limit the applications in consideration of huge costs. The NGS technologies along with paired-end and mate-pair information of multiple insert sizes have overcome the financial problem, and several landmark tools have been designed to reach into gap regions [12-16], sharing similar kmer-extension or local reassembly algorithms, but suffering the same problem of CPU and memory consuming for large genomes. Besides, those strategies cannot span the repetitive DNA fragments such as tandem repeats, and cause more misassemblies due to the short read/kmer length.

The single molecule TGS technologies, including Pacbio and ONT, have the potential to solve these limitations as their reads (~10kb) are typically longer than most DNA repeats[17]. Although the *de novo* genome assembly using long reads may allow incremental improvements, the higher expense and lower accuracy relative to NGS platforms prevent the popularization, which generally requires intense computational costs for long-read error correction. These common TGS sequencing errors, including insertions and deletions, may cause frameshifts in gene-coding regions, disrupting the protein prediction[18]. There have been several hybrid assemblers designed to combine advantages of both sequencing platforms since the TGS commercial techniques were released. Main principles include constructing a final assembly graph

by mixing NGS contigs and TGS long reads based on the OLC or string graph algorithm[19], or scaffolding the contigs generated by NGS dependent on their alignments against long reads[20-22] to utilize the medium/long-range information, but ignore the possible combination with longer assembly information provided by other techniques. Gap-closing algorithms, however, only upgrade the missing regions, reserving the majority of the existing assembly information to considerably reduce the computing complexity and cost. PBJelly[23] was the first software tool to use Pacbio datasets to close gaps through locally assembling the mapped long reads in gap regions. The number of gaps could also be efficiently reduced by FGAP[24], which aligned long reads to gaps using BLAST algorithm[25]. More tools modified and extended the algorithms for different purposes[26-31]. However, most tools mentioned above share the same crucial shortcoming: they function well only with pre-error-corrected or simulated long reads. It hampers the application because the error correction needs sufficient coverage of expensive long reads or extra short reads, and requires huge time and memory consumption, but usually splits long reads into short fragments and loses valuable assembly length information, not readily usable for large genomes.

It is necessary to comprehensively utilize assembly information in short range (NGS, <1kb), medium range (TGS, 1k~10kb) and long range (TGS, SLR, Hi-C, BioNano, 10kb~Mb) with different resolutions, and balance their costs. Three key points should be considered to develop a TGS gap-closing algorithm. First, use TGS data as few as possible. Although the price has been decreasing[32], the data efficiency is still the

first priority, especially for those small labs or small projects. Thus, local reassembly or pre-error correction through overlapping long reads is not preferable. Another important factor is the accuracy and precision in the choice of long reads to fill the gaps. It has been demonstrated that the number of assembly errors caused by most gap-closing tools is higher than that of *de novo* assembled contigs[27]. High error rate and the existence of repeats may increase the probability of large misassembly events. Last but not least, the filled gaps should not diminish the single-base level accuracy, which influences the quality of downstream analysis. There is still a need of error correction or polish for inserted raw long read sequences. Note that the most recent Pacbio improved its base-calling accuracy to 99.8%[33], which may directly simplify the problem, but sacrifice the throughout and read length, affecting the gap-closure efficiency.

In this work, we describe a software tool, named TGS-GapCloser, that uses error-prone long reads at low depth to efficiently and accurately close gaps for large genomes within a reasonable time. We applied it to three draft *de novo* assemblies of human genome using ONT or Pacbio long reads[34, 35], improving the contig NG50 10.9 to 44.9-fold and NGA50 6.9 to 30.7-fold dependent on different inputs, finishing ~ 90% gaps on average with 88.2% PPV and 77.9% sensitivity. The improved human genome assemblies achieve up to Q46 (99.997%) single-base accuracy, beyond that of most long read assemblies[33, 34]. In addition, the number of BUSCO completed genes is increased by 3.3-13.1%, respectively, implying the updates of completeness and accuracy. 71.6% gaps in the ultra large genome assembly of ginkgo are also

closed using 10.5× coverage of corrected Pacbio long reads, increasing the contig N50 from 48kb to 365kb. The updated genome assemblies generated by *de novo* assemblers with TGS-GapCloser as a hybrid strategy may promote the quality of gene annotation, structure variation calling and thus improving the quality of downstream analysis of ontogeny, phylogeny, and evolution.

Data description

Three datasets of two large genomes were used to examine the gap-closing results by TGS-GapCloser. We sequenced *Homo sapiens* (HG001/NA12878) using MGIEasy stLFR Library Prep Kit on BGISEQ-500 platform with the data size of 660 Gb, and reads mapped to the Chr19 were also extracted for comparisons and further analysis. NGS short reads were assembled using MaSuRCA (version 3.3.1)[21] or Mercedes (in-house tool) to obtain short but highly accurate contigs for each dataset, and the SLR long-range (barcode/read cloud) and short-range (paired-end) information provided by stLFR technique was exploited to do further scaffolding by SLR-superscaffolder(version 1.0.0)[36]. Supernova (version 2.1.1)[37] was originally designed to assemble 10X Genomics data, but could be applied to stLFR format reads to obtain draft scaffolds. To test the generalization and potential of TGS-GapCloser, we utilized new generations of long reads for the human genome to close gaps: ONT MinION rel3 dataset (rel3)[34] and Pacbio CCS HiFi dataset (HiFi)[35]. Note that currently there has been no HiFi reads for HG001/NA12878 available, and the dataset for HG002/NA24385 was applied instead.

The input genome assembly of *Ginkgo biloba* female (estimated about 12 Gb) was obtained from [38], which was initially assembled using SOAPdenovo2[12] and updated using Hi-C data[39]. The Pacbio reads for ginkgo were sequenced by Pacbio Sequel, with the chemistry of Sequel Sequencing Kit 3.0 Bundle (4 rxn). The total data amount was 256 Gb with the average read length of 38,623 bp. Error correction by Canu[40] reduced data size to 126 Gb, with the average read length of 10,722 bp. The basic statistics for input assemblies are listed in Table S1, and sequencing reads are in Table S3.

Algorithm and implementation of TGS-GapCloser

TGS-GapCloser accepts any type of TGS long reads or other pre-assembled contigs to automatically fill gaps in any type of draft assemblies in the following four steps as shown in Figure 1: (i) determination of gap regions in the draft assembly; (ii) acquisition of candidates from the alignments of long reads against gaps; (iii) base-level error correction of alternative sub-long reads; and (iv) gap closure using the error-corrected candidates with the highest score for each gap or linkage of the neighboring contigs with overlaps.

Figure 1. A schematic of TGS-GapCloser workflow. (A) A flow chart of the overall algorithm, (B) a schematic describing the determination of gap regions, acquisition of candidate long read fragments, and error correction of alternative sub-long reads, (C) a detailed flow chart for gap filling or contig merging in a gap region with the most proper

medium/long-range information from long reads.

The input scaffolds were firstly split into parts called contigs (scaftigs) from the observed N positions, and each pair of neighboring contigs according to their positions in the shared scaffold were considered as a gap region waiting to be filled. TGS-GapCloser defaults the high quality of inputs, including the base-level accuracy, the order and orientation of contigs, but not the estimated gap size. That is because the estimations based on the long-range information provided by SLR, Hi-C, or BioNano barely reach low resolutions (<10kb), leading to a high probability of faults especially for small gaps.

We used minimap2[41] to align long reads against each gap region to obtain corresponding candidate fragments with the preset options for ONT or Pacbio datasets. A candidate for a specific gap is defined as the segment truncated from the aligned long reads in the area between two neighboring contigs along with 2kb flanking wings on both sides. Each long read might provide several candidate sequences dependent on its spanning length and base-calling accuracy, but was limited to give at most one candidate for the same gap region to overcome the redundant alignments due to the algorithm’s nature of the aligner and high error rate of long reads.

The alignment amount and quality determine the efficiency and accuracy of gap closure. Thus, all alignments were filtered based on the alignment length and identity ratio. For each gap, up to ten sub-long reads with the highest QS were chosen as

candidates for error correction, avoiding multi-alignments within the same area and dramatically suppressing the data amount for further analysis. The QS is given by

$$QS = a \cdot logA_i + b \cdot logI_i + a \cdot logA_{i+1} + b \cdot logI_{i+1}$$

where letter *A* refers to the alignment length, letter *I* refers to the identity ratio for *i*th, *i+1*th contigs respectively in preliminary scaffolds; letter *a* and *b* are two arbitrary coefficients to distinguish *A* and *I*'s weights on the score, and have been tuned to 1:6 for ONT dataset as default. To further reduce the complexity and save computational resources, the overlapped candidates in the same long read were clipped and merged for the following error correction.

The merged candidate sequences could be corrected using Pilon[42] or Racon[43] to enhance both the base-level accuracy and the precision of alignments. Pilon was utilized to fix individual base errors, small indels and local misassemblies with short but accurate NGS reads, while Racon was used to correct sequencing errors by constructing a SIMD-accelerated partial-order alignment graph from long read's overlaps. The short reads were aligned to the candidates also using minimap2 but conducted with the option *-k14 -w5 -n2 -m20 -s40 --sr --frag yes* tuned for short sequences. The choice of using Pilon or Racon or neither depends on the type of input reads. The utilization of Racon runs faster than Pilon in most of our tests but has limited effects on the accuracy of corrected fragments.

The error correction would benefit not only the single-base accuracy but also the final choice of candidates to fill gaps on the basis of the hypothesis that a candidate with higher-quality alignments could be mapped to a more precise position in the reference

after error correction, while the one with lower-quality alignments became to fail to be mapped. The error-corrected candidates were again split and aligned against their corresponding gaps, and finally the one with the highest QS would be employed to fill the gap. We discarded the flanking wings of winning candidates but used bases from contigs as many as possible considering the relatively lower accuracy of long reads. If the winning candidate gave a negative filling information instead, then the gap region would collapse to a single contig based on the overlapping relation. A portion of contigs have overlaps with others because of incorrect paths in the initial assembly graph or too aggressive contig extension strategy. However, a single molecule long read spanning the gap has the ability to solve the overlapping if two contigs are mapped to the correct positions. We took more care of “negative” gaps given by the best corrected candidates with extra strict criteria because most base-calling errors in long reads, including indels and homopoly-meric repeats tend to cause untruthful overlapping. Gaps without any corresponding candidates would fail to be closed, possibly because of misassemblies in the draft assembly.

TGS-GapCloser is coded in C++ programing language (requires GCC 4.4+ and Make 3.8+). It applies minimap2 to align long reads against gaps or short/long reads against candidates, and Pilon (requires Java runtime 1.7+) or Racon (requires GCC 4.8+ and CMake 3.2+) to correct errors. The acceleration and enhanced mapping quality of the algorithm partially originate from the aligner, as minimap2 shows great improvements in speed and overall higher mapping accuracy for error-prone long reads[41]. The algorithm automatically determines gap areas and tries to find the best matched long

read fragments to fill gaps or merge adjacent contigs based on the alignments. The details in each step are individually recorded, including gap determination, mapping, long read extraction, error correction, and gap filling or merging. The final output is reported in FASTA format, along with log files describing the detailed sequence insertion/merging information for each gap to trace all the improvements. TGS-GapCloser is available via GitHub at https://github.com/BGI-Qingdao/TGS-GapCloser.

Gap closure in human genome

Three genome assemblies and two long read datasets were used to benchmark the utility of TGS-GapCloser in gap closing or contig merging in preliminary scaffolds for *Homo sapiens*. Using the same library, the whole genome was assembled by: (1) contigs by MaSuRCA + scaffolds by SLR-superscaffolder, (2) contigs by Mercedes + scaffolds by SLR-superscaffolder, and (3) contigs and scaffolds by Supernova to take full use of barcoded long-range information. Although MaSuRCA itself can assemble both contigs and scaffolds, the lack of SLR information used in the assembler results in relatively short scaffolds. It is necessary to employ SLR-superscaffolder to obtain a comparable scaffold NG50 against Supernova. TGS long reads are ideal to build the bridge between NGS reads and barcodes with respect to the length and resolution of assembly information. To assess the efficient usage of long reads, we randomly extracted only ~10× coverage long reads from ONT rel3 dataset with the claimed mean read identity of 82.73%[34] and Pacbio HiFi dataset with the claimed average

read concordance of 99.8%[33], respectively. The long-read fragments from rel3 were corrected by Pilon with NGS short reads while those from HiFi were corrected by Racon with long reads themselves. Gap regions in draft assemblies have been investigated that they can be fully covered by at least one long reads. Figure 2 describes the improvements in the assembly evaluation given by QUAST[44] after gap closure with our method. The contig NG50 increases from 13.2kb to 593.4kb with rel3 and to 222.4kb with HiFi for Assembly (1), 15.3kb to 660.7kb with rel3 and to 206.5kb with HiFi for Assembly (2), and 109.6kb to 1193.4kb with rel3 and to 1541.9kb with HiFi for Assembly (3); while the corresponding contig NGA50 grows from 13.1kb to 402.0kb with rel3 and to 192.9kb with HiFi, 15.2kb to 405.7kb with rel3 and to 177.4kb with HiFi, and 105.5kb to 724.9kb with rel3 and to 838.7kb with HiFi, respectively. Note that our current algorithm does not split or merge input scaffolds to remain the existing long-range information. Up to 91.8% of total 191189, 94.8% of total 129408, and 86.9% of total 42359 gaps are successfully finished by TGS-GapCloser for three assemblies. As listed in Table S2, the genome fraction against the reference is also improved by 1.6%, 3.7% and 0.5% using rel3, 1.5%, 2.5% and 0.5% using HiFi for different inputs after gap closure, indicating that the updated gap regions are mapped to the reference's new areas. The application of rel3 dataset increases the large-scale misassemblies (>1kb) induced by the filled sequences by 17.4% and 9.7% in Assembly (2) and (3) as expected, but decreases by 10.7% in Assembly (1) due to the more precise mapping position of scaffolds/contigs against the reference. In spite of error correction, the local misassemblies (<1kb) still present

an increment of 1.2-fold, 7.4-fold, and 1.1-fold dependent on the length and the single-base accuracy of filled sequences. The HiFi dataset with higher initial read accuracy represents fewer induced misassemblies or local misassemblies: -6.7% and 0.3-fold for Assembly (1), 13.7% and 1.4-fold for Assembly (2), 20.1% and 0.5-fold for Assembly (3). Overall, rel3 closes more gaps to obtain better continuity than HiFi, but induces more assembly errors. This is because there are longer reads in the ONT rel3 dataset (the longest >500kb) while Pacbio HiFi folds the single-molecule fragments into ~13kb subreads to achieve better single-base accuracy (Figure S3). The performance of TGS-GapCloser is substantially dependent on both the length and the accuracy of long reads, which are weights on each side of the balance for current single-molecule sequencing techniques.

BUSCO[45] (version 3.0.2) analysis indicates the possible enhancements for further analysis such as gene annotation after gap filling. The genome was queried against the vertebrata_odb9 database. It reveals that 90.5%, 89.7% and 94.1% of the expected vertebrate genes are complete after gap filling using rel3, and 90.9%, 86.5% and 94.0% using HiFi, both improved from the original 86.2%, 76.6% and 90.7%, respectively.

Figure 2. Gap filling improvements and effects on the draft assemblies produced by TGS-GapCloser. (A) contig NG50, (B) contig NGA50, (C) number of remaining gaps, (D) genome fraction, (E) misassemblies and (F) local misassemblies for the human genome. (C) was given by directly counting while others were reported by QUAST.

Gap closure in the ultra large genome of ginkgo

The gingko is a best-known living fossil that has remained its form and structure over 270 million years, taking a unique position in the evolutionary tree of life[47]. We applied TGS-GapCloser to improve the chromosomal-level assembly of the *Ginkgo biloba*[38] by incorporating 10.5× coverage of error-corrected Pacbio data. The input assembly has been assigned to13 chromosomes of 9,570,195,624 bp, with 613,821 gaps in total. In this case, the long reads are pre-corrected by Canu. After gap filling which only consumed 541 CPU hours, up to 71.6% of the gaps were closed, and thus the total contig size was increased by 411,608,879 bp, 4.3% of total. The contig N50 was also enhanced from 57.1kb to 364.8kb. Note that most gap-closing tools have been benchmarked only for several bacterial and fungal genomes or small eukaryotes previously[24, 27, 29], and it is doubt that they could be applied to this ultra large genome using reasonable computing resources.

Validation of gap-closing sequences

As a sanity check, we generated ideally filled sequences for all gaps using the human reference genome and compared them to the gap-filling sequences created by TGS-GapCloser. Note that the statistics of filled gaps described here would be different from that given by directly counting (Figure 2 (C)) since the situations with overlapping are not considered. Table 1 lists the evaluation of TGS-GapCloser’s improvements. By comparing the fragments inserted by TGS-GapCloser to those

validated by the reference, the PPV ranges from 98.4% to 62.0% for three assemblies using two different long-read datasets, and the sensitivity from 96.3% to 51.2%. In total, gap-closing results using HiFi reads show relatively higher PPV and sensitivity due to its higher read accuracy. The accuracy in long-read selection of Assembly (1) or (2) is better than that of Assembly (3) where short length predominates in the input gaps, which implies that TGS-GapCloser tends to fill large gaps as it is more difficult for long reads to be mapped to short gap regions.

In terms of single-base level accuracy, the Phred-like concordance QV calculated with the same method described in [33] is decreased by the inserted sequences: the contig QV down from 45.9 to 40.7 using rel3 and to 41.4 using HiFi, with the filled-sequence QV of 23.2 using rel3 and 32.6 using HiFi after error correction on average. The more accurate HiFi reads more slightly decrease the single-base accuracy, which is consistent with the accuracy in long-read selection. The final assemblies with >Q40 single-base quality, which are comparable with or better than TGS long read *de novo* assemblies along with error correction and polish[33, 34], satisfy the so-called "3.4.2Q40" perfect genome assembly standard (contig N50 >1Mb, scaffold N50 >10Mb, contigs mapped to chromosomes >90%, and consensus accuracy >Q40)[48].

Table 1. Gap Filling accuracy statistics and computational consumptions for TGS-GapCloser.

Performance of TGS-GapCloser for large genomes

We have presented a new tool for updating a draft genome assembly based on currently available long reads fast and accurately. With respect to the human genome, it consumes as low as 155 CPU hours in total and 32 GB peak memory. It only corrects the fragments in gap regions, substantially reducing the time for mapping and error correction (>90% of the whole pipeline). The usage of short reads to correct fragments by Pilon (~7 days 21 hours on average) is slower than the corrections based on long read's overlaps by Racon (~15 hours on average). The gap-closing algorithm is more costly effective with higher assembly continuity and accuracy compared with the *de novo* assemblies of 30× coverage of long reads, which requires ~40K CPU hours for ONT and ~62K CPU hours for Pacbio[34].

The time and memory usage are further reduced without error correction. It takes only 541 CPU hours for ginkgo's ultra large genome using pre-error-corrected Pacbio reads.

This method is widely applicable to many genome projects for thousands of research groups, benefiting from its flexibility of using different sequencing technologies and different assemblies. TGS-GapCloser requires only low depth of expensive long reads without pre-error correction, making this approach more costly effective and suitable for small budgets.

Comparison with other tools

We did not compare TGS-GapCloser to NGS gap-closing tools because the utilization

of medium/long-range information provided by long reads spans repetitive or other complicated regions that kmer-based extension cannot reach and congenitally creates better results as revealed in previous studies[24, 27]. In this work, we chose PBJelly and FGAP, and applied to the Chr19 Mercedes+SLR-superscaffolder assembly with the rel3 dataset to compare the gap-closing performance. Other subsequent tools do not display obvious improvements in efficiency and accuracy of gap filling[29, 31]. The stLFR short reads were also extracted for Chr19, and used to correct long read fragments by TGS-GapCloser. Note that for the human whole genome FGAP failed to close gaps during our test due to its huge memory requirement (>1.5 TB), while PBJelly stopped running with no outputs after 30 days for unclear reasons.

The evaluation of outputs shows that the gap-closing efficiency of TGS-GapCloser is considerably higher than that of other tools, leaving only 322 gaps after gap closure compared to 1,730 for PBJelly default, 458 gaps for FGAP default and 782 for FGAP with overlap option on (Table 2), thus enhancing the contig NG50 and NGA50 from 9.7kb to 161.6kb, and 9.5kb to 117.6kb, respectively, 5.8- and 3.2-fold than PBJelly and FGAP. The induced misassemblies are less than that of PBJelly or FGAP default but comparable with FGAP (overlap on), whereas FGAP (overlap on) represents the least local misassemblies. Considering the common existence of overlapped adjacent contigs in input scaffolds, the overall result of FGAP with overlap option on is better than that of default settings.

Table 2. Gap filling statistics for TGS-GapCloser, PBJelly and FGAP.

Input data	Unfilled gaps	Misassembly	Local misassembly	Scaffold NG50 (bp)	Scaffold NGA50 (bp)	Contig NG50 (bp)	Contig NGA50 (bp)	Runtime (hours)	Peak memory (GB)
Draft Assemblies	2,600	176	126	1,561,142	196,307	9,687	9,464	/	/
TGS-GapCloser	322	210	1,035	1,418,496	274,922	161,606	117,601	2.1	8.22
PBJelly	1,730	664	741	1,240,439	83,803	29,715	19,247	52.3	9.93
FGAP	458	867	684	1,871,611	44,244	127,982	28,615	44.8	35.06
FGAP (overlap on)	782	204	292	1,536,120	204,672	39,581	34,514	63.4	36.04

All datasets were run with 16 threads. Note that the memory consumption by Pilon is not counted.

In addition, we measured the running time and memory consumption of each tool under the same operation conditions. TGS-GapCloser surprisingly runs approximately 20- and 40-fold faster than PBJelly and FGAP. The maximal memory used by TGS-GapCloser is also comparably the smallest.

Effects of long read coverage

It is worthwhile evaluating the effects of long read coverage on gap filling results. We

extracted 1×, 5×, 10×, 20× and original 29× coverages of mapped ONT rel3 reads against the Chr19 reference, and individually applied them to close gaps in the same Chr19 Mercedes+SLR-superscaffolder assembly by TGS-GapCloser using the same default options. As shown in Figure S1 (A), the number of filled gaps and total filled bases grow with the increasing coverage, but saturate at 10× coverage to the level of theoretically filled gap number and bases. Surprisingly, the total time usage does not change much as the coverage increases, while the peak memory presents an approximately linear growth in Figure S1 (B). With more long reads, Figure S1 (C) displays that the sensitivity increases from 22.1% to 87.4% while the PPV remains similar as expected. In terms of single-base level accuracy in Figure S1 (D), the concordance QV of inserted sequences drops as more gaps are closed, but has ignorable effect on that of output contigs. The result indicates that TGS-GapCloser enables a considerable number of gaps closed with high quality using low coverage of long reads.

Improvements in MHC region

The assembly of MHC region in the human genome has been advertised as a proof that TGS medium/long-range information overcomes the assembly difficulty of short reads to span the 6-Mb region of high repetition and polymorphism[34]. It is located on the Chromosome 6 containing all class I and II human leukocyte antigens genes, important to cancer and immunity studies[46]. We analyzed three assemblies to investigate the contiguity and accuracy in this region as shown in Table 3. For

Assembly (3), a portion of a single scaffold (>29Mb) entirely covers the MHC region, while portions of two and three scaffolds (0.6-27Mb) cover for Assembly (1) and (2). Gap filling with the ONT rel3 dataset merges contigs from 339 to 31, 271 to 26, and 76 to12, respectively, eliminates N bases from 15.2% down to 3.7% on average, and enhances the genome fraction from 81.52% to 91.17%. As a result, the contig NG50 and NGA50 are also improved from 46.7kb to 585.1kb, and 41.0kb to 300.4kb, which would effect the gene annotations, structural variation detection or single nucleotide polymorphism calling. In contrast, although ultra-long TGS reads resolve the MHC locus into a single or several contigs, the relatively low contig NGA50 (52.6kb), low genome fraction (59.86%) and numerous local misassemblies indicate that the accuracy in short-range information is still a challenge for TGS applications.

Table 3. Enhancements in MHC region assembly by TGS-GapCloser.

Future direction

A number of improvements for future versions of TGS-GapCloser have been put on the agenda. The accuracy of inserted sequences largely depends on the performance of the aligner. Minimap2 performs well in most cases, however, gets worse when handling long sequences sharing short overlaps and short reads against long sequences although it has been tuned somewhat. We hope that the problem will be solved by applying other aligners or sufficient parameter tuning. In addition, the computational consumption by error correctors or polishers is still considerable

although our algorithm has tried to reduce the input data size as much as possible. But it is convenient to replace them with other error-correction tools when available. Long reads with higher quality are promised by ONT and Pacbio, which help us get rid of this annoying step. Last but not least, we default the input scaffolds including the orientation and order relations of contigs to retain the existing assembly information, but neglect previous assembly errors in reality. We are planning to use the medium/ long-range information provided by TGS reads to correct improper relations of contigs in the shared scaffold and link different scaffolds if there is overlapping. Nevertheless, a hybrid assembly strategy using different sequencing libraries with different length and resolution in assembly information including this tool makes it possible to complete the high-quality (ultra) large genome assemblies.

Methods

Gap closing with other tools

We compared the performance of TGS-GapCloser with two state-of-the-art gap-closing tools, PBJelly (version PBSuite_15.8.24) and FGAP (version 1.8.1) using the same human Chr19 assembly and rel3 dataset. For PBJelly, the gaps were closed with default options, while FGAP was conducted with default options and the overlap detection option off and on.

Validation of gap-closing results

We classified the elevation of the gap-closing accuracy in two levels: the accuracy in long-read selection and that in single-base level. The former is determined by whether the algorithm can capture the best matched long read to the corresponding gap region, and effects the medium/long-range information, such as chromosomal variations and large relocations or inversions in the same chromosome. The quality of error correction and the length of inserted long read fragments decide the single-base level accuracy, and effects small single-nucleotide polymorphisms or insertion/deletion calling in short range.

QUAST[44] (version 5.0.2) not only reports basic statistics about the assembly such as total length, scaffold NG50 and contig NG50, but also provides the mapping relations with the reference genome when provided, including scaffold NGA50, contig NGA50, genome fraction, misassemblies and local misassemblies. For each assembly, we used flag -s to split scaffolds into contigs (scaftigs) to evaluate both. To further assess the efficiency and accuracy, we aligned the reference against the input assembly to get the theoretically filled gap sequences with QUAST intermediate files, and compared them to sequences filled by TGS-GapCloser with minimap2 (preset *-x map-ont*). Gaps that can be theoretically filled by the reference are chosen to evaluate the sensitivity and the PPV. Note that sequences shorter than 100bp were filtered out. The sensitivity is defined as the ratio of the number of actually filled gaps that the reference also successively fills to the total number of gaps that the reference can fill. The PPV is defined as the ratio of the number of actually filled gap sequences that can be uniquely matched to the reference-filled gap sequences to the total number of filled

gaps. Note that TGS-GapCloser also completes gaps that the reference cannot fill using the new medium/long-range information provided by TGS long reads, which cannot be easily judged. The single-base level accuracy was quantified by mapping the contigs in the assembly to the GIAB high-confidence regions in the reference genome GRCh38.p13 to evaluate the concordance QV with the method in [33], where the contigs were split into bins of 100kb, and those bins with over 50% length that can be mapped to the high-confidence regions at >50% identity ratio were used to calculate the average concordance quality values. The QVs were expressed in Phred format.

BUSCO

To quantify the possible improvements for downstream bioinformatics analysis, we ran BUSCO analysis for all the assemblies against the vertebrata_odb9 database. Note that we employed all the human genome sequences directly, but split gingko ultra-long scaffolds (>1.1Gb) into several portions at the position of large N gaps (>1kb) because the aligner BLAST implemented in BUSCO could not handle such long sequences. This estimate is a lower bound as additional random breakpoints in the scaffolds decrease the continuity.

Availability of source code and requirements

Project name: TGS-GapCloser

Project home page: https://github.com/BGI-Qingdao/TGS-GapCloser

Operating system(s): Linux

Programming language: C++, shell

Other requirements: Racon, or SAMtools and Pilon are required to be pre-installed

License: GPLv3

RRID: SCR_017633

Availability of supporting data and materials

The human genome datasets and assemblies are available at:

stLFR reads: CNSA under accession ID CNP0000066;

ONT reads: https://github.com/nanopore-wgs-consortium/NA12878/blob/master/nanopore-human-genome/rel_3_4.md

Pacbio reads: NCBI SRA under accession ID SRX5327410

Assemblies: CNSA under accession ID CNP0000796.

The ginkgo genome datasets and assemblies are available at CNSA under accession ID CNP0000796.

Additional files

Supplementary information contains the following information:

Figure S1: Effects of long read coverage on gap closure. (A) the number of filled gaps and

bases, (B) wall-clock time and peak memory, (C) accuracy in long-read selection, and (D) accuracy in single-base level. All datasets were run with 16 threads.

Figure S2. Length distribution of gaps in draft scaffolds and updated versions filled by TGS-GapCloser.

Figure S3. Read length distribution for input ONT rel3 and Pacbio HiFi datasets.

Table S1. Summary of the input contigs/scaffolds in this work.

Table S2. Summary of the updated contigs/scaffolds in this work.

Table S3. Genomics dataset source.

Table S4. Control parameters used for different datasets.

Abbreviations

TGS: third-generation sequencing; GIAB: Genome in a Bottle; SLR: synthetic long reads; NGS: next-generation sequencing; Pacbio: Pacific Biosciences; ONT: Oxford Nanopore Techniques; OLC: Overlap-Layout-Consensus; PPV: positive predictive value; MHC: major histocompatibility complex; stLFR: single tube Long Fragment Reads; Chr19: Chromosome 19; QS: quality score; SIMD: single-instruction-multiple-data; BUSCO: Benchmarking Universal Single-Copy Orthologs; QV: quality value; CNSA: CNGB Nucleotide Sequence Archive.

Competing interests

The authors declare that they have no competing interests.

Funding

This research was supported by the National Key Research and Development Program of China (Grant No. 2018YFD0900301-05) and the Qingdao Applied Basic Research Projects (Grant No. 19-6-2-33-cg).

Authors' contributions

M.X., L.G., and L.D. performed software design and implementation. M.X., L.G., S.G., O.W., and R.Z. contributed to data modeling, data curation, assembler benchmarking. M.X. wrote the draft manuscript and L.G., G.F., and L.D. contributed to manuscript editing. X.X., L.D., and X.L. supervised the project. M.X. and F.G. performed funding acquisition. All authors read and approved the final manuscript.

ACKNOWLEDGEMENTS

The authors are grateful for the advice from Hongmei Zhu, the support of Mercedes Assembler from Yinlong Xie and many other BGI-Shenzhen employees in the development of TGS-GapCloser.

References

1. KA. W. DNA Sequencing Costs: Data from the NHGRI Genome Sequencing Program (GSP). 2014.

2. Branton D, Deamer DW, Marziali A, Bayley H, Benner SA, Butler T, et al. The potential and challenges of nanopore sequencing. Nat Biotechnol. 2008;26 10:1146-53. doi:10.1038/nbt.1495.

3. Schadt EE, Turner S and Kasarskis A. A window into third-generation sequencing. Hum Mol Genet. 2010;19 R2:R227-40. doi:10.1093/hmg/ddq416.

4. Peters BA, Kermani BG, Sparks AB, Alferov O, Hong P, Alexeev A, et al. Accurate whole-genome sequencing and haplotyping from 10 to 20 human cells. Nature. 2012;487 7406:190-5. doi:10.1038/nature11236.

5. Kaper F, Swamy S, Klotzle B, Munchel S, Cottrell J, Bibikova M, et al. Whole-genome haplotyping by dilution, amplification, and sequencing. Proc Natl Acad Sci U S A. 2013;110 14:5552-7. doi:10.1073/pnas.1218696110.

6. Amini S, Pushkarev D, Christiansen L, Kostem E, Royce T, Turk C, et al. Haplotype-resolved whole-genome sequencing by contiguity-preserving transposition and combinatorial indexing. Nat Genet. 2014;46 12:1343-9. doi:10.1038/ng.3119.

7. Zheng GX, Lau BT, Schnall-Levin M, Jarosz M, Bell JM, Hindson CM, et al. Haplotyping germline and cancer genomes with high-throughput linked-read sequencing. Nat Biotechnol. 2016;34 3:303-11. doi:10.1038/nbt.3432.

8. Wang O, Chin R, Cheng X, Wu MKY, Mao Q, Tang J, et al. Efficient and unique cobarcoding

of second-generation sequencing reads from long DNA molecules enabling cost-effective and accurate sequencing, haplotyping, and de novo assembly. Genome Res. 2019;29 5:798-808. doi:10.1101/gr.245126.118.

9. Belton JM, McCord RP, Gibcus JH, Naumova N, Zhan Y and Dekker J. Hi-C: a comprehensive technique to capture the conformation of genomes. Methods. 2012;58 3:268-76. doi:10.1016/j.ymeth.2012.05.001.

10. Shelton JM, Coleman MC, Herndon N, Lu N, Lam ET, Anantharaman T, et al. Tools and pipelines for BioNano data: molecule assembly pipeline and FASTA super scaffolding tool. BMC Genomics. 2015;16:734. doi:10.1186/s12864-015-1911-8.

11. Adams MD FC, Venter JC. Automated DNA Sequencing and Analysis Techniques. Academic Press; 1994.

12. Luo R, Liu B, Xie Y, Li Z, Huang W, Yuan J, et al. SOAPdenovo2: an empirically improved memory-efficient short-read de novo assembler. Gigascience. 2012;1 1:18. doi:10.1186/2047-217x-1-18.

13. Boetzer M and Pirovano W. Toward almost closed genomes with GapFiller. Genome Biol. 2012;13 6:R56. doi:10.1186/gb-2012-13-6-r56.

14. Tsai IJ, Otto TD and Berriman M. Improving draft assemblies by iterative mapping and assembly of short reads to eliminate gaps. Genome Biol. 2010;11 4:R41. doi:10.1186/gb-2010-11-4-r41.

15. Gao S, Bertrand D and Nagarajan N. FinIS: Improved in silico Finishing Using an Exact Quadratic Programming Formulation. In: Berlin, Heidelberg, 2012, pp.314-25. Springer Berlin Heidelberg.

16. Puranik R, Quan G, Werner J, Zhou R and Xu Z. A pipeline for completing bacterial genomes using in silico and wet lab approaches. BMC Genomics. 2015;16 Suppl 3 Suppl 3:S7. doi:10.1186/1471-2164-16-s3-s7.

17. Catasti P, Chen X, Mariappan SV, Bradbury EM and Gupta G. DNA repeats in the human genome. Genetica. 1999;106 1-2:15-36. doi:10.1023/a:1003716509180.

18. Watson M and Warr A. Errors in long-read assemblies can critically affect protein prediction. Nat Biotechnol. 2019;37 2:124-6. doi:10.1038/s41587-018-0004-z.

19. Ye C, Hill CM, Wu S, Ruan J and Ma ZS. DBG2OLC: Efficient Assembly of Large Genomes Using Long Erroneous Reads of the Third Generation Sequencing Technologies. Sci Rep. 2016;6:31900. doi:10.1038/srep31900.

20. Boetzer M and Pirovano W. SSPACE-LongRead: scaffolding bacterial draft genomes using long read sequence information. BMC Bioinformatics. 2014;15:211. doi:10.1186/1471-2105-15-211.

21. Zimin AV, Marçais G, Puiu D, Roberts M, Salzberg SL and Yorke JA. The MaSuRCA genome assembler. Bioinformatics. 2013;29 21:2669-77. doi:10.1093/bioinformatics/btt476.

22. Luo J, Lyu M, Chen R, Zhang X, Luo H and Yan C. SLR: a scaffolding algorithm based on long reads and contig classification. BMC Bioinformatics. 2019;20 1:539. doi:10.1186/s12859-019-3114-9.

23. English AC, Richards S, Han Y, Wang M, Vee V, Qu J, et al. Mind the gap: upgrading genomes with Pacific Biosciences RS long-read sequencing technology. PLoS One. 2012;7 11:e47768. doi:10.1371/journal.pone.0047768.

24. Piro VC, Faoro H, Weiss VA, Steffens MB, Pedrosa FO, Souza EM, et al. FGAP: an

automated gap closing tool. BMC Res Notes. 2014;7:371. doi:10.1186/1756-0500-7-371.

25. McGinnis S and Madden TL. BLAST: at the core of a powerful and diverse set of sequence analysis tools. Nucleic Acids Res. 2004;32 Web Server issue:W20-5. doi:10.1093/nar/gkh435.

26. Kammonen JI, Smolander OP, Paulin L, Pereira PAB, Laine P, Koskinen P, et al. gapFinisher: A reliable gap filling pipeline for SSPACE-LongRead scaffolder output. PLoS One. 2019;14 9:e0216885. doi:10.1371/journal.pone.0216885.

27. Kosugi S, Hirakawa H and Tabata S. GMcloser: closing gaps in assemblies accurately with a likelihood-based selection of contig or long-read alignments. Bioinformatics. 2015;31 23:3733-41. doi:10.1093/bioinformatics/btv465.

28. Ramos RT, Carneiro AR, Caracciolo PH, Azevedo V, Schneider MP, Barh D, et al. Graphical contig analyzer for all sequencing platforms (G4ALL): a new stand-alone tool for finishing and draft generation of bacterial genomes. Bioinformation. 2013;9 11:599-604. doi:10.6026/97320630009599.

29. de Sa PH, Miranda F, Veras A, de Melo DM, Soares S, Pinheiro K, et al. GapBlaster-A Graphical Gap Filler for Prokaryote Genomes. PLoS One. 2016;11 5:e0155327. doi:10.1371/journal.pone.0155327.

30. Salmela L, Sahlin K, Makinen V and Tomescu AI. Gap Filling as Exact Path Length Problem. J Comput Biol. 2016;23 5:347-61. doi:10.1089/cmb.2015.0197.

31. Xu G-C, Xu T-J, Zhu R, Zhang Y, Li S-Q, Wang H-W, et al. LR_Gapcloser: a tiling path-based gap closer that uses long reads to complete genome assembly. GigaScience. 2018;8 1 doi:10.1093/gigascience/giy157.

32. Weirather JL, de Cesare M, Wang Y, Piazza P, Sebastiano V, Wang XJ, et al. Comprehensive

comparison of Pacific Biosciences and Oxford Nanopore Technologies and their applications to transcriptome analysis. F1000Res. 2017;6:100. doi:10.12688/f1000research.10571.2.

33. Wenger AM, Peluso P, Rowell WJ, Chang PC, Hall RJ, Concepcion GT, et al. Accurate circular consensus long-read sequencing improves variant detection and assembly of a human genome. Nat Biotechnol. 2019; doi:10.1038/s41587-019-0217-9.

34. Jain M, Koren S, Miga KH, Quick J, Rand AC, Sasani TA, et al. Nanopore sequencing and assembly of a human genome with ultra-long reads. Nat Biotechnol. 2018;36 4:338-45. doi:10.1038/nbt.4060.

35. Zook JM, Chapman B, Wang J, Mittelman D, Hofmann O, Hide W, et al. Integrating human sequence data sets provides a resource of benchmark SNP and indel genotype calls. Nat Biotechnol. 2014;32 3:246-51. doi:10.1038/nbt.2835.

36. Deng L, Guo L, Xu M, Wang W, Gu S, Zhao X, et al. SLR-superscaffolder: a de novo scaffolding tool for synthetic long reads using a top-to-bottom scheme. bioRxiv. 2019:762385. doi:10.1101/762385.

37. Weisenfeld NI, Kumar V, Shah P, Church DM and Jaffe DB. Direct determination of diploid genome sequences. Genome Res. 2017;27 5:757-67. doi:10.1101/gr.214874.116.

38. Guan R. Updated draft genome assembly of Ginkgo biloba. Gigascience Database. 2019; doi:https://doi.org/10.5524/100613.

39. Guan Rea. Updated draft genome assembly of Ginkgo biloba. Gigascience Database. 2019; doi:https://doi.org/10.5524/100613

40. Koren S, Walenz BP, Berlin K, Miller JR, Bergman NH and Phillippy AM. Canu: scalable and accurate long-read assembly via adaptive k-mer weighting and repeat separation. Genome

Res. 2017;27 5:722-36. doi:10.1101/gr.215087.116.

41. Li H. Minimap2: pairwise alignment for nucleotide sequences. Bioinformatics. 2018;34 18:3094-100. doi:10.1093/bioinformatics/bty191.

42. Walker BJ, Abeel T, Shea T, Priest M, Abouelliel A, Sakthikumar S, et al. Pilon: an integrated tool for comprehensive microbial variant detection and genome assembly improvement. PLoS One. 2014;9 11:e112963. doi:10.1371/journal.pone.0112963.

43. Vaser R, Sovic I, Nagarajan N and Sikic M. Fast and accurate de novo genome assembly from long uncorrected reads. Genome Res. 2017;27 5:737-46. doi:10.1101/gr.214270.116.

44. Gurevich A, Saveliev V, Vyahhi N and Tesler G. QUAST: quality assessment tool for genome assemblies. Bioinformatics. 2013;29 8:1072-5. doi:10.1093/bioinformatics/btt086.

45. Simao FA, Waterhouse RM, Ioannidis P, Kriventseva EV and Zdobnov EM. BUSCO: assessing genome assembly and annotation completeness with single-copy orthologs. Bioinformatics. 2015;31 19:3210-2. doi:10.1093/bioinformatics/btv351.

46. Brandt DY, Aguiar VR, Bitarello BD, Nunes K, Goudet J and Meyer D. Mapping Bias Overestimates Reference Allele Frequencies at the HLA Genes in the 1000 Genomes Project Phase I Data. G3 (Bethesda). 2015;5 5:931-41. doi:10.1534/g3.114.015784.

47. Guan R, Zhao Y, Zhang H, Fan G, Liu X, Zhou W, et al. Draft genome of the living fossil Ginkgo biloba. Gigascience. 2016;5 1:49. doi:10.1186/s13742-016-0154-1.

48. Teeling EC, Vernes SC, Davalos LM, Ray DA, Gilbert MTP and Myers E. Bat Biology, Genomes, and the Bat1K Project: To Generate Chromosome-Level Genomes for All Living Bat Species. Annu Rev Anim Biosci. 2018;6:23-46. doi:10.1146/annurev-animal-022516-022811.

Author notes

Mengyang Xu, Lidong Guo and Shengqiang Gu contributed equally to this work.

Table 2. Gap Filling accuracy statistics and computational consumptions for TGS-GapCloser.

Accuracy in long-read selection						
**Input data**	**No. of filled gaps**	**No. of filled gaps in theory**	**PPV (%)**	**Sensitivity (%)**	**Runtime (hours)**	**Peak memory (GB)**
MaSuRCA+SLR-superscaffolder+TGS-GapCloser (ONT)	75,629	74,353	96.6	96.3	259	50.07
MaSuRCA+SLR-superscaffolder+TGS-GapCloser (Pacbio)	74,321	74,353	98.2	89.8	13	32.97
Mercedes+SLR-superscaffolder+TGS-GapCloser (ONT)	58,938	61,267	97.7	93.4	145	50.52

Mercedes+SLR-superscaffolder+TGS-GapCloser (Pacbio)	52,116	61,267	98.4	75.6	11	31.74
Supernova+TGS-GapCloser (ONT)	22,563	24,760	62	51.2	163	73.61
Supernova+TGS-GapCloser (Pacbio)	26,919	24,760	76.1	61.2	20	38.47

Accuracy in single-base level

**Input data**	**No. of filled bases (bp)**	**No. of filled bases in theory (bp)**	**Input QV (Phred)**	**Output QV (Phred)**	**Filled QV (Phred)**
MaSuRCA+SLR-superscaffolder+TGS-GapCloser (ONT)	335,541,557	353,352,038	40.68	36.03	23.26
MaSuRCA+SLR-superscaffolder+TGS-GapCloser (Pacbio)	227,853,495	353,352,038	40.68	37.09	33.42
Mercedes+SLR-superscaffolder+TGS-GapCloser (ONT)	352,316,717	497,208,670	48.30	40.01	23.24

Mercedes+SLR-superscaffolder+TGS-GapCloser (Pacbio)	189,250,142	497,208,670	48.30	40.78	34.07
Supernova+TGS-GapCloser (ONT)	49,669,581	38,276,270	48.65	45.91	23.13
Supernova+TGS-GapCloser (Pacbio)	26,015,792	38,276,270	48.65	46.22	30.20

All datasets were run with 42 threads. Note that the peak memory consumption by Pilon or Racon is not counted. The higher speed of runs using the Pacbio HiFi dataset mainly originates from the usage of Racon to correct fragments with long reads.

Table 3. Enhancements in MHC region assembly by TGS-GapCloser.

	**MaSuRCA+SLR-superscaffolder+TGS-GapCloser**		**Mercedes+SLR-superscaffolder+TGS-GapCloser**		**Supernova+TGS-GapCloser**		**Ref.（33）**	
	draft	updated	draft	updated	draft	updated	rel3	rel5
No. of scaffolds (>1kb)	2	2	3	3	1	1	/	/

No. of contigs (>1kb)	339	31	271	26	76	12	7	1
Non-N bases (bp)	5,293,785	5,907,069	4,134,156	5,445,373	5,831,980	5,988,090	5,739,339	5,628,041
No. of gaps	343	31	268	23	81	16	/	/
Scaffold NG50 (bp)	3,400,000	3,400,000	4,400,000	4,400,000	6,000,000	6,000,000	/	/
Scaffold NGA50 (bp)	232,462	396,537	182,662	429,613	649,591	534,616	/	/
Contig NG50 (bp)	17,483	324,807	12,244	450,213	110,320	980,326	3,007,673	5,628,041
Contig NGA50 (bp)	16,630	199,405	11,901	321,624	94,556	380,102	49,485	52,555
Genome Fraction (%)	82.801	92.623	67.869	85.609	93.887	95.292	62.521	59.855
No. of misassemblies	11	25	13	22	15	17	20	53
No. of local misassemblies	34	101	11	122	29	42	546	484

The statistical results were generated by QUAST.

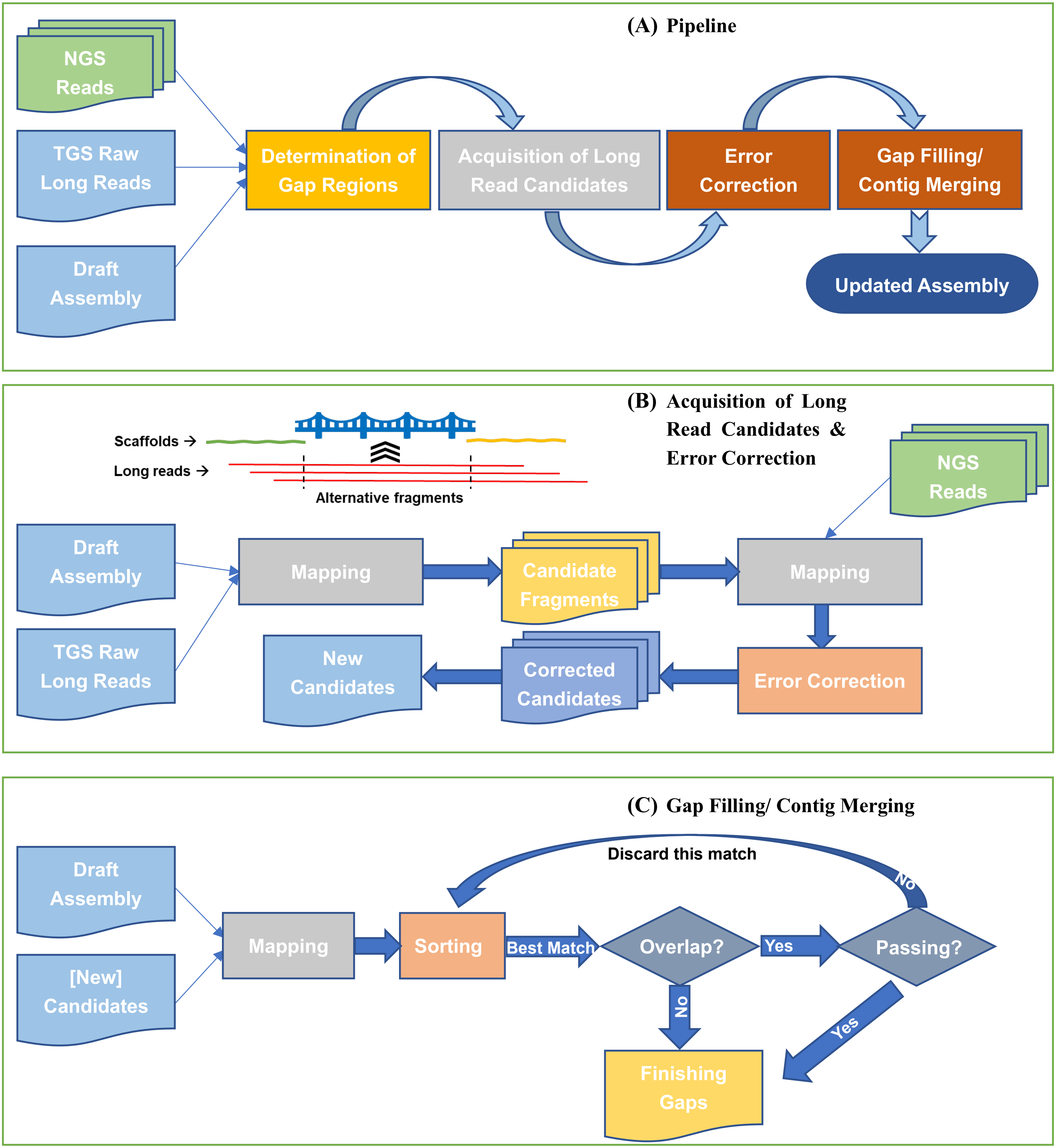
(A) Pipeline
NGS Reads
TGS Raw Long Reads
Draft Assembly
Determination of Gap Regions
Acquisition of Long Read Candidates
Error Correction
Gap Filling/ Contig Merging
Updated Assembly
(B) Acquisition of Long Read Candidates & Error Correction
Scaffolds →
Long reads →
Alternative fragments
NGS Reads
Draft Assembly
TGS Raw Long Reads
Mapping
Candidate Fragments
Mapping
Error Correction
Corrected Candidates
New Candidates
(C) Gap Filling/ Contig Merging
Discard this match
Draft Assembly
[New] Candidates
Mapping
Sorting
Best Match
Overlap?
Yes
Passing?
No
No
Yes
Finishing Gaps

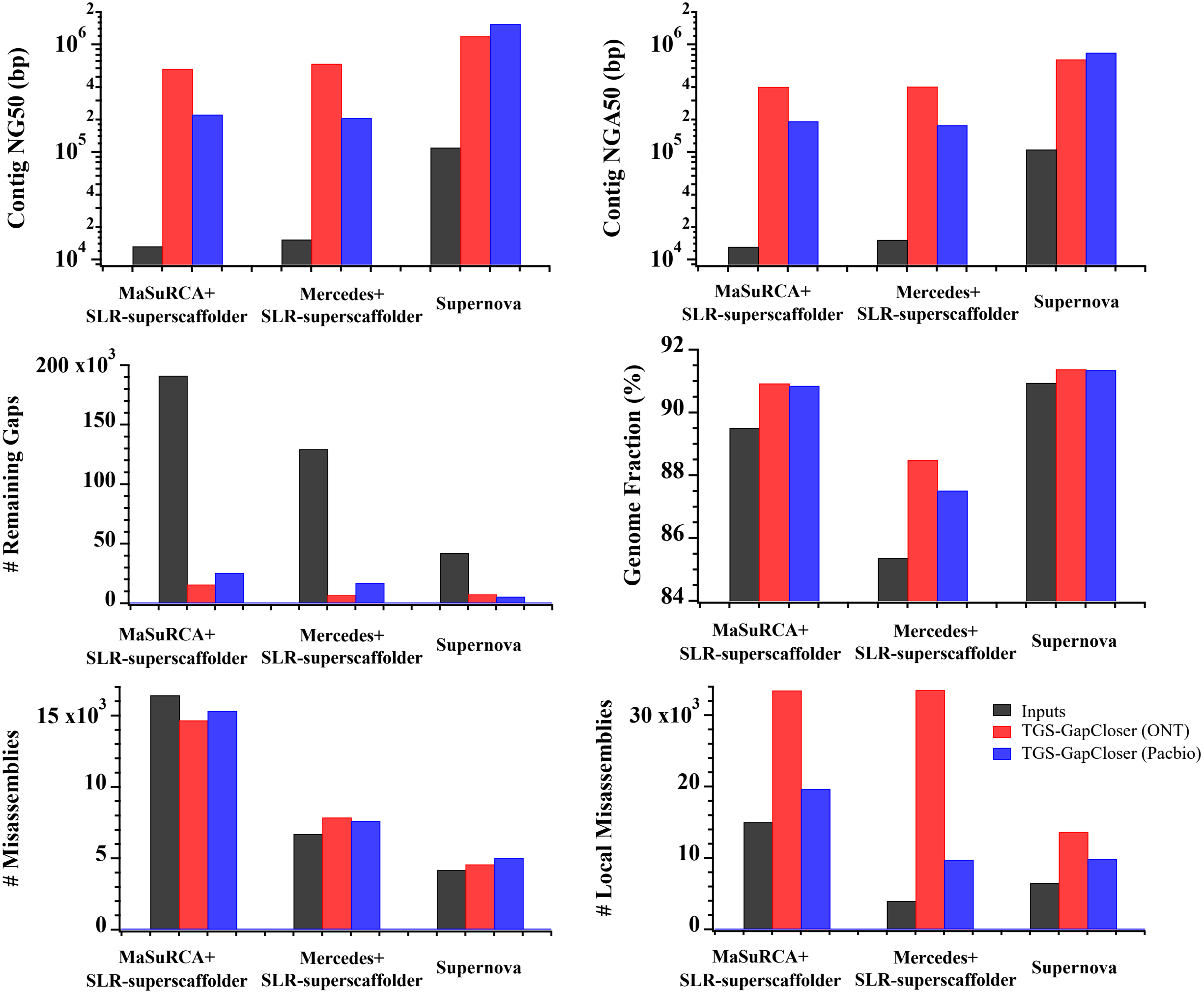

Contig NG50 (bp)
10^4
10^5
10^6
Contig NGA50 (bp)
Remaining Gaps
200 x10^3
150
100
50
0
Genome Fraction (%)
92
90
88
86
84
Misassemblies
15 x10^3
10
5
0
Local Misassemblies
30 x10^3
20
10
0
MaSuRCA+
SLR-superscaffolder
Mercedes+
SLR-superscaffolder
Supernova
Inputs
TGS-GapCloser (ONT)
TGS-GapCloser (Pacbio)

Click here to access/download
Supplementary Material
SI_TGS-GapCloser_GigaScience_version5.docx

Cover Letter
Click here to access/download;Personal Cover;cover_letter.docx

February 2nd, 2020

Dear *GigaScience* Editor,

It is our great pleasure to submit the enclosed manuscript for your consideration of publishing on *GigaScience*. The brief of our submission is:

Title: TGS-GapCloser: A fast and accurate gap closer for large genomes with low coverage of error-prone long reads

Authors: Mengyang Xu, Lidong Guo, Shengqiang Gu, Ou Wang, Rui Zhang, Guangyi Fan, Xun Xu, Li Deng & Xin Liu

Manuscript type: Technical Notes

The application of third-generation sequencing technology has brought a revolution in life and biomedical fields, but suffers the problem of expense and accuracy. We developed a gap-closing software tool, TGS-GapCloser that utilizes only low depth of single molecule sequencing long reads to discover the complicated areas in large genomes that short reads cannot reach. We demonstrate that TGS-GapCloser improves the continuity, completeness of human genome and gingko ultra large genome without loss of accuracy. Comparing with mainstream long-read gap-closing tools, it can complete more gaps in input assemblies, but run incredibly faster. We believe that the TGS-GapCloser-based hybrid assembly strategy comprehensively employs assembly information to the utmost extent from various sequencing platforms, and improves the quality of downstream analysis of gene annotation. The low-depth requirement of expensive long reads makes this approach more costly effective and suitable for the community with small budgets, and readily enlarges the “big data” database.

All authors have declared that they have no competing interests, approved the contents of the manuscript and agreed with the submission to *GigaScience*. This manuscript is not under consideration for publication elsewhere and has been preprinted in bioRxiv only. We look forward to hearing from you soon. Your kind assistance on this is greatly appreciated!

Sincerely yours,

Mengyang Xu, Ph.D.

BGI-Research

BGI-Qingdao, BGI-SZ, Qingdao 266555, China
