# Supplementary material for: TGS-GapCloser: A fast and accurate gap closer for large genomes with low coverage of error-prone long reads
Source: Gigascience. 2020 Sep 7;9(9):giaa094. doi: 10.1093/gigascience/giaa094 (PMC7476103; doi:10.1093/gigascience/giaa094)
Supplement: giaa094_GIGA-D-20-00014_Revision_1 [file giaa094_giga-d-20-00014_revision_1.pdf]

GigaScience

TGS-GapCloser: A fast and accurate gap closer for large genomes with low coverage of error-prone long reads.

--Manuscript Draft--

**Manuscript Number:**	GIGA-D-20-00014R1
**Full Title:**	TGS-GapCloser: A fast and accurate gap closer for large genomes with low coverage of error-prone long reads.
**Article Type:**	Technical Note
**Funding Information:**	National Key Research and Development Program of China (2018YFD0900301-05) — Dr. Guangyi Fan<br>Qingdao Applied Basic Research Projects (19-6-2-33-cg) — Dr. Mengyang Xu
**Abstract:**	Background: The continuity, completeness and accuracy of genome assemblies determine the quality of subsequent bioinformatics analysis. Despite benefiting from the medium/long-range information, the employment of single molecule sequencing techniques to enhance assemblies suffers from the substantial sequencing cost and computational consumption, especially for large genomes (>1Gb).<br>Findings: We developed a gap-closing tool, TGS-GapCloser that uses low-depth (~10×) long reads to close gaps for large genomes. The algorithm extracts long-read gap regions from the alignments against input scaffolds, corrects only the candidate fragments, and assigns the best sequences to each gap. We demonstrate that TGS-GapCloser improves the scaftig NG50 value of three human genome assemblies by 24-fold on average with only ~10× coverage of ONT or Pacbio reads, completing up to 94.8% gaps with 97.7% positive predictive value. Despite of high error rate of raw long reads, the improved assembly achieves 99.998% (Q46) single-base accuracy with final inserted sequences of 99.97% (Q35) accuracy. This promises the high-quality downstream analysis, including that up to 31-fold increment in the scaftig NGA50 and up to 13.1% more BUSCO genes are completed. It also shows the power to improve the ultra large genome assembly of ginkgo (~12Gb) with 71.6% of gaps closed. The validation of inserted sequences was conducted with reference genomes and GIAB benchmark sets.<br>Conclusions: TGS-GapCloser can close gaps in large genome assemblies using raw long reads in a fast and cost-effective way, and improve the continuity, completeness without loss of accuracy. The software is available at https://github.com/BGI-Qingdao/TGS-GapCloser .
**Corresponding Author:**	Mengyang Xu<br>BGI<br>CHINA
**Corresponding Author Secondary Information:**	
**Corresponding Author's Institution:**	BGI
**Corresponding Author's Secondary Institution:**	
**First Author:**	Mengyang Xu
**First Author Secondary Information:**	
**Order of Authors:**	Mengyang Xu<br>Lidong Guo<br>Shengqiang Gu<br>Ou Wang<br>Rui Zhang<br>Guangyi Fan

Powered by Editorial Manager® and ProduXion Manager® from Aries Systems Corporation

	Xun Xu
	Li Deng
	Xin Liu
**Order of Authors Secondary Information:**	
**Response to Reviewers:**	Dear Editor,<br>Thank you for giving us the opportunity to submit a revised draft of the manuscript entitled “TGS-GapCloser: A fast and accurate gap closer for large genomes with low coverage of error-prone long reads.” (GIGA-D-20-00014) to GigaScience. We appreciate the time and effort that you and the reviewers have dedicated to providing your valuable feedback on the manuscript. We are grateful to the reviewers for their insightful comments, and have incorporated changes to reflect most of the suggestions. Here is a point-by-point response to the reviewers’ comments and concerns.<br><br>Response to Reviewer #1:<br>General Comment: The authors present a new method for gap closing with low coverage raw long reads. Experiments on draft genomes with contigs assembled from short reads and scaffolds from long range reads show the tool works well, and more efficiently compared to other tools. The manuscript overall is written and organized well. But I have the following concerns:<br><br>Response: We appreciate the reviewer’s positive feedback about the efficiency of the software tool, and the organization and quality of the manuscript.<br><br><br>Comment 1: With more long reads sequenced, more genomes are directly assembled from long reads and then scaffolded or phased together with HiC and/or linked reads. Even though the cost is higher than short reads, different from applications like SV calling, genome assembly is done once and most of the cases the cost could be tolerated. The target application of the proposed method is using low coverage long reads to fill the gaps on draft genome assembled from short reads. The authors need to clearly define how "low" coverage they could perform better than with direct assembly from long reads. The authors show the performance of their tool on different coverage of long reads, but only on the draft genome assembled from short reads. How about the genome directly assembled from long reads? Say at 20X? If it is already good enough, then no need for gap closing at this coverage. This is pretty important as it defines the potential roles the proposed method could play.<br><br>Response: Thank you for raising this important point here. The relatively high cost of de novo assembly from long reads is from both expensive sequencing and high computing consumption, in spite of much effort to decrease the sequencing price and simplify the computation. A recent discussion of the effect of sequence depth and length in long-read assembly (Ou S, et al, Nature Communications. 2020;11 1:2288. doi:10.1038/s41467-020-16037-7) suggest that >30× sequencing coverage and >11kb N50 read length are required to obtain a high-quality assembly.<br>Actually, we extracted 1×, 5×, 10×, and 20× sequencing depth (coverage) of ONT reads for the human chromosome 19 and separately assembled them by Canu. The results indicate that those long reads cannot be directly used to assemble the whole chromosome (contig N50~40kb, and reference genome coverage ~30.7% with 20x sequencing depth) although the assembly is sharply improved with increasing depth. In contrast, TGS-GapCloser exhibits obvious assembly improvements (scaftig N50 ~454kb, and reference genome coverage~98.2%with only ~10× sequencing depth) on the basis of stLFR assembly (scaftig N50 ~27kb, genome coverage~97.9%). The comparison was summarized in Table S5.<br>In this study, we expect that the utilization of TGS long reads acts as a significant part in a hybrid assembly pipeline, which benefits from advantages of different sequencing techniques instead of TGS alone.<br><br><br>Comment 2: The major advantages in speed and memory cost over other tools come from using other third party tools that perform good, like minimap2, while the compared tools use slow tools like "blast". It's not from method innovation or better algorithm

Powered by Editorial Manager® and ProduXion Manager® from Aries Systems Corporation

design, although selecting proper tools is also important.

Response: We agree that minimap2 is faster than BLAST or BLASR, and the choice of the aligner plays an important role in the performance. In our work, we also tried to speed up the gap-closing process through specific designs such as: 1. Fragmenting long reads as candidates and limiting the number of candidates corresponding to each gap for correction and competition to reduce the computing amount (30Gb long reads for human decreased to 1.9Gb candidates), and 2. A concise but efficient scoring system of candidates to reduce the computational complexity (Table S3).
In our tests, TGS-GapCloser is less time-consuming than BLASR-based PBJelly, BLAST-based FGAP, and the newly added minmap2-based Cobbler as suggested by Reviewer #2 (Table 2). It is difficult to owe the speed to the aligner only because their algorithm designs and best applicable scopes are different. The comparison demonstrates that TGS-GapCloser focuses more on the accuracy of the long-read candidate selection. This discussion was added to the section “Algorithm and implementation of TGS-GapCloser”, current version, page 12, line 2-9.

Comment 3: HG001 has HiFi long reads released by GIAB from last year (2019). The authors may consider switching to it.

Response: As suggested by the reviewer, we replaced the Pacbio HiFi reads of HG002 with that of HG001/NA12878 obtained from GIAB, and updated the gap-closing results in the manuscript. The improvements of the genome assembly after gap closure are comparable. On average, there are 2% increase in scaftig NG50, and 3% increase in scaftig NGA50 after the replacement for the three human assemblies. The effect on BUSCO results is almost ignorable.

Response to Reviewer #2:
Comment 1: The authors present a novel gap-filling algorithm that utilizes low coverage long read data, useful for augmenting existing assemblies. The algorithm appears sound and would be very useful for the bioinformatics community and experimental design and measurements/evaluations, for the most part, seem well designed. However, some more evaluations and comparisons to more tools may be needed. Also, the manuscript has some issues with the quality of the writing.
The writing of the manuscript needs work. Interestingly, it appears that attempts at correction were done but the manuscript seems like it had been proofread and edited by someone who didn't understand the content or just run through grammar checking software without consideration of the content, resulting in a manuscript that was somewhat difficult to read and inaccurate in some places. Giving the authors the benefit of the doubt it is possible to piece together the reasonable intent within the writing, but this takes a great deal of effort and guesswork.
For example, the problems with writing the paper start at the very beginning in the background of the abstract which states that "gap-closing tools suffer multi-alignments and high error rates". The authors likely intend for it to read something like "the long reads suffer from high error rates which reduce the performance of current long read based gap closing tools". Following this statement, they claim that this results in a "huge time and money costs" but these issues are not logically a direct result of the previous point. Perhaps the authors intended to state something like "due to the poor performance of these tools, high long read coverage is needed resulting in huge time and monetary costs". This kind of issue is just the tip of the iceberg of problems related to the overall writing of the paper and the authors need to be more careful in their writing in general to make this manuscript of publishable quality.

Response: We appreciate the positive feedback from the reviewer about the algorithm and the comments about the writing quality. We have carefully investigated the confused part mentioned above and gone through the whole manuscript, and realized that the reason is because we tried to express our opinion but extend to other points of view with few words. Thus, we split the sentence to make it more logically sound, and only focus on the closely related points. The main changes have been made as follows:
1)Abstract: Background, original version, page 2, line 3-6: “Despite benefiting from the

Powered by Editorial Manager® and ProduXion Manager® from Aries Systems Corporation

	medium/long-range information of single molecule sequencing techniques, current gap-closing tools to enhance assemblies suffer multi-alignments and high error rates, resulting in huge time and money costs, especially for large genomes." To "Despite benefiting from the medium/long-range information, the employment of single molecule sequencing techniques to enhance assemblies suffers from the substantial sequencing cost and computational consumption, especially for large genomes (>1Gb)."<br>2)Findings: Introduction, original version, page 3, line 16-18: "However, all the finished assemblies are imperfect, even for human and model organisms, which contain gaps of unknown nucleic acids (represented by Ns)." To "However, the finished assemblies for human or other large organisms remain imperfect, which contain gaps of unknown nucleic acids (represented by N's)[1, 2]. "<br>3)Findings: Introduction, original version, page 4, line 4-6: "But the manual or semi-automated processes limit the applications in consideration of huge costs." To "But the sequencing and labor costs hindered the manual or semi-automated gap-closing processes[1]."<br>4)Findings: Introduction, original version, page 5, line 11-17: "However, most tools mentioned above share the same crucial shortcoming: they function well only with pre-error-corrected or simulated long reads. It hampers the application because the error correction needs sufficient coverage of expensive long reads or extra short reads, and requires huge time and memory consumption, but usually splits long reads into short fragments and loses valuable assembly length information, not readily usable for large genomes." To "These tools have been widely used to close gaps with TGS long reads, but their efficiencies and accuracies are substantially dependent on the quality of input long reads. PBJelly improves the quality of inserted long reads through local assembly, but requires sufficient coverage. Other tools bypass the problem of input quality, and require or recommend pre-error corrected long reads or pre-assembled contigs. However, the additional assembly or correction for all the reads prior to gap closure needs adequate coverage of expensive long reads or extra short reads, and requires huge time and memory consumption, especially for large genomes. In addition, the correction algorithms might trim ambiguous segments[3] and split long reads into short fragments[4] due to the undetermined bases, thus losing valuable length information."<br>5)Findings: Introduction, original version, page 6, line 5-6: "High error rate and the existence of repeats may increase the probability of large misassembly events." To "The misalignments of long reads against the scaffolds owing to base-calling errors or repeats might increase the probability of large misassembly events. An effective scoring mechanism prevents the gap-closing tools from making wrong choice of filled fragments to some extent."<br>6)Findings: Algorithm and implementation of TGS-GapCloser, original version, page 9, line 20-22: "The alignment amount and quality determine the efficiency and accuracy of gap closure. Thus, all alignments were filtered based on the alignment length and identity ratio." To "The quantity and quality of candidates determine the efficiency and accuracy of gap closure. Thus, we designed a scoring system of candidates for quality control and filtration based on the length and identity ratio (matched bases/ aligned bases) of the alignment between a long-read candidate and flanking scaftig ends in the gap."<br>7)Findings: Algorithm and implementation of TGS-GapCloser, original version, page 10, line 21-22: "…a candidate with higher-quality alignments could be mapped to a more precise position in the reference…" to "…the QS of a candidate with higher-quality alignments would be increased due to the more precise mapping to the gap after error correction …"<br><br>Comment 2: The mention of the correctness of the assembly (q46) in the manuscript is somewhat meaningless unless compared to the quality before the tool was run. It might also make more sense if the authors state the correctness of only the filled regions, as this quality metric is likely washed out by the overall assembly quality.<br><br>Response: We agree that the single-base accuracy of the inserted sequences is washed out by that of the overall assembly because of the small ratio. We have, accordingly, added the quality of the inserted sequences to the abstract and emphasized the changes in both inserted and overall quality after the gap closure in Table 1. Although the corrected long-read fragments decrease the overall quality, they are improved from Q17 for raw to Q29 at last on average. This is a result of the accurate selection of long read to close the gap and the single-base level error

correction.
As we noted in our response to Reviewer #1, TGS-GapCloser aims at improving the assembly quality as an important part of the comprehensive utilization of advantages from different techniques. The change in the final overall correctness induced by inserted sequences has a great influence on the quality of follow-up bioinformatics analyses. Thus, we retain the description of the overall correctness in the abstract but mark the contribution from the inserted sequences as suggested by the reviewer.

Comment 3: Contig NG50 was specified in the abstract but gap-filling typically is performed on scaffolds and one would not expect NG50 to increase substantially after gap-filling. Did the authors mean scaftig NG50? Better yet, perhaps scaftig NGA50 should be stated in the abstract instead. I noticed scaftigs seemed to be mentioned in the manuscript equating them to contigs; to prevent confusion consider replacing all instances of contig with scaftig.

Response: Thank you for pointing out the difference between contig and scaftig. As suggested by the reviewer, the “scaftig” is used to replace “contig”, and refers to the continuous sequence within a scaffold without N’s in the current main text.

Comment 4: It seems strange to me that various tools like LR-Gapcloser were not even benchmarked and merely stated that they "did not show any obvious improvements in efficiency and accuracy". Indeed, if PB-Jelly published in 2012 was compared, why were these other tools omitted? Other tools omitted from comparisons are Cobbler (Warren et al. 2016), GMcloser (Kosugi et al. 2015) and others may exist all of which should be considered in the comparisons unless a compelling reason they did not can be convincingly stated.
If the tools cannot be run due to resource limitations, authors should at least make this clear by benchmarking on smaller datasets and show how resource usage scales or at the very least state the algorithm does not run given their resources (explaining possible reasons why it does not scale). Unless the authors can definitively prove that these tools are not worth benchmarking as they have already shown performance less than the current state-of-the-art tools (to which TGS-GapCloser is compared to) in other publications in almost all dimensions, it doesn't make sense to me that these tools should be omitted from the comparisons.

Response: As suggested by the reviewer, we added three latest tools to the comparison, including GMcloser, Cobbler and LR_Gapcloser. Since PBJelly and FGAP could not close gaps for the whole human genome, we uniformly applied them to the assembly of the human chromosome 19. The results are expressed in the section “Comparison with other gap-closing tools” and listed in Table 2. The BLAST-based GMcloser and FGAP, and the BLASR-based PBJelly consume much more time than others. Newer tools such as Cobbler and LR_Gapcloser obviously improve the speed and memory consumption relative to prior tools, but they are still slower than TGS-GapCloser. More importantly, the gap-closing accuracy of these tools in terms of scaftig NGA50 (14% of TGS-GapCloser’s on average) and introduced assembly errors (2.4-fold more than TGS-GapCloser on average) are clearly worse than that of TGS-GapCloser, although the number of closed gaps and the improved scaftig continuity are close. Their algorithms need to be adjusted to accept the error-prone input, increase the accuracy in the selection of inserted sequences, and improve the gap-closing efficiency for low coverage of raw long reads.

Comment 5: Installation was very easy but might be helpful to add the tool to conda and linuxbrew but this is a very minor point as there are very few dependencies.

Response: As suggested by the reviewer, we have uploaded the software tool to conda, and are considering to add it to linuxbrew.

1.English AC, Richards S, Han Y, Wang M, Vee V, Qu J, et al. Mind the gap: upgrading genomes with Pacific Biosciences RS long-read sequencing technology. PLoS One.

Powered by Editorial Manager® and ProduXion Manager® from Aries Systems Corporation

	2012;7 11:e47768. doi:10.1371/journal.pone.0047768.<br>2.Schneider VA, Graves-Lindsay T, Howe K, Bouk N, Chen HC, Kitts PA, et al. Evaluation of GRCh38 and de novo haploid genome assemblies demonstrates the enduring quality of the reference assembly. Genome Res. 2017;27 5:849-64. doi:10.1101/gr.213611.116.<br>3.Koren S, Walenz BP, Berlin K, Miller JR, Bergman NH and Phillippy AM. Canu: scalable and accurate long-read assembly via adaptive k-mer weighting and repeat separation. Genome Res. 2017;27 5:722-36. doi:10.1101/gr.215087.116.<br>4.Walker BJ, Abeel T, Shea T, Priest M, Abouelliel A, Sakthikumar S, et al. Pilon: an integrated tool for comprehensive microbial variant detection and genome assembly improvement. PLoS One. 2014;9 11:e112963. doi:10.1371/journal.pone.0112963.<br><br><br>Additional clarifications:<br>In addition to the above responses, we also made two changes:<br>1.We added one citation (Murigneux V., et al. Comparison of long read methods for sequencing and assembly of a plant genome. bioRxiv. 2020:2020.03.16.992933. doi: https://doi.org/10.1101/2020.03.16.992933) as an extra support that TGS-GapCloser leads to an obvious increase in the scaftig N50 with BUSCO detecting more complete genes.<br>2.In the QUAST evaluations, we changed the human reference assembly from GRCh38.p13 to hs37d5, which excludes ambiguous sequences such as ALT, unplaced and unlocalized sequences to avoid false misassemblies aroused by the alignments between assemblies and those ambiguous segments.<br>Meanwhile, all spelling and grammatical errors have been checked and corrected.<br>We look forward to hearing from you in due time regarding our submission and to respond to any further questions and comments you or reviewers may have.<br><br>Sincerely,<br>Mengyang Xu
Additional Information:	
Question	Response
Are you submitting this manuscript to a special series or article collection?	No
**Experimental design and statistics**<br><br><br>Full details of the experimental design and statistical methods used should be given in the Methods section, as detailed in our Minimum Standards Reporting Checklist. Information essential to interpreting the data presented should be made available in the figure legends.<br><br><br>Have you included all the information requested in your manuscript?	Yes
**Resources**<br><br><br>A description of all resources used, including antibodies, cell lines, animals and software tools, with enough information to allow them to be uniquely	Yes

identified, should be included in the Methods section. Authors are strongly encouraged to cite Research Resource Identifiers (RRIDs) for antibodies, model organisms and tools, where possible.<br><br>Have you included the information requested as detailed in our Minimum Standards Reporting Checklist?	
**Availability of data and materials**<br><br>All datasets and code on which the conclusions of the paper rely must be either included in your submission or deposited in publicly available repositories (where available and ethically appropriate), referencing such data using a unique identifier in the references and in the "Availability of Data and Materials" section of your manuscript.<br><br>Have you have met the above requirement as detailed in our Minimum Standards Reporting Checklist?	Yes

Manuscript

Click here to access/download;Manuscript;TGS-GapCloser_GigaScience_version9.docx

TGS-GapCloser: A fast and accurate gap closer for large genomes with low coverage of error-prone long reads

Mengyang Xu[1,2,4,#], Lidong Guo[3,1,#], Shengqiang Gu[3,1,#], Ou Wang[4,6], Rui Zhang[1], Guangyi Fan[1,4], Xun Xu[4,5], Li Deng[1,2,4, *] & Xin Liu[1,2,4,5, *]

[1]BGI-Qingdao, BGI-Shenzhen, Qingdao 266555, China

[2]State Key Laboratory of Agricultural Genomics, BGI-Shenzhen, Shenzhen 518083, China

[3]BGI Education Center, University of Chinese Academy of Sciences, Shenzhen 518083, China

[4]BGI-Shenzhen, Shenzhen 518083, China

[5]China National GeneBank, BGI-Shenzhen, Shenzhen 518120, China

[6]MGI, BGI-Shenzhen, Shenzhen 518083, China

[#]These authors contributed equally to this work.

*Corresponding authors: Li Deng (dengli1@genomics.cn) and Xin Liu (liuxin@genomics.cn)

Abstract

Background: The continuity, completeness and accuracy of genome assemblies determine the quality of subsequent bioinformatics analysis. Despite benefiting from the medium/long-range information, the employment of single molecule sequencing techniques to enhance assemblies suffers from the substantial sequencing cost and computational consumption, especially for large genomes (>1Gb).

Findings: We developed a gap-closing tool, TGS-GapCloser that uses low-depth (~10×) long reads to close gaps for large genomes. The algorithm extracts long-read gap regions from the alignments against input scaffolds, corrects only the candidate fragments, and assigns the best sequences to each gap. We demonstrate that TGS-GapCloser improves the scaftig NG50 value of three human genome assemblies by 24-fold on average with only ~10× coverage of ONT or Pacbio reads, completing up to 94.8% gaps with 97.7% positive predictive value. Despite of high error rate of raw long reads, the improved assembly achieves 99.998% (Q46) single-base accuracy with final inserted sequences of 99.97% (Q35) accuracy. This promises the high-quality downstream analysis, including that up to 31-fold increment in the scaftig NGA50 and up to 13.1% more BUSCO genes are completed. It also shows the power to improve the ultra large genome assembly of ginkgo (~12Gb) with 71.6% of gaps closed. The validation of inserted sequences was conducted with reference genomes and GIAB benchmark sets.

Conclusions: TGS-GapCloser can close gaps in large genome assemblies using raw long reads in a fast and cost-effective way, and improve the continuity, completeness

without loss of accuracy. The software is available at https://github.com/BGI-Qingdao/TGS-GapCloser.

Keywords: gap-closure, third-generation sequencing, genome assembly, ginkgo, MHC

Findings

Introduction

The development of genome sequencing techniques has been reducing the cost and improving the throughput at a speed beyond the Moore's Law over the last decade[1]. The genetic sequence databases have been drastically enriched, and progressively increasing focuses move from small bacterial and fungal genomes to large eukaryotes. The application of state-of-the-art techniques, for instance, TGS long reads[2, 3], SLR libraries[4-8], Hi-C[9], and BioNano physical map[10], provides extra genomic information on different length scales to increase negentropy of the system, promising the enhanced genome assembly relative to the NGS-based assembly. However, the finished assemblies for human or other large organisms remain imperfect, which contain gaps of unknown nucleic acids (represented by N’s)[11-13]. The repetitiveness and polymorphism of the genomes, the limitation of sequencing platforms, and the trade-off of algorithms may lead to the gaps. Gap closure or gap filling can recover the unknown bases and extend scaftigs (contigs within a scaffold without N’s)[14] to entirely or partially missing gene-encoding area to bridge the

gaps. Therefore, there has been a need to develop tools to close gaps in the existing assemblies for more complete genomes, especially for large eukaryotic genomes with high complexity.

The first effort to finish gaps in draft genome assemblies was made using Fosmids, BACs libraries and Sanger reads[15]. But the sequencing and labor costs hindered the manual or semi-automated gap-closing processes[12]. The NGS technologies along with paired-end and mate-pair information of multiple insert sizes overcame the financial problem, and several landmark tools were designed to reach into gap regions [16-20], sharing similar *k*-mer-extension or local reassembly algorithms, but suffering the same problem of large CPU and memory consuming for large genomes. Besides, those strategies hardly spanned the repetitive DNA fragments such as tandem repeats and tended to cause more misassemblies due to the short read/*k*-mer length.

The current single molecule TGS technologies, including Pacbio and ONT, have the potential to break through these limitations as their reads (~10kb) are typically longer than most DNA repeats[21]. Although the *de novo* genome assembly using long reads alone may allow remarkable improvements, the lower accuracy relative to NGS platforms generally requires sufficient sequencing coverage and intense computational costs for error correction prior to or after the assembly[22]. The correction is essential as these base-calling errors may cause frameshifts in the gene-coding region, and thus disrupting the protein prediction[23].

There have been several hybrid assemblers designed to combine advantages of both sequencing platforms since the TGS commercial techniques were released. Main

strategies to utilize the medium/long-range information include constructing a final assembly graph by mixing NGS contigs and TGS long reads based on the OLC or string graph algorithm[24], and scaffolding the contigs generated by NGS dependent on their alignments against long reads[25-27]. In contrast, the gap-closing algorithm provides a direct way to reduce the computing complexity and costs, which only upgrades the missing regions and reserves the majority of the existing assembly information. PBJelly[12] is the first tool to use Pacbio reads to close gaps through locally assembling the long reads falling into gap regions. FGAP[28] selects the best matched pre-assembled contig to fill gaps based on the BLAST[29] alignments. GMcloser[30] tries to increase the accuracy of gap closure using likelihood-based classifiers. Cobbler[31] accepts new aligners to accelerate the buildup of the relation between long high-quality sequences (usually scaftigs/contigs from other assemblies) and input scaffolds, and patches the gaps if the long sequence meets the requirement of anchoring bases and sequence identity. LR_Gapcloser[32] shrinks the computational costs of alignments by fragmenting long reads into tags and aligning the short tags against scaffolds instead of the whole long reads. These tools have been widely used to close gaps with TGS long reads, but their efficiencies and accuracies are substantially dependent on the quality of input long reads. PBJelly improves the quality of inserted long reads through local assembly, but requires sufficient coverage. Other tools bypass the problem of input quality, and require or recommend pre-error corrected long reads or pre-assembled contigs. However, the additional assembly or correction for all the reads prior to gap closure needs adequate coverage of expensive

long reads or extra short reads, and requires huge time and memory consumption, especially for large genomes. In addition, the correction algorithms might trim ambiguous segments[33] and split long reads into short fragments[34] due to the undetermined bases, thus losing valuable length information.

It is necessary to comprehensively utilize assembly information in short range (NGS, <1kb), medium range (TGS, 1k~10kb) and long range (TGS, SLR, Hi-C, BioNano, 10kb~Mb) with different resolutions, and meanwhile balance their costs. Three key points should be considered to develop a TGS gap-closing algorithm. First, use TGS data as few as possible. Although the price has been decreasing[35], the gap-closing efficiency is still the first priority, particularly for those small labs or small projects. Thus, local reassembly or pre-error correction based on the long-read overlaps is not preferable. Another important factor is the accuracy and precision in the selection of long reads to fill the gaps. It has been demonstrated that the number of assembly errors caused by gap-closing tools is higher than that of *de novo* assembled scaftigs[30]. The misalignments of long reads against the scaffolds owing to base-calling errors or repeats might increase the probability of large misassembly events. An effective scoring mechanism prevents the gap-closing tools from making wrong choice of filled fragments to some extent. Last but not least, the filled sequences should not diminish the single-base level accuracy of the whole assembly, which influences the quality of downstream analysis. There is still a need of error correction or polish for the inserted raw long-read segments. Note that the most recent Pacbio improved its base-calling accuracy to 99.8%[36], which may directly simplify the

problem, although it sacrifices the throughout and read length.

In this work, we describe a software tool, named TGS-GapCloser, that straightforwardly uses error-prone long reads at low coverage to close gaps for large genomes in a more efficient and accurate way. We applied it to three *de novo* human genome assemblies with 10× coverage of ONT or Pacbio long reads[37, 38], and improved the scaftig NG50 11.0 to 45.0-fold and scaftig NGA50 6.8 to 30.6-fold dependent on different inputs. Besides, 71.6% gaps in the ultra large genome assembly of ginkgo are also closed using 10.5× coverage of corrected Pacbio reads, increasing the scaftig N50 from 57.1kb to 364.8kb. In addition, a recent study of the plant genome, *Macadamia janseni*, exhibits that TGS-GapCloser leads to a 29-fold increase in the scaftig N50 with BUSCO detecting 4.8% more complete genes[39]. The hybrid strategy of updating the draft *de novo* genome assembly with TGS-GapCloser might promote the quality of gene annotation and structure variation detection, thus improving the quality of downstream analysis of ontogeny, phylogeny, and evolution.

Data description

Three datasets of two large genomes were used to examine the gap-closing results by TGS-GapCloser: human, human chr19, and ginkgo. We sequenced *Homo sapiens* (HG001/NA12878) using MGIEasy stLFR Library Prep Kit on the BGISEQ-500 platform with the total data size of 660 Gb. Reads mapped to the Chr19 reference were also extracted for comparisons and further analysis. The short reads were

assembled using MaSuRCA (version 3.3.1)[26] or Mercedes (in-house tool) to obtain short but highly accurate contigs, and the SLR long-range (barcode/read cloud) and short-range (paired-end) information provided by stLFR technique was exploited to do further scaffolding by SLR-superscaffolder (version 1.0.0)[40]. In addition, Supernova (version 2.1.1)[41] was also used to obtain draft scaffolds although it was originally designed to assemble 10X Genomics data. To test the generalization and potential of TGS-GapCloser's application, we utilized new generation data of both long-read platforms to close gaps in each human genome assembly: ONT MinION Rel3 dataset (Rel3)[37] and Pacbio CCS HiFi dataset (HiFi)[38].

The input assembly of *Ginkgo biloba* female (estimated about 12 Gb) was obtained from [42], which was initially assembled using SOAPdenovo2[16] and updated using Hi-C data[43]. The Pacbio reads for ginkgo were sequenced by Pacbio Sequel, with the chemistry of Sequel Sequencing Kit 3.0 Bundle (4 rxn). The total data amount was 256 Gb with the average read length of 38,623 bp. Error correction by Canu[33] reduced the data size to 126 Gb, with the average read length of 10,722 bp. The basic statistics for input assemblies are listed in Table S1, and sequencing reads are in Table S4.

Algorithm and implementation of TGS-GapCloser

TGS-GapCloser accepts any type of TGS long reads or other pre-assembled contigs to automatically fill gaps in any type of draft assembly in the following four steps as shown in Figure 1: (i) determination of gap regions in the draft assembly; (ii)

acquisition of candidates from the alignments of long reads against gaps; (iii) base-level error correction of alternative sub-long reads; and (iv) gap closure using the error-corrected candidates with the highest score for each gap or linkage of the neighboring scaftigs with overlaps.

Figure 1. A schematic of TGS-GapCloser workflow. (A) A flow chart of the overall algorithm, (B) a schematic description for the determination of gap regions, the acquisition of candidate long-read fragments, and the error correction of alternative sub-long reads, (C) a detailed flow chart for gap filling or scaftig merging in a gap region with the most proper medium/long-range information provided by long reads.

The input scaffolds were firstly split into parts called scaftigs from the observed N positions, and each pair of neighboring scaftigs according to their positions in the shared scaffold were defined as a gap waiting to be filled. TGS-GapCloser defaults the high quality of input scaffolds, including the base-level accuracy, the order and orientation of scaftigs, but not the estimated gap size. The reason is because the long-range information provided by SLR, Hi-C, or BioNano barely reach a low resolution (<10kb), and the size estimations based on the information have a high probability of faults especially for small gaps.

We used minimap2[44] to align long reads against each gap to obtain the corresponding candidate fragments. A candidate for a specific gap is defined as the segment truncated from the aligned long reads in the N area between two neighboring

scaftigs plus 2kb-long flanking wings on both sides. Each long read might provide several candidate sequences dependent on its spanning length and base-calling accuracy, but was limited to give at most one candidate for the same gap to resolve the problem of redundant alignments induced by the alignment algorithm and long-read high error rate.

The quantity and quality of candidates determine the efficiency and accuracy of gap closure. Thus, we designed a scoring system of candidates for quality control and filtration based on the length and identity ratio (matched bases/ aligned bases) of the alignment between a long-read candidate and flanking scaftig ends in the gap. The score *QS* is given by

$$QS = a \cdot logA_i + b \cdot logI_i + a \cdot logA_{i+1} + b \cdot logI_{i+1}$$

where letter *A* refers to the alignment length, letter *I* refers to the identity ratio for the *i*th and *i+1*th scaftigs, respectively; letter *a* and *b* are two arbitrary coefficients to distinguish *A* and *I*'s weights on the score, and have been tuned to 1:6 for the ONT dataset as default. For each gap, up to ten candidates with the highest *QS* were chosen for error correction in order to suppress the data amount for further analysis. To further reduce the complexity and save computational resources, the overlapped candidates in the same long read were clipped and merged prior to the correction. Pilon[34] or Racon[45] could enhance the base-level accuracy of merged sequences. Pilon fixed individual base errors, small indels and local misassemblies with short but accurate NGS reads, while Racon also corrected sequencing errors by constructing a SIMD-accelerated partial-order alignment graph from long read's overlaps. The short

reads were aligned to candidates by minimap2 with the option *-k14 -w5 -n2 -m20 -s40 --sr --frag yes*.

The corrected candidates were realigned to the gap and scored again, and finally the one with the highest *QS* would be selected to fill the gap. The correction would benefit not only the single-base accuracy but also the final choice of candidates on the basis of the hypothesis that the *QS* of a candidate with higher-quality alignments would be increased due to the more precise mapping to the gap after error correction, while the candidate with relatively lower-quality alignments tends to fail to be mapped. We discarded the flanking wings of the winning candidate but kept all bases of scaftigs.

If the winner gave a negative filling information instead, then the gap would collapse to a single scaftig according to the overlapping relation. A portion of scaftigs have overlaps with others because of incorrect paths in the initial assembly graph or too aggressive scaftig extension strategy. However, a single-molecule long read spanning the gap has the ability to solve the overlapping if two scaftigs can be mapped to the correct positions. The candidate giving negative information was selected with extra strict criteria because large indels or homopoly-meric repeats in long reads tend to cause untruthful overlaps. Gaps without any corresponding candidates would fail to be closed, possibly because of the misassemblies in the draft assembly.

TGS-GapCloser is coded in C++ programing language (requires GCC 4.4+). It uses minimap2 to obtain alignments, and Pilon (requires Java runtime 1.7+) or Racon (requires GCC 4.8+) to correct candidate fragments. The algorithm automatically

determines gaps and tries to find the best matched long-read fragments to close gaps or merge adjacent scaftigs. To accelerate the gap closure without losing efficiency and accuracy, TGS-GapCloser only selects limited number of fragmented long reads as candidates for the following error correction and competition. Meanwhile, it reduces the computational complexity and improves the choosing accuracy by a concise but efficient scoring system (Table S3) and correction-induced mapping enhancement (Table S4). In addition, the aligner, minimap2 shows obvious improvements in speed and mapping accuracy for error-prone long reads[44], and further speed up the sequence alignment and enhance the gap-closing quality. The details in each step are individually recorded, including gap determination, mapping, candidate extraction, error correction, and candidate selection. The final output is reported in FASTA format, with a log file describing the detailed insertion/merging information to trace all the improvements.

Gap closure in the human genome

Three assemblies and two long-read datasets were used to benchmark the utility of TGS-GapCloser in gap closure and scaftig merging in the human genome. Using the same library, the whole genome was assembled by: (1) contigs by MaSuRCA + scaffolds by SLR-superscaffolder, (2) contigs by Mercedes + scaffolds by SLR-superscaffolder, and (3) contigs and scaffolds by Supernova to take full use of the barcoded long-range information. Although MaSuRCA itself can scaffold the contigs, the assembler does not utilize the SLR information, and generates relatively short

scaffolds. It is necessary to employ SLR-superscaffolder to obtain a comparable scaffold NG50 against Supernova. TGS long reads are ideal to build the bridge between the short-range NGS reads and long-range barcodes in consideration of the length and resolution of the medium/long-range information. To assess the efficiency, we extracted only ~10× coverage long reads from ONT Rel3 dataset with the claimed mean read identity of 82.73%[37] and Pacbio HiFi dataset with the claimed average read concordance of 99.8%[36], respectively. The long-read fragments from Rel3 were corrected by Pilon with NGS short reads while those from HiFi were corrected by Racon with long reads themselves. Figure 2 describes the improvements in the assembly evaluation given by QUAST[46] after gap closure. Up to 91.8% of total 191189, 94.8% of total 129408, and 86.8% of total 42359 gaps are successfully finished by TGS-GapCloser for three assemblies. The scaftig NG50 increases from 13.6kb to 610.6kb with Rel3 and to 243.7kb with HiFi for Assembly (1), 15.8kb to 682.4kb with Rel3 and to 173.7kb with HiFi for Assembly (2), and 113.0kb to 1229.2kb with Rel3 and to 1566.1kb with HiFi for Assembly (3). Additionally, the corresponding scaftig NGA50 is also improved from 13.4kb to 411.1kb with Rel3 and to 205.9kb with HiFi, 15.7kb to 418.2kb with Rel3 and to 153.2kb with HiFi, and 108.5kb to 734.2kb with Rel3 and to 849.7kb with HiFi, respectively. Note that our current algorithm does not split or merge input scaffolds to remain the existing long-range information. But the scaffold NG50 and NGA50 may vary due to the replacement of N's and combination of scaftigs. As listed in Table S2, the genome fraction against the reference is also increased by 1.4%, 3.2% and 0.4% with Rel3,

1.2%, 1.9% and 0.4% with HiFi for different inputs, indicating that the updated gaps are mapped to the new areas in the reference assembly. The application of Rel3 dataset increases the large-scale misassemblies (>1kb) induced by the filled sequences by 22.2% and 6.3% in Assembly (2) and (3) as expected, but decreases by 9.5% in Assembly (1) because the updated scaffolds can be mapped to the more precise positions against the reference. In spite of error correction, the local misassemblies (<1kb) still present an increment of 1.2-fold, 7.4-fold, and 1.1-fold dependent on the length and accuracy of filled sequences. The HiFi dataset with higher initial read accuracy represents fewer induced misassemblies and local misassemblies: -6.1% and 0.3-fold for Assembly (1), 13.1% and 1.3-fold for Assembly (2), 13.9% and 0.5-fold for Assembly (3). Overall, Rel3 closes more gaps to obtain better continuity than HiFi, but induces more assembly errors. This is because Rel3 keeps all the long reads (the longest >500kb) while HiFi folds the single-molecule fragments into ~13kb subreads to achieve better single-base accuracy (Figure S3). The performance of TGS-GapCloser is substantially dependent on both the length and the accuracy of input long reads, which are weights on each side of the balance for current single-molecule sequencing techniques.

BUSCO[47] (version 3.0.2) analysis indicates the possible enhancements for further bioinformatics analysis such as gene annotation after gap closure. The genome was queried against the vertebrata_odb9 database. It reveals that 90.5%, 89.7% and 94.1% of the expected vertebrate genes are complete for three assemblies with Rel3, and 90.4%, 85.3% and 94.0% with HiFi, both improved from the original 86.2%, 76.6%

and 90.7%, respectively.

Figure 2. Gap filling improvements and effects on the draft assemblies produced by TGS-GapCloser. (A) scaftig NG50, (B) scaftig NGA50, (C) number of remaining gaps, (D) genome fraction, (E) misassemblies and (F) local misassemblies for the human genome. (C) was calculated by directly counting while others were reported by QUAST.

Gap closure in the ultra large genome of ginkgo

The *Ginkgo biloba* is a best-known living fossil that has remained its form and structure over 270 million years, taking a unique position in the evolutionary tree of life[48]. We applied TGS-GapCloser to the chromosomal-level assembly[42] with ~10.5× coverage of Pacbio reads. The input assembly has been assigned to 13 chromosomes of 9,570,195,624 bp, with 613,821 gaps in total. In this case, the long reads are pre-corrected by Canu. Up to 71.6% of the gaps were closed, and the total assembly size without N was increased by 411,608,879 bp. The scaftig N50 was also enhanced from 57.1kb to 364.8kb. Note that many gap-closing tools have been benchmarked only for several bacterial and fungal genomes or small eukaryotes previously[28, 30, 49], and it is doubt that they could be applied to this ultra large genome using reasonable computing resources.

Validation of gap-closing sequences

As a sanity check, we mapped the gaps in input scaffolds to the human reference

assembly, generated ideally filled sequences, and compared them to the filled long-read fragments created by TGS-GapCloser. Note that the statistics of the filled gaps described here are different from that given by directly counting (Figure 2 (C)) since the gaps closed with scaftig overlapping are not judged. The evaluation listed in Table 1 is split into two parts: long read choosing accuracy and single base level accuracy. For the selection of fragments inserted by TGS-GapCloser, the validated PPV ranges from 98.1% to 62.0% for three assemblies, and the sensitivity from 96.4% to 51.2%. In total, gap-closing results with HiFi reads show relatively higher PPV due to its higher read accuracy, but lower sensitivity due to its shorter read length. The accuracy of Assembly (1) or (2) is better than that of Assembly (3), which has more small gaps. The result implies that TGS-GapCloser tends to fill large gaps.

In terms of single-base level accuracy, we calculated the Phred-like concordance QV with the method described in [36]. The QV of the inserted long-read fragments is improved after error correction. However, the overall QV of the assembly is decreased: the scaftig QV is down from 45.8 to 40.8 with Rel3 but to 42.1 with HiFi on average. The degree of accuracy decline is less obvious with HiFi reads after error correction, which is consistent with the higher PPV in the long-read selection. The final assemblies with >Q40 single-base quality are still comparable with or even better than most *de novo* TGS assemblies along with pre-error correction and polish[36, 37].

Table 1. Gap-closing accuracy statistics and computational consumptions for TGS-

GapCloser.

Performance of TGS-GapCloser for large genomes

We have presented a new tool for updating the genome assembly fast and accurately. For the human genome, it consumed as low as 155 CPU hours in total and 32 GB peak memory. The algorithm designs substantially reduce the time for read mapping and error correction. However, the gap closure with the NGS-based error correction for the inserted sequences (~189 hours on average) is much slower than that with the TGS-based correction (~15 hours on average). In contrast, the *de novo* assembly for 30× coverage of long reads requires ~40K CPU hours for ONT and ~62K CPU hours for Pacbio[37]. The computing consumption can be further compressed without error correction. It only took 541 CPU hours for the ginkgo genome using pre-corrected Pacbio reads. TGS-GapCloser requires only low coverage of expensive long reads without pre-error correction, making this approach more costly effective and suitable for research projects with small budgets.

Comparison with other gap-closing tools

We did not compare TGS-GapCloser to the NGS gap-closing tools because the utilization of long-read medium/long-range information can span the repetitive or other complicated regions in the assembly that *k*-mer-based extension cannot reach, and it congenitally creates better results. In this paper, we applied a series of published long-read gap closers, including PBJelly[12], FGAP[28], GMcloser[30],

Cobbler[31] and LR_Gapcloser[32], on the same Chr19 Mercedes+SLR-superscaffolder assembly with Rel3 reads, and systematically compared the performance.

The evaluation shows that TGS-GapCloser exhibits the best performance among six tools with this combination of inputs (Table 2). Its gap-closing efficiency is considerably higher than that of other tools, reducing the number of gaps from 2600 to 288, and enhancing the scaftig NG50 from 9.6kb to 194.5kb. The number of closed gaps by FGAP or LR_Gapcloser is second only to TGS-GapCloser. For LR_Gapcloser, the default number of gap-closing iterations is 3, and the scaftig NG50 increases to 157.2kb. FGAP also reduces the gap number to 458 and increases the scaftig NG50 to 127.4kb. On the other hand, PBJelly, GMcloser and Cobbler leave more than 1000 gaps and do not represent good improvements in the scaftig length. In terms of accuracy, TGS-GapCloser leads to the greatest increase in the scaftig NGA50 (16.0 folds to input) with fewer introduced misassemblies, which is at least 5.2 times that of other five tools. Although FGAP and LR_Gapcloser extend the scaftig NG50 longer than 100kb, the increasing number of misassemblies results in shorter scaftig NGA50. Most gap-closing tools were originally designed for error-corrected long reads or high-quality pre-assembled contigs, and as a result, their performances are somewhat unsatisfactory with such low coverage of raw ONT reads without error correction.

Table 2. Gap filling statistics for TGS-GapCloser and other gap-closing tools.

Input data	Unfilled gaps	Misassembly	Local misassembly	Scaffold NG50 (bp)	Scaffold NGA50 (bp)	Scaftig NG50 (bp)	Scaftig NGA50 (bp)	Runtime (min)	Peak memory (GB)
Draft Assemblies	2,600	176	126	1,561,142	196,307	9,687	9,464	/	/
TGS-GapCloser	288	187	324	1,426,438	383,995	194,512	149,166	12	16.37
PBJelly	1,730	664	741	1,240,439	83,803	29,715	19,247	3,137	9.93
FGAP	458	867	684	1,871,611	44,244	127,982	28,615	2,687	35.06
GMcloser	2,600	175	125	1,561,142	195,886	9,570	9,335	17,140	11.39
Cobbler	1,475	230	516	1,522,592	176,960	24,072	18,217	24	9.43
LR_Gapcloser	447	1,064	1,076	1,561,028	27,211	157,181	18,216	74	2.90

All datasets were run with 16 threads on the same machine. Note that QUAST accepts <10 continuous N's in the scaftig.

In addition, we measured the running time and memory consumption for each tool under the same operation conditions. TGS-GapCloser runs approximately 261-, 224-, 1428-, 2- and 6-fold faster than PBJelly, FGAP, GMcloser, Cobbler and LR_Gapcloser, respectively. The BLAST[29]-based GMcloser and FGAP, and the BLASR[50]-based PBJelly are the most time-consuming. The relatively higher memory requirement of TGS-GapCloser comes from the error correction.

LR_Gapcloser employs short-tag comparisons to avoid long-read alignments, and thus requires the least memory than others.

Effects of long-read coverage

It is worthwhile evaluating the effects of long-read coverage on the gap closure. We randomly extracted 1×, 5×, 10×, 20× and 29× coverages of mapped ONT Rel3 reads against the Chr19 reference, and individually applied them to the same Chr19 MaSuRCA +SLR-superscaffolder assembly by TGS-GapCloser using the same default parameters. As shown in Figure S1 (A), the number of closed gaps and the total filled bases grow with the increasing coverage, but saturate at ~10× coverage close to the level of theoretically filled gap number and bases. Surprisingly, the total time usage does not change much with the increasing coverage, while the peak memory presents an approximately linear growth in Figure S1 (B). With more long reads, Figure S1 (C) displays that the sensitivity of inserted sequences increases from 22.1% to 87.4% while the PPV remains similar. In terms of single-base level accuracy shown in Figure S1 (D), the average concordance QV of inserted sequences drops as more gaps are closed, but has ignorable effect on that of scaftigs. The result indicates that TGS-GapCloser enables a considerable number of gaps closed with high quality using low coverage of error-prone long reads. In contrast, a high-quality long-read assembly requires at least 30× sequencing coverage[51].

Improvements in the MHC region

The assembly of MHC region in the human genome has been advertised as a proof that TGS medium/long-range information can overcome the assembly difficulty of short reads and span the ~6 Mb region of high repetition and polymorphism[37]. It is located on the Chr6 containing all class I and II human leukocyte antigens genes, important to cancer and immunity studies[52]. We analyzed three assemblies before and after gap closure to investigate the contiguity and accuracy in this region as shown in Table 3. For Assembly (3), a portion of a single long scaffold (>29Mb) entirely covers the MHC region, while several portions of two or three scaffolds (0.6-27Mb) cover the region for Assembly (1) and (2). Gap closure with the ONT Rel3 dataset reduces the number of scaftigs in those scaffolds from 339 down to 31, 271 to 26, and 76 to12, respectively. It eliminates N bases from 15.2% of the total assembly down to 3.7% on average, and enhances the mapped genome fraction against the reference from 81.52% to 91.17%. As a result, the scaftig NG50 and NGA50 are improved from 46.7kb to 585.1kb, and 41.0kb to 300.4kb, which would effect the gene annotations, structural variation detection or single nucleotide polymorphism calling. In contrast, although TGS long reads resolve the MHC locus into a single or several contigs, the relatively short contig NGA50 (52.6kb), low genome fraction (59.86%) and numerous local misassemblies indicate that improving the accuracy in short-range information is still a challenge for TGS applications.

Table 3. Improved assemblies in the MHC region by TGS-GapCloser.

Future direction

A series of improvements for future versions of TGS-GapCloser have been put on the agenda. The selection of inserted sequences largely depends on the performance of the aligner. Although minimap2 performs well in most cases, the alignment gets more imprecise if the pairwise sequences share small overlaps. We hope that the problem will be solved by applying other aligners or extra parameter optimization. In addition, the computational consumption by error correctors or polishers is still considerable although our algorithm has tried to reduce the input data size as much as possible. But it is convenient to replace them with other error-correction tools when available. Long reads with higher quality are promised by ONT and Pacbio, which help us get rid of this annoying step. Last but not least, we default the input scaffolds including the orientation and order relations of scaftigs to retain the existing assembly information, but neglect possible assembly errors. We are planning to use the medium/ long-range information provided by TGS reads to correct the improper relation of scaftigs in the same scaffold and link different scaffolds if the overlapping is detected. Nevertheless, TGS-Gapcloser enables the combination of different genetic information with different lengths and resolutions, making it possible to complete the high-quality (ultra) large genome assembly.

Methods

Gap closing with other tools

We compared the performance of TGS-GapCloser with that of five TGS gap-closing tools, including PBJelly (version PBSuite_15.8.24), FGAP (version 1.8.1), GMcloser (version 1.6.2), Cobbler (version 0.6.1) and LR_Gapcloser (no version information available). Among them, some cannot close any gaps with default parameters using low coverage of error-prone long reads. As a result, FGAP was tuned to have the capability to close larger gaps (<100kb, default <500). For GMcloser, we used the example parameters for long reads from the manual. In addition, the parameters of Cobbler were also tuned according to the authors' guidance on GitHub. All other tools were run using the default parameters for the ONT type.

Validation of gap-closing results

We classified the elevation of the gap-closing accuracy in two levels: the selection of long reads and the single-base level. The former is determined by whether the algorithm can capture the best long read to close the corresponding gap, and has an effect on the detection of chromosomal variations, large relocations and inversions. The quality of error correction and the size of inserted long-read bases decide the single-base level accuracy, and would effect single-nucleotide polymorphisms or small insertion/deletion calling in short range.

QUAST[46] (version 5.0.2) not only reports length statistics for the assembly such as total length, scaffold NG50 and scaftig NG50, but also provides the mapping relations with the reference, including scaffold NGA50, scaftig NGA50, genome fraction,

misassemblies and local misassemblies. Note that QUAST splits the scaffold by continuous fragments of N's of length ≥ 10 as scaftig. To further assess the efficiency and accuracy, we aligned the reference assembly against the input scaffolds to generate the theoretically filled gap sequences with QUAST intermediate files, and compared them to the actually filled sequences by TGS-GapCloser with minimap2 (*-x map-ont*). Gaps that can be filled by the reference are chosen to evaluate the sensitivity and PPV. Note that gaps smaller than 100bp were filtered out. The sensitivity is defined as the ratio of the number of actually filled gaps that the reference also successfully fills to the total number of gaps that the reference can fill. The PPV is defined as the ratio of the number of actually filled gaps that can be uniquely matched to the reference-filled gaps to the total number of filled gaps by both. Note that TGS-GapCloser also completes gaps that the reference cannot fill using the new medium/long-range information provided by long reads, which cannot be easily judged. The single-base level accuracy was quantified by mapping the scaftigs in the assembly to the GIAB high-confidence regions in the reference genome GRCh37 to calculate the concordance QV with the method in [36], where the scaftigs were split into bins of 100kb, and those bins with >50% mapped length at >50% identity ratio were used to calculate the average concordance quality value. The QVs were expressed in Phred format.

BUSCO

To quantify the possible improvements for downstream bioinformatics analysis, we

ran BUSCO analysis for all the human assemblies against the vertebrata_odb9 and the gingko assemblies against the embryophyta_odb9 database. Note that we directly input the whole human assemblies, but split gingko ultra-long scaffolds (>1.1Gb) into several portions at the position of large gaps (>1kb) because the aligner tblastn[53] in BUSCO could not handle such long sequences. The additional random breakpoints in the original scaffolds would decrease the continuity, and affect the BUSCO benchmarking.

Availability of source code and requirements

Project name: TGS-GapCloser

Project home page: https://github.com/BGI-Qingdao/TGS-GapCloser

Operating system(s): Linux

Programming language: C++, shell

Other requirements: Racon, or SAMtools and Pilon are required to be pre-installed

License: GPLv3

RRID: SCR_017633

biotools ID: TGS-GapCloser

Conda Access: conda install -c bioconda tgsgapcloser

Availability of supporting data and materials

The human genome datasets and assemblies are available at:

stLFR reads: CNSA under accession ID CNP0000066;

ONT reads: https://github.com/nanopore-wgs-consortium/NA12878/blob/master/nanopore-human-genome/rel_3_4.md;

Pacbio reads: GIAB ftp://ftp.ncbi.nlm.nih.gov/giab/ftp/data/NA12878/PacBio_SequelII_CCS_11kb/HG001.SequelII.pbmm2.hs37d5.whatshap.haplotag.RTG.trio.bam;

Assemblies: CNSA under accession ID CNP0000796.

The ginkgo genome datasets and assemblies are available at CNSA under accession ID CNP0000796.

Additional files

Supplementary information contains the following information:

Figure S1: Effects of long read coverage on gap closure. (A) the number of filled gaps and bases, (B) wall-clock time and peak memory, (C) accuracy in long-read selection, and (D) accuracy at single-base level. All datasets were run with 16 threads.

Figure S2. Length distribution of gaps in draft scaffolds and that of TGS-GapCloser filled gap sequences.

Figure S3. Read length distribution for the input ONT Rel3 and Pacbio HiFi reads.

Table S1. Summary of the input assemblies in this work.

Table S2. Summary of the updated assemblies in this work.

Table S3. The effect of the scoring system on the candidate selection and the gap-closing performance.

Table S4. The effect of error correction on the candidate selection and the gap-closing performance.

Table S5. The effect of long-read coverage on the TGS assemblies and gap-closing results.

Table S6. Genomics dataset source.

Table S7. Control parameters used for different software tools.

Abbreviations

TGS: third-generation sequencing; GIAB: Genome in a Bottle; SLR: synthetic long reads; NGS: next-generation sequencing; Pacbio: Pacific Biosciences; ONT: Oxford Nanopore Techniques; OLC: Overlap-Layout-Consensus; PPV: positive predictive value; MHC: major histocompatibility complex; stLFR: single tube Long Fragment Reads; Chr19: Chromosome 19; Chr6: Chromosome 6; QS: quality score; SIMD: single-instruction-multiple-data; BUSCO: Benchmarking Universal Single-Copy Orthologs; QV: quality value; CNSA: CNGB Nucleotide Sequence Archive.

Competing interests

The authors declare that they have no competing interests.

Funding

This research was supported by the National Key Research and Development Program of China (Grant No. 2018YFD0900301-05) and the Qingdao Applied Basic Research Projects (Grant No. 19-6-2-33-cg).

Authors' contributions

M.X., L.G., and L.D. performed software design and implementation. M.X., L.G., S.G., O.W., and R.Z. contributed to data modeling, data curation, assembler benchmarking. M.X. wrote the draft manuscript and L.G., G.F., and L.D. contributed to manuscript editing. X.X., L.D., and X.L. supervised the project. M.X. and F.G. performed funding acquisition. All authors read and approved the final manuscript.

ACKNOWLEDGEMENTS

The authors are grateful for the advice from Hongmei Zhu, the support of Mercedes Assembler from Yinlong Xie and many other BGI-Shenzhen employees in the development of TGS-GapCloser. The data that support this study have been deposited into CNSA of CNGBdb with accession number CNP0000796 (https://db.cngb.org/cnsa/).

References

1. KA. W. DNA Sequencing Costs: Data from the NHGRI Genome Sequencing Program (GSP). 2014.
2. Branton D, Deamer DW, Marziali A, Bayley H, Benner SA, Butler T, et al. The potential and challenges of nanopore sequencing. Nat Biotechnol. 2008;26 10:1146-53. doi:10.1038/nbt.1495.
3. Schadt EE, Turner S and Kasarskis A. A window into third-generation sequencing. Hum Mol Genet. 2010;19 R2:R227-40. doi:10.1093/hmg/ddq416.
4. Peters BA, Kermani BG, Sparks AB, Alferov O, Hong P, Alexeev A, et al. Accurate whole-genome sequencing and haplotyping from 10 to 20 human cells. Nature. 2012;487 7406:190-5. doi:10.1038/nature11236.
5. Kaper F, Swamy S, Klotzle B, Munchel S, Cottrell J, Bibikova M, et al. Whole-genome haplotyping by dilution, amplification, and sequencing. Proc Natl Acad Sci U S A. 2013;110 14:5552-7. doi:10.1073/pnas.1218696110.
6. Amini S, Pushkarev D, Christiansen L, Kostem E, Royce T, Turk C, et al. Haplotype-resolved whole-genome sequencing by contiguity-preserving transposition and combinatorial indexing. Nat Genet. 2014;46 12:1343-9. doi:10.1038/ng.3119.
7. Zheng GX, Lau BT, Schnall-Levin M, Jarosz M, Bell JM, Hindson CM, et al. Haplotyping germline and cancer genomes with high-throughput linked-read sequencing. Nat Biotechnol. 2016;34 3:303-11. doi:10.1038/nbt.3432.
8. Wang O, Chin R, Cheng X, Wu MKY, Mao Q, Tang J, et al. Efficient and unique cobarcoding of second-generation sequencing reads from long DNA molecules enabling cost-effective and accurate sequencing, haplotyping, and de novo assembly. Genome Res. 2019;29 5:798-808. doi:10.1101/gr.245126.118.
9. Belton JM, McCord RP, Gibcus JH, Naumova N, Zhan Y and Dekker J. Hi-C: a comprehensive technique to capture the conformation of genomes. Methods. 2012;58 3:268-76. doi:10.1016/j.ymeth.2012.05.001.
10. Shelton JM, Coleman MC, Herndon N, Lu N, Lam ET, Anantharaman T, et al. Tools and pipelines for BioNano data: molecule assembly pipeline and FASTA super scaffolding tool. BMC Genomics. 2015;16:734. doi:10.1186/s12864-015-1911-8.
11. Eichler EE, Clark RA and She X. An assessment of the sequence gaps: unfinished business in a finished human genome. Nat Rev Genet. 2004;5 5:345-54. doi:10.1038/nrg1322.
12. English AC, Richards S, Han Y, Wang M, Vee V, Qu J, et al. Mind the gap: upgrading genomes with Pacific Biosciences RS long-read sequencing technology. PLoS One. 2012;7 11:e47768. doi:10.1371/journal.pone.0047768.
13. Schneider VA, Graves-Lindsay T, Howe K, Bouk N, Chen HC, Kitts PA, et al. Evaluation of GRCh38 and de novo haploid genome assemblies demonstrates the enduring quality of the reference assembly. Genome Res. 2017;27 5:849-64. doi:10.1101/gr.213611.116.
14. Li Y, Hu Y, Bolund L and Wang J. State of the art de novo assembly of human genomes from

massively parallel sequencing data. Hum Genomics. 2010;4 4:271-7. doi:10.1186/1479-7364-4-4-271.
15. Adams MD FC, Venter JC. Automated DNA Sequencing and Analysis Techniques. Academic Press; 1994.
16. Luo R, Liu B, Xie Y, Li Z, Huang W, Yuan J, et al. SOAPdenovo2: an empirically improved memory-efficient short-read de novo assembler. Gigascience. 2012;1 1:18. doi:10.1186/2047-217x-1-18.
17. Boetzer M and Pirovano W. Toward almost closed genomes with GapFiller. Genome Biol. 2012;13 6:R56. doi:10.1186/gb-2012-13-6-r56.
18. Tsai IJ, Otto TD and Berriman M. Improving draft assemblies by iterative mapping and assembly of short reads to eliminate gaps. Genome Biol. 2010;11 4:R41. doi:10.1186/gb-2010-11-4-r41.
19. Gao S, Bertrand D and Nagarajan N. FinIS: Improved in silico Finishing Using an Exact Quadratic Programming Formulation. In: Berlin, Heidelberg, 2012, pp.314-25. Springer Berlin Heidelberg.
20. Puranik R, Quan G, Werner J, Zhou R and Xu Z. A pipeline for completing bacterial genomes using in silico and wet lab approaches. BMC Genomics. 2015;16 Suppl 3 Suppl 3:S7. doi:10.1186/1471-2164-16-s3-s7.
21. Catasti P, Chen X, Mariappan SV, Bradbury EM and Gupta G. DNA repeats in the human genome. Genetica. 1999;106 1-2:15-36. doi:10.1023/a:1003716509180.
22. Ou S, Liu J, Chougule KM, Fungtammasan A, Seetharam AS, Stein JC, et al. Effect of sequence depth and length in long-read assembly of the maize inbred NC358. Nature Communications. 2020;11 1:2288. doi:10.1038/s41467-020-16037-7.
23. Watson M and Warr A. Errors in long-read assemblies can critically affect protein prediction. Nat Biotechnol. 2019;37 2:124-6. doi:10.1038/s41587-018-0004-z.
24. Ye C, Hill CM, Wu S, Ruan J and Ma ZS. DBG2OLC: Efficient Assembly of Large Genomes Using Long Erroneous Reads of the Third Generation Sequencing Technologies. Sci Rep. 2016;6:31900. doi:10.1038/srep31900.
25. Boetzer M and Pirovano W. SSPACE-LongRead: scaffolding bacterial draft genomes using long read sequence information. BMC Bioinformatics. 2014;15:211. doi:10.1186/1471-2105-15-211.
26. Zimin AV, Marçais G, Puiu D, Roberts M, Salzberg SL and Yorke JA. The MaSuRCA genome assembler. Bioinformatics. 2013;29 21:2669-77. doi:10.1093/bioinformatics/btt476.
27. Luo J, Lyu M, Chen R, Zhang X, Luo H and Yan C. SLR: a scaffolding algorithm based on long reads and contig classification. BMC Bioinformatics. 2019;20 1:539. doi:10.1186/s12859-019-3114-9.
28. Piro VC, Faoro H, Weiss VA, Steffens MB, Pedrosa FO, Souza EM, et al. FGAP: an automated gap closing tool. BMC Res Notes. 2014;7:371. doi:10.1186/1756-0500-7-371.
29. McGinnis S and Madden TL. BLAST: at the core of a powerful and diverse set of sequence analysis tools. Nucleic Acids Res. 2004;32 Web Server issue:W20-5. doi:10.1093/nar/gkh435.
30. Kosugi S, Hirakawa H and Tabata S. GMcloser: closing gaps in assemblies accurately with a likelihood-based selection of contig or long-read alignments. Bioinformatics. 2015;31 23:3733-41. doi:10.1093/bioinformatics/btv465.
31. Warren RL. RAILS and Cobbler: Scaffolding and automated finishing of draft genomes using

long DNA sequences. Journal of Open Source Software. 2016;1 7:116. doi:10.21105/joss.00116.

32. Xu G-C, Xu T-J, Zhu R, Zhang Y, Li S-Q, Wang H-W, et al. LR_Gapcloser: a tiling path-based gap closer that uses long reads to complete genome assembly. GigaScience. 2018;8 1 doi:10.1093/gigascience/giy157.
33. Koren S, Walenz BP, Berlin K, Miller JR, Bergman NH and Phillippy AM. Canu: scalable and accurate long-read assembly via adaptive k-mer weighting and repeat separation. Genome Res. 2017;27 5:722-36. doi:10.1101/gr.215087.116.
34. Walker BJ, Abeel T, Shea T, Priest M, Abouelliel A, Sakthikumar S, et al. Pilon: an integrated tool for comprehensive microbial variant detection and genome assembly improvement. PLoS One. 2014;9 11:e112963. doi:10.1371/journal.pone.0112963.
35. Weirather JL, de Cesare M, Wang Y, Piazza P, Sebastiano V, Wang XJ, et al. Comprehensive comparison of Pacific Biosciences and Oxford Nanopore Technologies and their applications to transcriptome analysis. F1000Res. 2017;6:100. doi:10.12688/f1000research.10571.2.
36. Wenger AM, Peluso P, Rowell WJ, Chang PC, Hall RJ, Concepcion GT, et al. Accurate circular consensus long-read sequencing improves variant detection and assembly of a human genome. Nat Biotechnol. 2019; doi:10.1038/s41587-019-0217-9.
37. Jain M, Koren S, Miga KH, Quick J, Rand AC, Sasani TA, et al. Nanopore sequencing and assembly of a human genome with ultra-long reads. Nat Biotechnol. 2018;36 4:338-45. doi:10.1038/nbt.4060.
38. Zook JM, Chapman B, Wang J, Mittelman D, Hofmann O, Hide W, et al. Integrating human sequence data sets provides a resource of benchmark SNP and indel genotype calls. Nat Biotechnol. 2014;32 3:246-51. doi:10.1038/nbt.2835.
39. Murigneux V, Rai SK, Furtado A, Bruxner TJC, Tian W, Ye Q, et al. Comparison of long read methods for sequencing and assembly of a plant genome. bioRxiv. 2020:2020.03.16.992933. doi:10.1101/2020.03.16.992933.
40. Deng L, Guo L, Xu M, Wang W, Gu S, Zhao X, et al. SLR-superscaffolder: a de novo scaffolding tool for synthetic long reads using a top-to-bottom scheme. bioRxiv. 2019:762385. doi:10.1101/762385.
41. Weisenfeld NI, Kumar V, Shah P, Church DM and Jaffe DB. Direct determination of diploid genome sequences. Genome Res. 2017;27 5:757-67. doi:10.1101/gr.214874.116.
42. Guan R. Updated draft genome assembly of Ginkgo biloba. Gigascience Database. 2019; doi:https://doi.org/10.5524/100613.
43. Guan Rea. Updated draft genome assembly of Ginkgo biloba. Gigascience Database. 2019; doi:https://doi.org/10.5524/100613
44. Li H. Minimap2: pairwise alignment for nucleotide sequences. Bioinformatics. 2018;34 18:3094-100. doi:10.1093/bioinformatics/bty191.
45. Vaser R, Sovic I, Nagarajan N and Sikic M. Fast and accurate de novo genome assembly from long uncorrected reads. Genome Res. 2017;27 5:737-46. doi:10.1101/gr.214270.116.
46. Gurevich A, Saveliev V, Vyahhi N and Tesler G. QUAST: quality assessment tool for genome assemblies. Bioinformatics. 2013;29 8:1072-5. doi:10.1093/bioinformatics/btt086.
47. Simao FA, Waterhouse RM, Ioannidis P, Kriventseva EV and Zdobnov EM. BUSCO: assessing genome assembly and annotation completeness with single-copy orthologs. Bioinformatics. 2015;31 19:3210-2. doi:10.1093/bioinformatics/btv351.

48. Guan R, Zhao Y, Zhang H, Fan G, Liu X, Zhou W, et al. Draft genome of the living fossil Ginkgo biloba. Gigascience. 2016;5 1:49. doi:10.1186/s13742-016-0154-1.
49. de Sa PH, Miranda F, Veras A, de Melo DM, Soares S, Pinheiro K, et al. GapBlaster-A Graphical Gap Filler for Prokaryote Genomes. PLoS One. 2016;11 5:e0155327. doi:10.1371/journal.pone.0155327.
50. Chaisson MJ and Tesler G. Mapping single molecule sequencing reads using basic local alignment with successive refinement (BLASR): application and theory. BMC Bioinformatics. 2012;13:238. doi:10.1186/1471-2105-13-238.
51. Ou S, Liu J, Chougule KM, Fungtammasan A, Seetharam A, Stein J, et al. Effect of Sequence Depth and Length in Long-read Assembly of the Maize Inbred NC358. bioRxiv. 2019:858365. doi:10.1101/858365.
52. Brandt DY, Aguiar VR, Bitarello BD, Nunes K, Goudet J and Meyer D. Mapping Bias Overestimates Reference Allele Frequencies at the HLA Genes in the 1000 Genomes Project Phase I Data. G3 (Bethesda). 2015;5 5:931-41. doi:10.1534/g3.114.015784.
53. Gertz EM, Yu YK, Agarwala R, Schäffer AA and Altschul SF. Composition-based statistics and translated nucleotide searches: improving the TBLASTN module of BLAST. BMC Biol. 2006;4:41. doi:10.1186/1741-7007-4-41.

Author notes

Mengyang Xu, Lidong Guo and Shengqiang Gu contributed equally to this work.

Table 1. Gap-closing accuracy statistics and computational consumptions for TGS-GapCloser.

Accuracy in long-read selection						
**Input data**	**No. of closed gaps**	**No. of closed gaps in theory**	**PPV (%)**	**Sensitivity (%)**	**Runtime (hours)**	**Peak memory (GB)**

MaSuRCA+SLR-superscaffolder+TGS-GapCloser (ONT)	75,629	74,353	96.6	96.3	259	50
MaSuRCA+SLR-superscaffolder+TGS-GapCloser (Pacbio)	74,321	74,353	98.2	89.8	13	33
Mercedes+SLR-superscaffolder+TGS-GapCloser (ONT)	58,938	61,267	97.7	93.4	145	51
Mercedes+SLR-superscaffolder+TGS-GapCloser (Pacbio)	52,116	61,267	98.4	75.6	11	32
Supernova+TGS-GapCloser (ONT)	22,563	24,760	62.0	51.2	163	74
Supernova+TGS-GapCloser (Pacbio)	26,919	24,760	76.1	61.2	20	38

Accuracy in single-base level

Input data	Input QV (Phred)	Output QV (Phred)

	No. of filled bases (bp)	No. of filled bases in theory (bp)	Raw long reads	Scaftigs	Filled long reads	Scaftigs
MaSuRCA+SLR-superscaffolder+TGS-GapCloser (ONT)	335,541,557	353,352,038	7.63	40.51	23.24	36.06
MaSuRCA+SLR-superscaffolder+TGS-GapCloser (Pacbio)	198,327,815	353,352,038	26.99	40.51	35.52	37.64
Mercedes+SLR-superscaffolder+TGS-GapCloser (ONT)	352,316,717	497,208,670	7.63	48.09	23.23	40.19
Mercedes+SLR-superscaffolder+TGS-GapCloser (Pacbio)	146,148,151	497,208,670	26.99	48.09	36.25	42.29
Supernova+TGS-GapCloser (ONT)	49,669,581	38,276,270	7.63	48.72	23.15	46.11
Supernova+TGS-GapCloser (Pacbio)	22,178,115	38,276,270	26.99	48.72	34.82	46.48

All datasets were run with 42 threads. Note that the peak memory consumption by Pilon or

Racon is not counted. The higher speed of runs using the Pacbio HiFi dataset mainly originates from the usage of Racon to correct fragments with long reads. Note that QUAST accepts <10 continuous N's in the scaftig.

Table 3. Improved assemblies in the MHC region by TGS-GapCloser.

	**MaSuRCA+SLR-superscaffolder+TGS-GapCloser**		**Mercedes+SLR-superscaffolder+TGS-GapCloser**		**Supernova+TGS-GapCloser**		**Ref.（33）**	
	draft	updated	draft	updated	draft	updated	Rel3	Rel5
No. of scaffolds (>1kb)	2	2	3	3	1	1	/	/
No. of Scaftigs/contigs (>1kb)	339	31	271	26	76	12	7	1
Non-N bases (bp)	5,293,785	5,907,069	4,134,156	5,445,373	5,831,980	5,988,090	5,739,339	5,628,041
No. of gaps	343	31	268	23	81	16	/	/

Scaffold NG50 (bp)	3,400,000	3,400,000	4,400,000	4,400,000	6,000,000	6,000,000	/	/
Scaffold NGA50 (bp)	232,462	396,537	182,662	429,613	649,591	534,616	/	/
Scaftig/contig NG50 (bp)	17,483	324,807	12,244	450,213	110,320	980,326	3,007,673	5,628,041
Scaftig/contig NGA50 (bp)	16,630	199,405	11,901	321,624	94,556	380,102	49,485	52,555
Genome Fraction (%)	82.801	92.623	67.869	85.609	93.887	95.292	62.521	59.855
No. of misassemblies	11	25	13	22	15	17	20	53
No. of local misassemblies	34	101	11	122	29	42	546	484

The statistical results were generated by QUAST. Note that QUAST accepts <10 continuous N’s in the scaftig/contig.

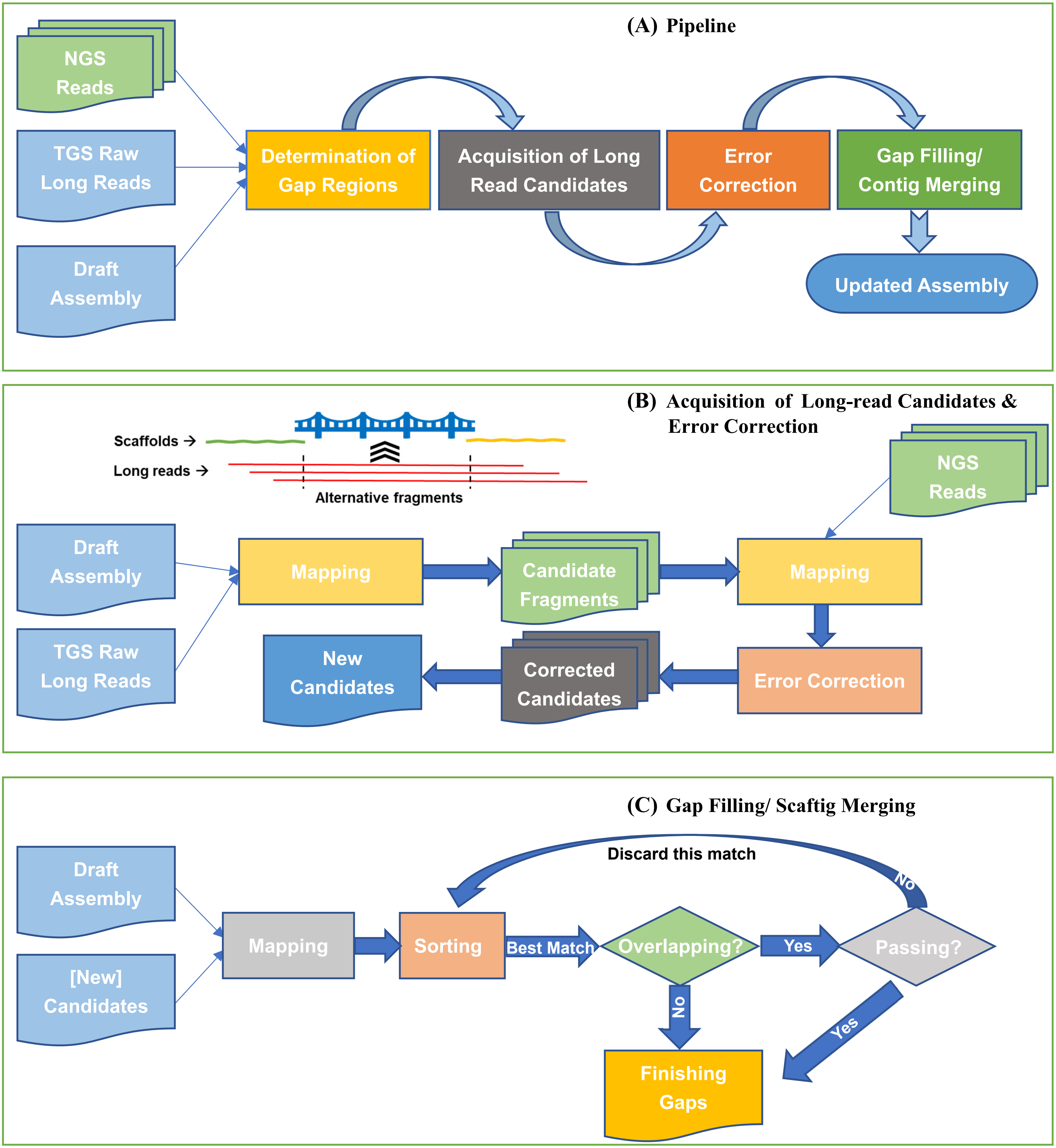
(A) Pipeline
NGS Reads
TGS Raw Long Reads
Draft Assembly
Determination of Gap Regions
Acquisition of Long Read Candidates
Error Correction
Gap Filling/ Contig Merging
Updated Assembly
(B) Acquisition of Long-read Candidates & Error Correction
Scaffolds →
Long reads →
Alternative fragments
NGS Reads
Draft Assembly
Mapping
Candidate Fragments
Mapping
TGS Raw Long Reads
New Candidates
Corrected Candidates
Error Correction
(C) Gap Filling/ Scaftig Merging
Discard this match
No
Draft Assembly
Mapping
Sorting
Best Match
Overlapping?
Yes
Passing?
[New] Candidates
No
Yes
Finishing Gaps

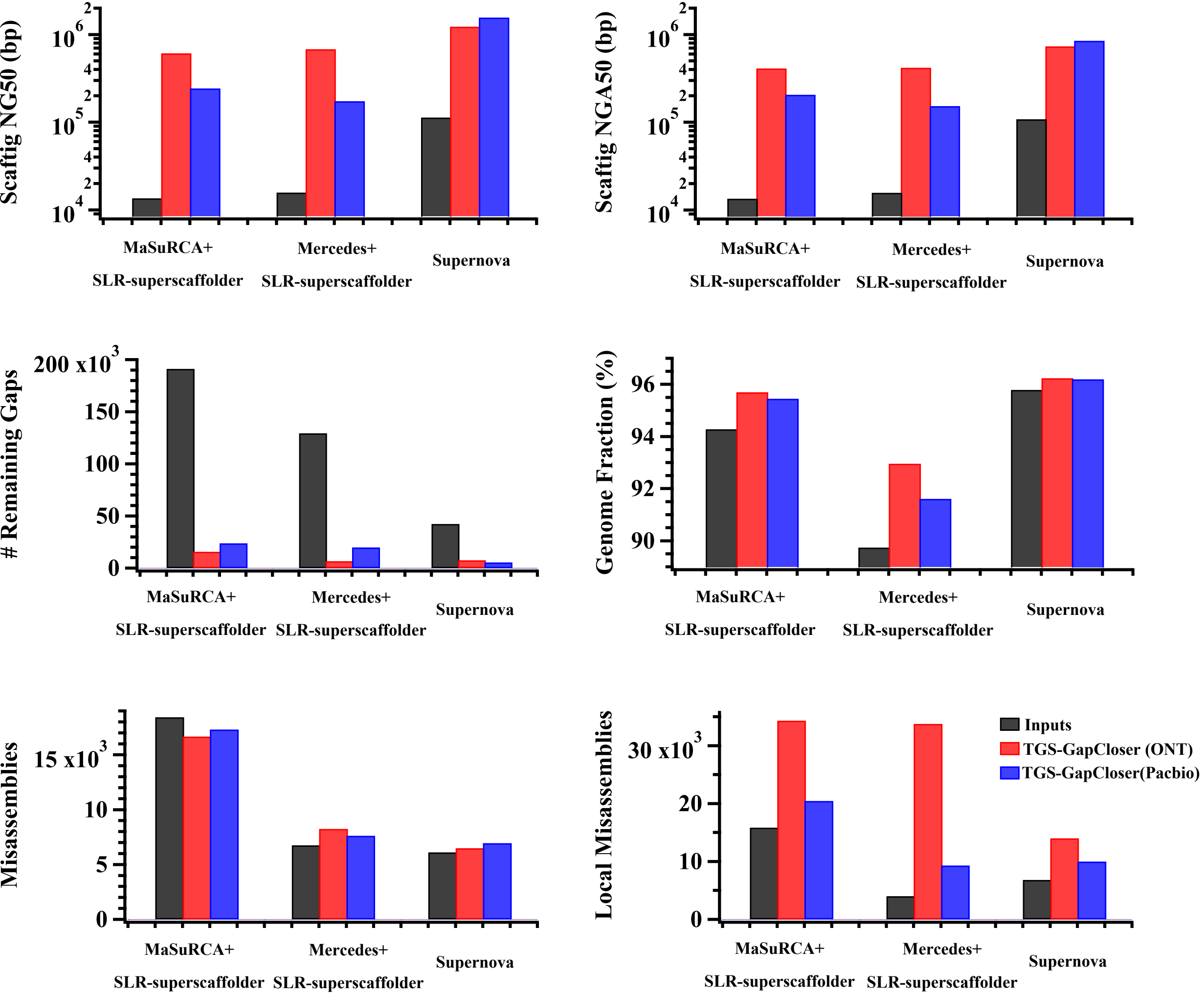

Scaftig NG50 (bp)
Scaftig NGA50 (bp)
Remaining Gaps
Genome Fraction (%)
Misassemblies
Local Misassemblies
MaSuRCA+
SLR-superscaffolder
Mercedes+
SLR-superscaffolder
Supernova
Inputs
TGS-GapCloser (ONT)
TGS-GapCloser(Pacbio)

Click here to access/download
Supplementary Material
SI_TGS-GapCloser_GigaScience_version9.docx

Cover Letter
Click here to access/download;Personal Cover;cover_letter.docx

February 2nd, 2020

Dear *GigaScience* Editor,

It is our great pleasure to submit the enclosed manuscript for your consideration of publishing on *GigaScience*. The brief of our submission is:

Title: TGS-GapCloser: A fast and accurate gap closer for large genomes with low coverage of error-prone long reads

Authors: Mengyang Xu, Lidong Guo, Shengqiang Gu, Ou Wang, Rui Zhang, Guangyi Fan, Xun Xu, Li Deng & Xin Liu

Manuscript type: Technical Notes

The application of third-generation sequencing technology has brought a revolution in life and biomedical fields, but suffers the problem of expense and accuracy. We developed a gap-closing software tool, TGS-GapCloser that utilizes only low depth of single molecule sequencing long reads to discover the complicated areas in large genomes that short reads cannot reach. We demonstrate that TGS-GapCloser improves the continuity, completeness of human genome and gingko ultra large genome without loss of accuracy. Comparing with mainstream long-read gap-closing tools, it can complete more gaps in input assemblies, but run incredibly faster. We believe that the TGS-GapCloser-based hybrid assembly strategy comprehensively employs assembly information to the utmost extent from various sequencing platforms, and improves the quality of downstream analysis of gene annotation. The low-depth requirement of expensive long reads makes this approach more costly effective and suitable for the community with small budgets, and readily enlarges the “big data” database.

All authors have declared that they have no competing interests, approved the contents of the manuscript and agreed with the submission to *GigaScience*. This manuscript is not under consideration for publication elsewhere and has been preprinted in bioRxiv only. We look forward to hearing from you soon. Your kind assistance on this is greatly appreciated!

Sincerely yours,

Mengyang Xu, Ph.D.

BGI-Research

BGI-Qingdao, BGI-SZ, Qingdao 266555, China
