# Supplementary material for: TGS-GapCloser: A fast and accurate gap closer for large genomes with low coverage of error-prone long reads
Source: Gigascience. 2020 Sep 7;9(9):giaa094. doi: 10.1093/gigascience/giaa094 (PMC7476103; doi:10.1093/gigascience/giaa094)
Supplement: giaa094_Supplemental_File [file giaa094_supplemental_file.docx]

**Supplemental Information****: TGS-GapCloser: A fast and** **accurate gap closer for large genomes with low coverage of error-prone long reads**

Mengyang Xu^1,2,4,#^, Lidong Guo^3,1,#^, Shengqiang Gu^3,1,#^, Ou Wang^4,6^, Rui Zhang^1^, Brock A. Peters^4,7^, Guangyi Fan^1,4^, Xin Liu^1,2,4,5^, Xun Xu^4,5^, Li Deng^1,2,4, *^ & Yongwei Zhang^4,7, *^

^1^BGI-Qingdao, BGI-Shenzhen, Qingdao 266555, China

^2^State Key Laboratory of Agricultural Genomics, BGI-Shenzhen, Shenzhen 518083, China

^3^BGI Education Center, University of Chinese Academy of Sciences, Shenzhen 518083, China

^4^BGI-Shenzhen, Shenzhen 518083, China

^5^China National GeneBank, BGI-Shenzhen, Shenzhen 518120, China

^6^MGI, BGI-Shenzhen, Shenzhen 518083, China

^7^Complete Genomics Inc., 2904 Orchard Pkwy, San Jose, California, 95134, USA

^#^These authors contributed equally to this work.

*Corresponding authors: Li Deng ([dengli1@genomics.cn](mailto:dengli1@genomics.cn)) and Yongwei Zhang ([zhangyongwei@genomics.cn](mailto:zhangyongwei@genomics.cn))

**Assemblies of MHC region**

Three human genome assemblies and the updated versions after gap closure were aligned to the 6-Mb-long reference in the MHC region using minimap2 (preset -x asm20). The MHC reference was extracted from the chr6:28000000-34000000 region in the GRCh38.p13 genome assembly. The corresponding mapped sequences were then extracted from a single or several long scaffolds based on their mapped positions against the reference for further QUAST analysis. The ONT Rel3 and Rel5 *de novo* assemblies were downloaded from <http://s3.amazonaws.com/nanopore-human-wgs/canu.30x.contigs.fasta> and <http://s3.amazonaws.com/nanopore-human-wgs/canu.35x.contigs.fasta>, and corresponding contigs in the MHC region were conduct in the same way. The mapped scaffolds/contigs with shorter than 50kb aligned length were ignored to avoid alignments that span the same region in the MHC reference. The polished assemblies of Rel3 and Rel5 show comparable results to the gap-closed assemblies.

**Gap length distribution**

Gaps in different draft human whole genome assemblies exhibit different length distributions as shown in Figure S2, however the majority is located in either positive or negative short length range (-1kb~1kb). The Supernova assembly represents more small gaps than others. The length distribution of filled sequences using ONT Rel3 dataset behaves highly similarly to that of corresponding inputs for different scenarios. But the small negative gaps (-500bp~0) are slightly overfilled by TGS-GapCloser, possibly because of the bad alignment quality for neighboring contigs with small overlaps found by the aligner.

**Figure S1: Effects of long read coverage on gap closure.** (A) the number of filled gaps ( left axis) and bases (right axis), (B) wall-clock time and peak memory, (C) accuracy in long-read selection, and (D) accuracy at single-base level. All datasets were run with 16 threads.


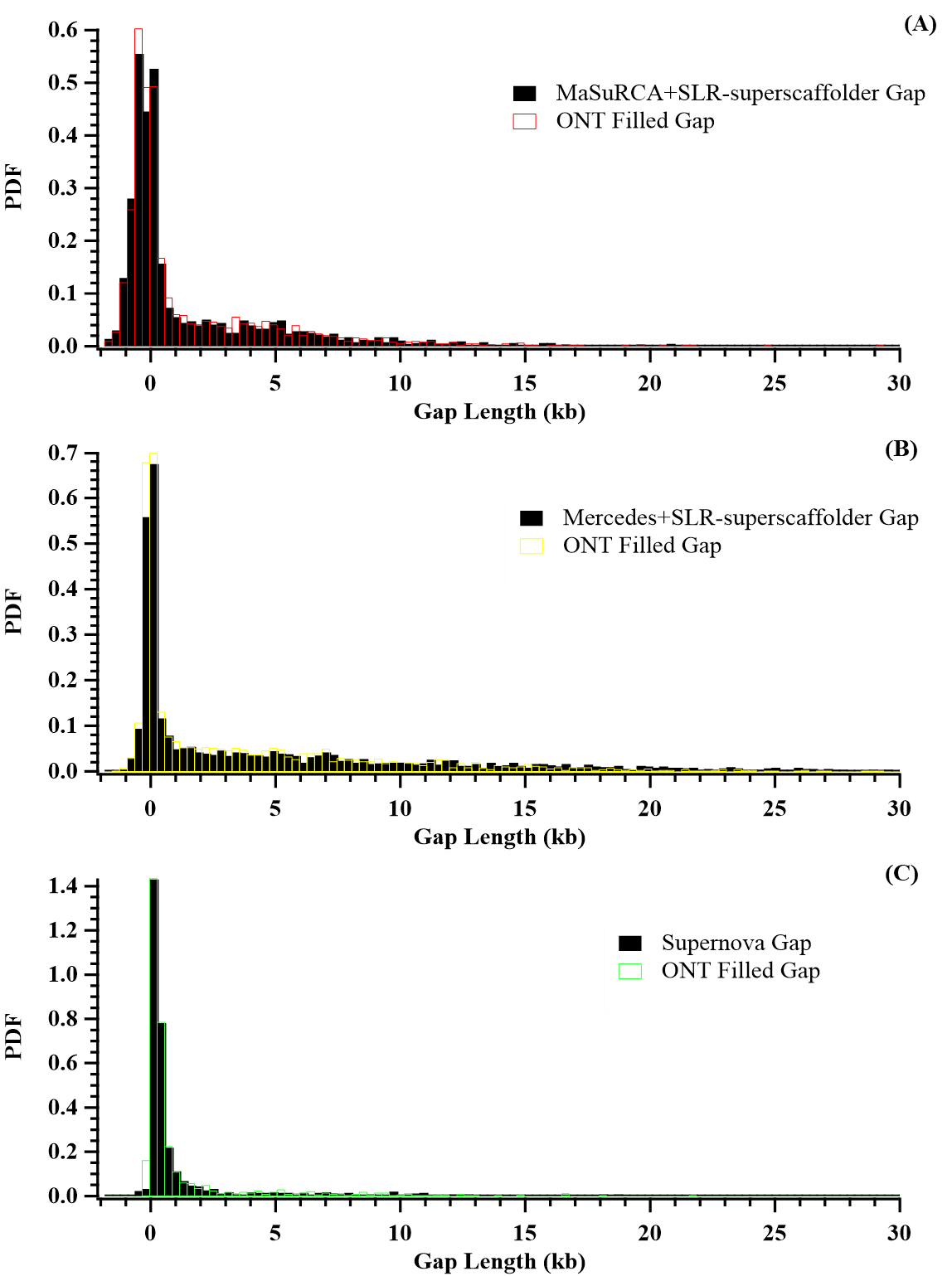


**Figure S2. Length distribution of gaps in draft scaffolds and that of TGS-GapCloser filled gap sequences.**

**
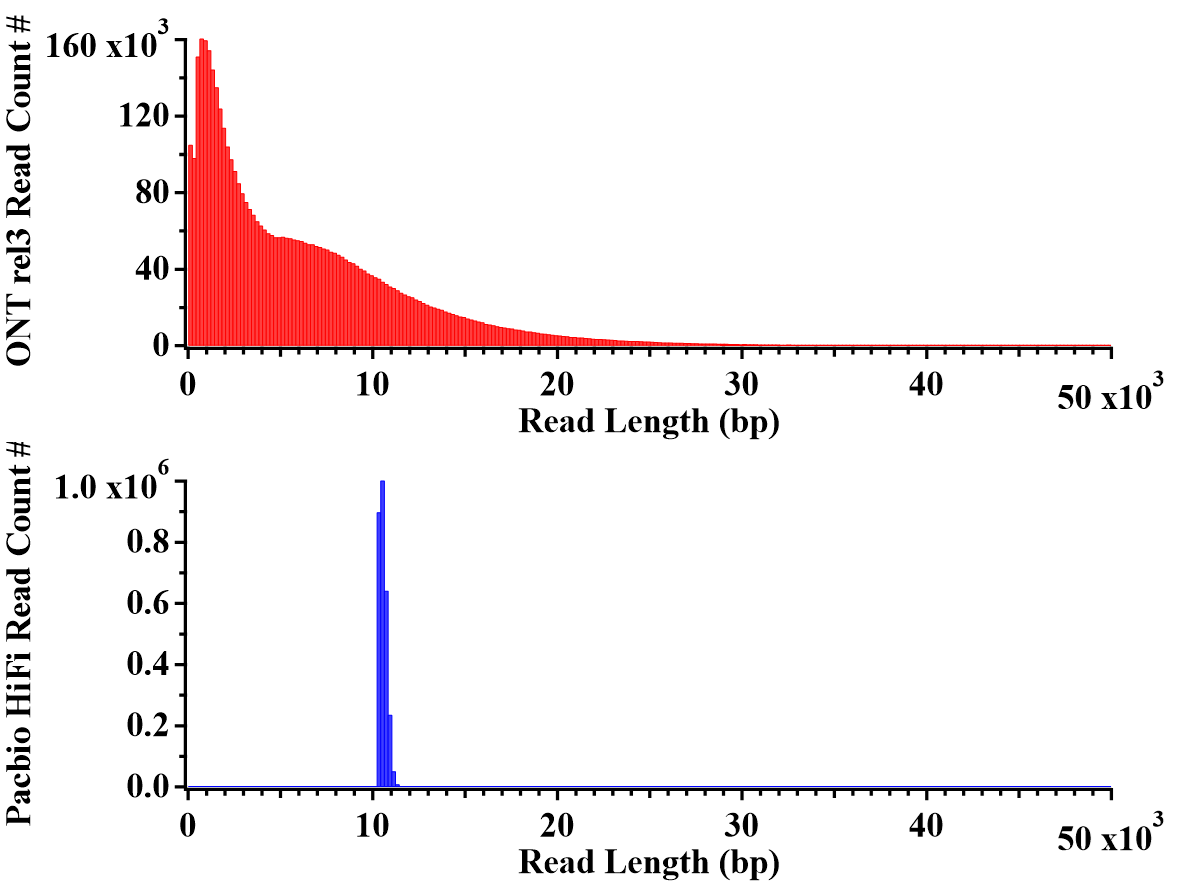
**

**Figure S3. Read length distribution for the input ONT Rel3 and Pacbio HiFi reads.**

**Table S1. Summary of the input assemblies in this work**

| **BUSCO** | / | / | / | C:86.2%[S:84.8%,D:1.4%],F:8.4%,M:5.4% | C:76.6%[S:75.6%,D:1.0%],F:13.8%,M:9.6% | C:90.7%[S:89.1%,D:1.6%],F:4.5%,M:4.8% | C:75.1%[S:66.0%,D:9.0%],F:8.8%,M:16.1% |
| --- | --- | --- | --- | --- | --- | --- | --- |
| **Local misassemblies** | 477 | 125 | 175 | 15,860 | 4,002 | 6,833 | / |
| **Misassemblies** | 170 | 175 | 122 | 18,425 | 6,749 | 6,110 | / |
| **Scaftig NGA50 (bp)** | 25,118 | 9,335 | 63,987 | 13,440 | 15,680 | 108,525 | / |
| **Scaftig NG50/N50 (bp)** | 25,792 | 9,570 | 66,000 | 13,569 | 15,757 | 113,000 | 57,099 |
| **Scaffold NGA50 (bp)** | 850,387 | 195,886 | 951,094 | 390,588 | 587,799 | 1,792,064 | / |
| **Scaffold NG50/N50 (bp)** | 8,696,596 | 1,561,142 | 8,378,184 | 18,898,312 | 11,997,047 | 26,329,970 | 724,988,903 |
| **No. of gaps** | 2,195 | 2,600 | 1,363 | 191,189 | 129,408 | 42,359 | 613,821 |
| **No. of Scaffolds** | 2,202 | 3,411 | 1,631 | 142,241 | 72,359 | 40,738 | 13 |
| **Genome Fraction (**%**)** | 97.86 | 87.64 | 97.42 | 94.28 | 89.74 | 95.79 | / |
| **Total length (bp)** | 62,941,506 | 66,644,779 | 57,459,696 | 3,345,341,888 | 3,201,977,072 | 2,855,133,582 | 9,570,195,624 |
| **Datasets** | MaSuRCA+SLR-superscaffolder | Mercedes+SLR-superscaffolder | Supernova | MaSuRCA+SLR-superscaffolder | Mercedes+SLR-superscaffolder | Supernova | SOAPdenovo2+Hi-C |
| **Organisms** | Human Chr19 | | | *Homo sapiens* (NA12878) | | | *Ginkgo biloba* |

**Table S2. Summary of the updated assemblies in this work**

| **BUSCO** | / | / | / | C:90.5%[S:89.3%,D:1.2%],F:5.2%,M:4.3% | C:90.4%[S:88.8%,D:1.6%],F:5.6%,M:4.0% | C:89.7%[S:88.4%,D:1.3%],F:5.8%,M:4.5% | C:85.3%[S:83.8%,D:1.5%],F:8.9%,M:5.8% | C:94.1%[S:92.4%,D:1.7%],F:2.7%,M:3.2% | C:94.0%[S:92.2%,D:1.8%],F:2.8%,M:3.2% | C:75.8%[S:66.6%,D:9.2%],F:8.5%,M:15.8% |
| --- | --- | --- | --- | --- | --- | --- | --- | --- | --- | --- |
| **Local misassemblies** | 636 | 324 | 297 | 34,309 | 20,462 | 33,770 | 9,300 | 14,028 | 10,010 | / |
| **Misassemblies** | 186 | 187 | 150 | 16,671 | 17,307 | 8,250 | 7,632 | 6,492 | 6,957 | / |
| **Genome fraction (%)** | 98.49 | 92.19 | 98.22 | 95.70 | 95.45 | 92.96 | 91.61 | 96.24 | 96.20 | / |
| **No. of filled bases (bp)** | 2,759,626 | 7,750,957 | 1,254,168 | 272,947,305 | 137,926,293 | 336,390,884 | 130,426,390 | 48,546,731 | 21,089,536 | 411,608,879 |
| **No. of closed gaps** | 2,098 | 2,278 | 1,156 | 175,437 | 167,259 | 122,626 | 109,548 | 34,831 | 36,757 | 439,641 |
| **Contig NGA50 (bp)** | 615,735 | 149,166 | 701,708 | 411,129 | 205,868 | 418,197 | 153,169 | 734,168 | 849,699 | / |
| **Contig NG50/N50 (bp)** | 1,499,980 | 194,512 | 1,550,347 | 610,644 | 243,696 | 682,438 | 173,738 | 1,229,152 | 1,566,131 | 364,800 |
| **Input data** | MaSuRCA+SLR-superscaffolder+TGS-GapCloser (ONT) | Mercedes+SLR-superscaffolder+TGS-GapCloser (ONT) | Supernova+TGS-GapCloser (ONT) | MaSuRCA+SLR-superscaffolder+TGS-GapCloser (ONT) | MaSuRCA+SLR-superscaffolder+TGS-GapCloser (Pacbio) | Mercedes+SLR-superscaffolder+TGS-GapCloser (ONT) | Mercedes+SLR-superscaffolder+TGS-GapCloser (Pacbio) | Supernova+TGS-GapCloser (ONT) | Supernova+TGS-GapCloser (Pacbio) | SOAPdenovo2+Hi-C+TGS-GapCloser (Pacbio) |
| **Organisms** | Human chr19 | | | *Homo sapiens* (NA12878) | | | | | | *Ginkgo biloba* |

**Table S3. The effect of the scoring system on the candidate selection and the gap-closing performance.**

| **Candidate selection stratagem** | **Closed Gaps** | **Choosing PPV (%)** | **Choosing Sensitivity (%)** | **Scaffold NGA50 (bp)** | **Scaftig NG50 (bp)** | **Scaftig NGA50 (bp)** | **Misassemblies** | **Local Misassemblies** | **Runtime (min)** | **Peak Memory (GB)** |
| --- | --- | --- | --- | --- | --- | --- | --- | --- | --- | --- |
| Input scaffolds | - | - | - | 850,387 | 25,792 | 25,118 | 170 | 477 | - | - |
| Random | 2,142 | 64.0 | 91.7 | 484,221 | 1,782,406 | 407,918 | 409 | 714 | 14.7 | 17.17 |
| Based on alignment length only | 2,141 | 79.4 | 91.8 | 765,783 | 2,178,210 | 529,413 | 337 | 657 | 15.0 | 17.16 |
| Based on identity ratio only | 2,145 | 93.3 | 92.3 | 825,103 | 2,176,878 | 540,122 | 234 | 628 | 14.9 | 17.20 |
| a: b =1:1 | 2,142 | 93.3 | 92.3 | 825,125 | 1,785,395 | 638,481 | 226 | 637 | 15.1 | 17.18 |
| a: b =1:6 | 2,143 | 94.3 | 92.3 | 883,840 | 1,784,955 | 687,191 | 212 | 625 | 15.0 | 17.22 |
| a: b =1:10 | 2,142 | 95.0 | 92.2 | 884,342 | 1,785,368 | 669,905 | 223 | 638 | 15.0 | 17.19 |
| a: b =6:1 | 2,142 | 87.5 | 91.5 | 766,583 | 2,176,999 | 507,643 | 305 | 642 | 15.1 | 17.19 |

**Note: Use ONT reads to close gaps in the Chr19 Masurca+SLR_Superscaffolder assembly. To fully test the effect of the scoring, min_idy and min_match were set to zero. The inserted sequences were corrected by Racon. All other parameters remained the same.**

**Table S4. The effect of error correction on the candidate selection and the gap-closing performance.**

| **# indels per 100 kbp** | 45 | 342 | 234 |
| --- | --- | --- | --- |
| **# mismatches per 100 kbp** | 151 | 344 | 195 |
| **Local Misassemblies** | 477 | 731 | 625 |
| **Misassemblies** | 170 | 219 | 212 |
| **Scaftig NGA50 (bp)** | 25,118 | 487,175 | 687,191 |
| **Scaftig NG50 (bp)** | 25,792 | 2,162,843 | 1,784,955 |
| **Scaffold NGA50 (bp)** | 850,387 | 663,651 | 883,840 |
| **Choosing Sensitivity (%)** | - | 92.2 | 92.3 |
| **Choosing PPV (%)** | - | 8.7 | 94.3 |
| **Closed Gaps** | - | 2,143 | 2,143 |
| **Candidate correction stratagem** | Input scaffolds | Without correction | With correction |

**Note: Use ONT reads to close gaps in the Chr19 Masurca+SLR_Superscaffolder assembly. To fully test the effect of error correction, min_idy and min_match were set to zero. Ratio a: b was set to 1:6. All other parameters remained the same.**

**Table S5. The effect of long-read coverage on the TGS assemblies and gap-closing results.**

**Table S6. Genomics dataset source.**

| **Source** | Extracted from *H. sapiens,* available via the GigaDB | Extracted from *H. sapiens,* available via the GigaDB | Extracted from *H. sapiens,* available via the GigaDB | Extracted from *H. sapiens,* available via the GigaDB | <ftp://ftp.cngb.org/pub/CNSA/CNP0000066/CNS0007594/CNX0005851/CNR0006062/> | <https://github.com/nanopore-wgs-consortium/NA12878/blob/master/nanopore-human-genome/rel_3_4.md> | <ftp://ftp.ncbi.nlm.nih.gov/giab/ftp/data/NA12878/PacBio_SequelII_CCS_11kb/HG001.SequelII.pbmm2.hs37d5.whatshap.haplotag.RTG.trio.bam> | <ftp://ftp-trace.ncbi.nih.gov/1000genomes/ftp/technical/reference/phase2_reference_assembly_sequence/hs37d5.fa.gz> | <ftp://ftp.cngb.org/pub/CNSA/CNP0000796/CNS0191733/CNX0142212/CNR0175149/female.all.fa.gz.fastq.gz> |
| --- | --- | --- | --- | --- | --- | --- | --- | --- | --- |
| **Avg. read length (bp)** | PE100 | 6,671 | 9,890 | 59,128,983 | PE100 | 6,434 | 10,552 | 36,482,032 | 10,722 |
| **Total bases** | 4,301 Mb | 1,673 Mb | 1,685 Mb | 59,128,983bp | 660 Gb | 30 Gb | 30 Gb | 3.1 Gb | 126 Gb |
| **Total reads** | 43,014,410 | 250,811 | 170,394 | 1 | 2,071,968,776 | 4,662,428 | 2,500,000 | 86 | 11,787,154 |
| **Datatype** | stLFR | ONT | Pacbio | Reference | stLFR | ONT | Pacbio | Reference | Pacbio |
| **Organisms** | Human Chr19 | | | | *Homo sapiens* (NA12878) | | | | *Ginkgo biloba* |

**Table S7. Control parameters used for different software tools.**

| **Software** | **Parameters** |
| --- | --- |
| TGS-GapCloser (ONT) | *--pilon PATH/pilon.jar --ngs short_reads.fq* |
| TGS-GapCloser (Pacbio) | *--racon PATH/racon --tgstype pb* |
| MaSuRCA | *GRAPH_KMER_SIZE=63, cgwErrorRate=0.15* |
| Mercedes | *Default* |
| SLR_superscaffolder | *MST_BIN_SIZE=7000, HT_BIN_SIZE=3500, CLUSTER=0.1, PE_SEED_MIN=200* |
| Supernova | *--nopreflight* |
| PBJelly | *--minMatch 8 --minPctIdentity 70 --bestn 1 --nCandidates 20 –noSplitSubreads* |
| FGAP | *-R 100000 -I 100000 –p 1 –z 0 –g 0 –t 16* |
| GMcloser | *-lr -l 100 -i 400 -d 40 -f fasta -n 16 -nc 16 -c* |
| Cobbler | *-l 1 -g 500 -d 1000 -i 0.8* |
| LR_Gapcloser | *-s nanopore -t 16 –c 0.8 –r 3* |
| QUAST | *-m1000 -s --fast --fragmented* |
| BUSCO | *-l vertebrata_odb9 −m geno −sp human* |
| Canu | *minInputCoverage=0 stopOnLowCoverage=0 useGrid=false genomeSize=60m maxThreads=16 -nanopore* |
