# Supplementary material for: TGS-GapCloser: A fast and accurate gap closer for large genomes with low coverage of error-prone long reads
Source: Gigascience. 2020 Sep 7;9(9):giaa094. doi: 10.1093/gigascience/giaa094 (PMC7476103; doi:10.1093/gigascience/giaa094)
Supplement: giaa094_Response_to_Reviewer_Comments_Original_Submission [file giaa094_response_to_reviewer_comments_original_submission.pdf]

Author's Response To Reviewer Comments

Close

Dear Editor,
Thank you for giving us the opportunity to submit a revised draft of the manuscript entitled "TGS-GapCloser: A fast and accurate gap closer for large genomes with low coverage of error-prone long reads." (GIGA-D-20-00014) to GigaScience. We appreciate the time and effort that you and the reviewers have dedicated to providing your valuable feedback on the manuscript. We are grateful to the reviewers for their insightful comments, and have incorporated changes to reflect most of the suggestions. Here is a point-by-point response to the reviewers' comments and concerns.

Response to Reviewer #1:
General Comment: The authors present a new method for gap closing with low coverage raw long reads. Experiments on draft genomes with contigs assembled from short reads and scaffolds from long range reads show the tool works well, and more efficiently compared to other tools. The manuscript overall is written and organized well. But I have the following concerns:

Response: We appreciate the reviewer's positive feedback about the efficiency of the software tool, and the organization and quality of the manuscript.

Comment 1: With more long reads sequenced, more genomes are directly assembled from long reads and then scaffolded or phased together with HiC and/or linked reads. Even though the cost is higher than short reads, different from applications like SV calling, genome assembly is done once and most of the cases the cost could be tolerated. The target application of the proposed method is using low coverage long reads to fill the gaps on draft genome assembled from short reads. The authors need to clearly define how "low" coverage they could perform better than with direct assembly from long reads. The authors show the performance of their tool on different coverage of long reads, but only on the draft genome assembled from short reads. How about the genome directly assembled from long reads? Say at 20X? If it is already good enough, then no need for gap closing at this coverage. This is pretty important as it defines the potential roles the proposed method could play.

Response: Thank you for raising this important point here. The relatively high cost of de novo assembly from long reads is from both expensive sequencing and high computing consumption, in spite of much effort to decrease the sequencing price and simplify the computation. A recent discussion of the effect of sequence depth and length in long-read assembly (Ou S, et al, Nature Communications. 2020;11 1:2288. doi:10.1038/s41467-020-16037-7) suggest that >30× sequencing coverage and >11kb N50 read length are required to obtain a high-quality assembly.
Actually, we extracted 1×, 5×, 10×, and 20× sequencing depth (coverage) of ONT reads for the human chromosome 19 and separately assembled them by Canu. The results indicate that those long reads cannot be directly used to assemble the whole chromosome (contig N50~40kb, and reference genome coverage ~30.7% with 20x sequencing depth) although the assembly is sharply improved with increasing depth. In contrast, TGS-GapCloser exhibits obvious assembly improvements (scaftig N50 ~454kb, and reference genome coverage~98.2%with only ~10× sequencing depth) on the basis of stLFR assembly (scaftig N50 ~27kb, genome coverage~97.9%). The comparison was summarized in Table S5.
In this study, we expect that the utilization of TGS long reads acts as a significant part in a hybrid assembly pipeline, which benefits from advantages of different sequencing techniques instead of TGS alone.

Comment 2: The major advantages in speed and memory cost over other tools come from using other third party tools that perform good, like minimap2, while the compared tools use slow tools like "blast". It's not from method innovation or better algorithm design, although selecting proper tools is also important.

Response: We agree that minimap2 is faster than BLAST or BLASR, and the choice of the aligner plays an important role in the performance. In our work, we also tried to speed up the gap-closing process through specific designs such as: 1. Fragmenting long reads as candidates and limiting the number of candidates corresponding to each gap for correction and competition to reduce the computing amount (30Gb long reads for human decreased to 1.9Gb candidates), and 2. A concise but efficient scoring system of candidates to reduce the computational complexity (Table S3).
In our tests, TGS-GapCloser is less time-consuming than BLASR-based PBJelly, BLAST-based FGAP, and the newly added minmap2-based Cobbler as suggested by Reviewer #2 (Table 2). It is difficult to owe the speed to the aligner only because their algorithm designs and best applicable scopes are different. The comparison demonstrates that TGS-GapCloser focuses more on the accuracy of the long-read candidate selection. This discussion was added to the section “Algorithm and implementation of TGS-GapCloser”, current version, page 12, line 2-9.

Comment 3: HG001 has HiFi long reads released by GIAB from last year (2019). The authors may consider switching to it.

Response: As suggested by the reviewer, we replaced the Pacbio HiFi reads of HG002 with that of HG001/NA12878 obtained from GIAB, and updated the gap-closing results in the manuscript. The improvements of the genome assembly after gap closure are comparable. On average, there are 2% increase in scaftig NG50, and 3% increase in scaftig NGA50 after the replacement for the three human assemblies. The effect on BUSCO results is almost ignorable.

Response to Reviewer #2:
Comment 1: The authors present a novel gap-filling algorithm that utilizes low coverage long read data, useful for augmenting existing assemblies. The algorithm appears sound and would be very useful for the bioinformatics community and experimental design and measurements/evaluations, for the most part, seem well designed. However, some more evaluations and comparisons to more tools may be needed. Also, the manuscript has some issues with the quality of the writing.
The writing of the manuscript needs work. Interestingly, it appears that attempts at correction were done but the manuscript seems like it had been proofread and edited by someone who didn't understand the content or just run through grammar checking software without consideration of the content, resulting in a manuscript that was somewhat difficult to read and inaccurate in some places. Giving the authors the benefit of the doubt it is possible to piece together the reasonable intent within the writing, but this takes a great deal of effort and guesswork.
For example, the problems with writing the paper start at the very beginning in the background of the abstract which states that "gap-closing tools suffer multi-alignments and high error rates". The authors likely intend for it to read something like "the long reads suffer from high error rates which reduce the performance of current long read based gap closing tools". Following this statement, they claim that this results in a "huge time and money costs" but these issues are not logically a direct result of the previous point. Perhaps the authors intended to state something like "due to the poor performance of these tools, high long read coverage is needed resulting in huge time and monetary costs". This kind of issue is just the tip of the iceberg of problems related to the overall writing of the paper and the authors need to be more careful in their writing in general to make this manuscript of publishable quality.

Response: We appreciate the positive feedback from the reviewer about the algorithm and the comments about the writing quality. We have carefully investigated the confused part mentioned above and gone through the whole manuscript, and realized that the reason is because we tried to express our opinion but extend to other points of view with few words. Thus, we split the sentence to make it more logically sound, and only focus on the closely related points. The main changes have been made as follows:
1) Abstract: Background, original version, page 2, line 3-6: “Despite benefiting from the medium/long-range information of single molecule sequencing techniques, current gap-closing tools to enhance assemblies suffer multi-alignments and high error rates, resulting in huge time and money costs, especially for large genomes.” To “Despite benefiting from the medium/long-range information, the employment of single molecule sequencing techniques to enhance assemblies suffers from the substantial sequencing cost and computational consumption, especially for large genomes (>1Gb).”
2) Findings: Introduction, original version, page 3, line 16-18: “However, all the finished assemblies are imperfect, even for human and model organisms, which contain gaps of unknown nucleic acids (represented by Ns).” To “However, the finished assemblies for human or other large organisms remain

imperfect, which contain gaps of unknown nucleic acids (represented by N's)[1, 2]. "
3) Findings: Introduction, original version, page 4, line 4-6: "But the manual or semi-automated processes limit the applications in consideration of huge costs." To "But the sequencing and labor costs hindered the manual or semi-automated gap-closing processes[1]."
4) Findings: Introduction, original version, page 5, line 11-17: "However, most tools mentioned above share the same crucial shortcoming: they function well only with pre-error-corrected or simulated long reads. It hampers the application because the error correction needs sufficient coverage of expensive long reads or extra short reads, and requires huge time and memory consumption, but usually splits long reads into short fragments and loses valuable assembly length information, not readily usable for large genomes." To "These tools have been widely used to close gaps with TGS long reads, but their efficiencies and accuracies are substantially dependent on the quality of input long reads. PBJelly improves the quality of inserted long reads through local assembly, but requires sufficient coverage. Other tools bypass the problem of input quality, and require or recommend pre-error corrected long reads or pre-assembled contigs. However, the additional assembly or correction for all the reads prior to gap closure needs adequate coverage of expensive long reads or extra short reads, and requires huge time and memory consumption, especially for large genomes. In addition, the correction algorithms might trim ambiguous segments[3] and split long reads into short fragments[4] due to the undetermined bases, thus losing valuable length information."
5) Findings: Introduction, original version, page 6, line 5-6: "High error rate and the existence of repeats may increase the probability of large misassembly events." To "The misalignments of long reads against the scaffolds owing to base-calling errors or repeats might increase the probability of large misassembly events. An effective scoring mechanism prevents the gap-closing tools from making wrong choice of filled fragments to some extent."
6) Findings: Algorithm and implementation of TGS-GapCloser, original version, page 9, line 20-22: "The alignment amount and quality determine the efficiency and accuracy of gap closure. Thus, all alignments were filtered based on the alignment length and identity ratio." To "The quantity and quality of candidates determine the efficiency and accuracy of gap closure. Thus, we designed a scoring system of candidates for quality control and filtration based on the length and identity ratio (matched bases/ aligned bases) of the alignment between a long-read candidate and flanking scaftig ends in the gap."
7) Findings: Algorithm and implementation of TGS-GapCloser, original version, page 10, line 21-22: "…a candidate with higher-quality alignments could be mapped to a more precise position in the reference…" to "…the QS of a candidate with higher-quality alignments would be increased due to the more precise mapping to the gap after error correction …"

Comment 2: The mention of the correctness of the assembly (q46) in the manuscript is somewhat meaningless unless compared to the quality before the tool was run. It might also make more sense if the authors state the correctness of only the filled regions, as this quality metric is likely washed out by the overall assembly quality.

Response: We agree that the single-base accuracy of the inserted sequences is washed out by that of the overall assembly because of the small ratio. We have, accordingly, added the quality of the inserted sequences to the abstract and emphasized the changes in both inserted and overall quality after the gap closure in Table 1. Although the corrected long-read fragments decrease the overall quality, they are improved from Q17 for raw to Q29 at last on average. This is a result of the accurate selection of long read to close the gap and the single-base level error correction.
As we noted in our response to Reviewer #1, TGS-GapCloser aims at improving the assembly quality as an important part of the comprehensive utilization of advantages from different techniques. The change in the final overall correctness induced by inserted sequences has a great influence on the quality of follow-up bioinformatics analyses. Thus, we retain the description of the overall correctness in the abstract but mark the contribution from the inserted sequences as suggested by the reviewer.

Comment 3: Contig NG50 was specified in the abstract but gap-filling typically is performed on scaffolds and one would not expect NG50 to increase substantially after gap-filling. Did the authors mean scaftig NG50? Better yet, perhaps scaftig NGA50 should be stated in the abstract instead. I noticed scaftigs seemed to be mentioned in the manuscript equating them to contigs; to prevent confusion consider replacing all instances of contig with scaftig.

Response: Thank you for pointing out the difference between contig and scaftig. As suggested by the reviewer, the "scaftig" is used to replace "contig", and refers to the continuous sequence within a scaffold without N's in the current main text.

Comment 4: It seems strange to me that various tools like LR-Gapcloser were not even benchmarked and merely stated that they "did not show any obvious improvements in efficiency and accuracy". Indeed, if PB-Jelly published in 2012 was compared, why were these other tools omitted? Other tools omitted from comparisons are Cobbler (Warren et al. 2016), GMcloser (Kosugi et al. 2015) and others may exist all of which should be considered in the comparisons unless a compelling reason they did not can be convincingly stated.
If the tools cannot be run due to resource limitations, authors should at least make this clear by benchmarking on smaller datasets and show how resource usage scales or at the very least state the algorithm does not run given their resources (explaining possible reasons why it does not scale). Unless the authors can definitively prove that these tools are not worth benchmarking as they have already shown performance less than the current state-of-the-art tools (to which TGS-GapCloser is compared to) in other publications in almost all dimensions, it doesn't make sense to me that these tools should be omitted from the comparisons.

Response: As suggested by the reviewer, we added three latest tools to the comparison, including GMcloser, Cobbler and LR_Gapcloser. Since PBJelly and FGAP could not close gaps for the whole human genome, we uniformly applied them to the assembly of the human chromosome 19. The results are expressed in the section “Comparison with other gap-closing tools” and listed in Table 2. The BLAST-based GMcloser and FGAP, and the BLASR-based PBJelly consume much more time than others. Newer tools such as Cobbler and LR_Gapcloser obviously improve the speed and memory consumption relative to prior tools, but they are still slower than TGS-GapCloser. More importantly, the gap-closing accuracy of these tools in terms of scaftig NGA50 (14% of TGS-GapCloser’s on average) and introduced assembly errors (2.4-fold more than TGS-GapCloser on average) are clearly worse than that of TGS-GapCloser, although the number of closed gaps and the improved scaftig continuity are close. Their algorithms need to be adjusted to accept the error-prone input, increase the accuracy in the selection of inserted sequences, and improve the gap-closing efficiency for low coverage of raw long reads.

Comment 5: Installation was very easy but might be helpful to add the tool to conda and linuxbrew but this is a very minor point as there are very few dependencies.

Response: As suggested by the reviewer, we have uploaded the software tool to conda, and are considering to add it to linuxbrew.

1. English AC, Richards S, Han Y, Wang M, Vee V, Qu J, et al. Mind the gap: upgrading genomes with Pacific Biosciences RS long-read sequencing technology. PLoS One. 2012;7 11:e47768. doi:10.1371/journal.pone.0047768.
2. Schneider VA, Graves-Lindsay T, Howe K, Bouk N, Chen HC, Kitts PA, et al. Evaluation of GRCh38 and de novo haploid genome assemblies demonstrates the enduring quality of the reference assembly. Genome Res. 2017;27 5:849-64. doi:10.1101/gr.213611.116.
3. Koren S, Walenz BP, Berlin K, Miller JR, Bergman NH and Phillippy AM. Canu: scalable and accurate long-read assembly via adaptive k-mer weighting and repeat separation. Genome Res. 2017;27 5:722-36. doi:10.1101/gr.215087.116.
4. Walker BJ, Abeel T, Shea T, Priest M, Abouelliel A, Sakthikumar S, et al. Pilon: an integrated tool for comprehensive microbial variant detection and genome assembly improvement. PLoS One. 2014;9 11:e112963. doi:10.1371/journal.pone.0112963.

Additional clarifications:
In addition to the above responses, we also made two changes:
1. We added one citation (Murigneux V., et al. Comparison of long read methods for sequencing and assembly of a plant genome. bioRxiv. 2020:2020.03.16.992933. doi: https://doi.org/10.1101/2020.03.16.992933) as an extra support that TGS-GapCloser leads to an obvious increase in the scaftig N50 with BUSCO detecting more complete genes.
2. In the QUAST evaluations, we changed the human reference assembly from GRCh38.p13 to hs37d5, which excludes ambiguous sequences such as ALT, unplaced and unlocalized sequences to avoid false misassemblies aroused by the alignments between assemblies and those ambiguous segments.
Meanwhile, all spelling and grammatical errors have been checked and corrected.

We look forward to hearing from you in due time regarding our submission and to respond to any further questions and comments you or reviewers may have.

Sincerely,
Mengyang Xu

Close
