# Supplementary material for: TGS-GapCloser: A fast and accurate gap closer for large genomes with low coverage of error-prone long reads
Source: Gigascience. 2020 Sep 7;9(9):giaa094. doi: 10.1093/gigascience/giaa094 (PMC7476103; doi:10.1093/gigascience/giaa094)
Supplement: giaa094_Response_to_Reviewer_Comments_Revision_1 [file giaa094_response_to_reviewer_comments_revision_1.pdf]

Author's Response To Reviewer Comments

Close

Dear Editor,
We are pleased that the manuscript entitled "TGS-GapCloser: A fast and accurate gap closer for large genomes with low coverage of error-prone long reads." (GIGA-D-20-00014R1) is potentially acceptable in GigaScience. Again, we would like to thank you and the reviewers for the time spent on reviewing our manuscript and the comments helping us to improve the article. As suggested, we mainly focused on improving the language in the manuscript. The structure remains unchanged. We then address comments specific to each reviewer below.

Response to Reviewer #1:
Comment: The authors have solved all my previous concerns, and I don't have further comments for the manuscript. I would recommend for an "Accept".

Response: We again appreciate the reviewer's positive comments. It does not require any further revision.

Response to Reviewer #2:
Comment: Most of my technical concerns were addressed well and the authors have done a good job in this regard. At this stage, consider the paper in a state of minor revision but it is on the borderline because I cannot overlook the number of issues I have found in the writing.

Though some of the revisions to the paper have improved the writing, the paper still has some issues that indicate more proof-reading is needed. Again, the paper is written well enough that most readers who have an understanding of assembly algorithms can figure out the intent of what you are trying to say in most cases, but it overall comes off as far too sloppy for publication. To be fair, I have seen many other manuscripts with much worse writing, but there are too many errors to overlook. Based on the type of errors I see, there are hallmarks of correction via grammar checking software, albeit almost a blind acceptance of what the program was spitting out. Grammar checking software is a good tool but not a substitute for proper proofreading, as I imagine is the case for any language. Here are some examples of erroneous or poor writing as well as the possible correction (sequentially from the start of the paper):

- continuity, completeness -> contiguity, completeness (substitutions of contiguity for continuity occur multiple times in the paper, most grammar checking programs would think the use of "contiguity" is an error as it is generally an assembly specific term)
- The development of genome sequencing techniques has been reducing the cost and improving the throughput at a speed beyond the Moore's Law over the last decade -> Genome sequencing techniques have been reducing in cost and improving in throughput at a speed beyond the Moore's Law over the last decade ("the cost/throughput" of what? The use of the preposition "in" ties the subject with these terms)
- progressively increasing focuses move from small bacterial and fungal genomes to large eukaryotes. -> progressively increasing a focus from smaller bacterial and fungal genomes to larger eukaryotes genomes. (this was just wrong, but I do understand the intent)
- BioNano physical map[10], provides -> BioNano physical maps [10], provide
- relative to the NGS-based assembly -> relative to pure NGS-based assemblies.
- the limitation of sequencing platform -> limitations of sequencing platforms
- and the trade-off of algorithms -> and algorithms trade-offs
- The first effort to finish gaps in draft genome assemblies was made -> The first efforts to finish gaps in draft genome assemblies were made
- The NGS technologies -> NGS technologies
- overcame the financial problem -> overcame this financial problem
- of large CPU and memory consuming -> of large CPU and memory consumption

I have only provided only corrections for the first few pages of the paper (up to page 4). Given the number of errors in such a short span of the manuscript, I think you can see why I am concerned. Reviewers are not copy-editors but these errors are quite minor and if they only occurred a few times I would have accepted this paper and have just provided corrections for all of them. Please consider having someone with a good grasp of the English language (ideally with an understanding of assembly) edit the work. Structurally the organization of ideas of the paper is done well; the authors clearly have an understanding of how to communicate science but it is unfortunate English can be such a frustrating language to use, yet is also the de-facto language of science.

Response: We would like to thank the reviewer for raising this writing quality issue. All the errors mentioned by the reviewer have been corrected. To further improve the English, we have invited two bioinformatics experts to carefully go through the manuscript: Dr. Brock Peters is a native English speaker in the US, and Dr. Yongwei Zhang has been working in the US for more than 20 years. Both of them have made extensive polishing, and thus are listed as co-authors. We hope that the current writing quality can meet the requirement of publishing.

We look forward to hearing from you and would like to respond to any further questions and comments you or reviewers may have.

Sincerely,
Mengyang Xu

Close
