# Supplementary material for: TGS-GapCloser: A fast and accurate gap closer for large genomes with low coverage of error-prone long reads
Source: Gigascience. 2020 Sep 7;9(9):giaa094. doi: 10.1093/gigascience/giaa094 (PMC7476103; doi:10.1093/gigascience/giaa094)
Supplement: giaa094_Reviewer_1_Report_Original_Submission — Chong Chu -- 3/19/2020 Reviewed [file giaa094_reviewer_1_report_original_submission.pdf]

Reviewer Report

Title: TGS-GapCloser: A fast and accurate gap closer for large genomes with low coverage of error-prone long reads

Version: Original Submission **Date:** 3/19/2020

Reviewer name: Chong Chu

Reviewer Comments to Author:

The authors present a new method for gap closing with low coverage raw long reads. Experiments on draft genomes with contigs assembled from short reads and scaffolds from long range reads show the tool works well, and more efficiently compared to other tools. The manuscript overall is written and organized well. But I have the following concerns:

1. With more long reads sequenced, more genomes are directly assembled from long reads and then scaffolded or phased together with HiC and/or linked reads. Even though the cost is higher than short reads, different from applications like SV calling, genome assembly is done once and most of the cases the cost could be tolerated. The target application of the proposed method is using low coverage long reads to fill the gaps on draft genome assembled from short reads. The authors need to clearly define how "low" coverage they could perform better than with direct assembly from long reads. The authors show the performance of their tool on different coverage of long reads, but only on the draft genome assembled from short reads. How about the genome directly assembled from long reads? Say at 20X? If it is already good enough, then no need for gap closing at this coverage. This is pretty important as it defines the potential roles the proposed method could play.
2. The major advantages in speed and memory cost over other tools come from using other third party tools that perform good, like minimap2, while the compared tools use slow tools like "blast". It's not from method innovation or better algorithm design, although selecting proper tools is also important.
3. HG001 has HiFi long reads released by GIAB from last year (2019). The authors may consider switching to it.

Level of Interest

Please indicate how interesting you found the manuscript: Choose an item.

Quality of Written English

Please indicate the quality of language in the manuscript: Choose an item.

Declaration of Competing Interests

Please complete a declaration of competing interests, considering the following questions:

- Have you in the past five years received reimbursements, fees, funding, or salary from an organisation that may in any way gain or lose financially from the publication of this manuscript, either now or in the future?
- Do you hold any stocks or shares in an organisation that may in any way gain or lose financially from the publication of this manuscript, either now or in the future?
- Do you hold or are you currently applying for any patents relating to the content of the manuscript?
- Have you received reimbursements, fees, funding, or salary from an organization that holds or has applied for patents relating to the content of the manuscript?
- Do you have any other financial competing interests?
- Do you have any non-financial competing interests in relation to this paper?

If you can answer no to all of the above, write 'I declare that I have no competing interests' below. If your reply is yes to any, please give details below.

I declare that I have no competing interests.

I agree to the open peer review policy of the journal. I understand that my name will be included on my report to the authors and, if the manuscript is accepted for publication, my named report including any attachments I upload will be posted on the website along with the authors' responses. I agree for my report to be made available under an Open Access Creative Commons CC-BY license (http://creativecommons.org/licenses/by/4.0/). I understand that any comments which I do not wish to be included in my named report can be included as confidential comments to the editors, which will not be published.

Choose an item.

To further support our reviewers, we have joined with Publons, where you can gain additional credit to further highlight your hard work (see: https://publons.com/journal/530/gigascience). On publication of this paper, your review will be automatically added to Publons, you can then choose whether or not to claim your Publons credit. I understand this statement.

Yes Choose an item.
