# Supplementary material for: TGS-GapCloser: A fast and accurate gap closer for large genomes with low coverage of error-prone long reads
Source: Gigascience. 2020 Sep 7;9(9):giaa094. doi: 10.1093/gigascience/giaa094 (PMC7476103; doi:10.1093/gigascience/giaa094)
Supplement: giaa094_Reviewer_2_Report_Original_Submission — Justin Chu -- 3/20/2020 Reviewed [file giaa094_reviewer_2_report_original_submission.pdf]

Reviewer Report

Title: TGS-GapCloser: A fast and accurate gap closer for large genomes with low coverage of error-prone long reads

Version: Original Submission **Date:** 3/20/2020

Reviewer name: Justin Chu

Reviewer Comments to Author:

1. The authors present a novel gap-filling algorithm that utilizes low coverage long read data, useful for augmenting existing assemblies. The algorithm appears sound and would be very useful for the bioinformatics community and experimental design and measurements/evaluations, for the most part, seem well designed. However, some more evaluations and comparisons to more tools may be needed. Also, the manuscript has some issues with the quality of the writing.

The writing of the manuscript needs work. Interestingly, it appears that attempts at correction were done but the manuscript seems like it had been proofread and edited by someone who didn't understand the content or just run through grammar checking software without consideration of the content, resulting in a manuscript that was somewhat difficult to read and inaccurate in some places. Giving the authors the benefit of the doubt it is possible to piece together the reasonable intent within the writing, but this takes a great deal of effort and guesswork.

For example, the problems with writing the paper start at the very beginning in the background of the abstract which states that "gap-closing tools suffer multi-alignments and high error rates". The authors likely intend for it to read something like "the long reads suffer from high error rates which reduce the performance of current long read based gap closing tools". Following this statement, they claim that this results in a "huge time and money costs" but these issues are not logically a direct result of the previous point. Perhaps the authors intended to state something like "due to the poor performance of these tools, high long read coverage is needed resulting in huge time and monetary costs". This kind of issue is just the tip of the iceberg of problems related to the overall writing of the paper and the authors need to be more careful in their writing in general to make this manuscript of publishable quality.

2. The mention of the correctness of the assembly (q46) in the manuscript is somewhat meaningless unless compared to the quality before the tool was run. It might also make more sense if the authors state the correctness of only the filled regions, as this quality metric is likely washed out by the overall assembly quality.

3. Contig NG50 was specified in the abstract but gap-filling typically is performed on scaffolds and one would not expect NG50 to increase substantially after gap-filling. Did the authors mean scaftig NG50? Better yet, perhaps scaftig NGA50 should be stated in the abstract instead. I noticed scaftigs seemed to be mentioned in the manuscript equating them to contigs; to prevent confusion consider replacing all instances of contig with scaftig.

4. It seems strange to me that various tools like LR-Gapcloser were not even benchmarked and merely stated that they "did not show any obvious improvements in efficiency and accuracy". Indeed, if PB-Jelly published in 2012 was compared, why were these other tools omitted? Other tools omitted from

comparisons are Cobbler (Warren et al. 2016), GMcloser (Kosugi et al. 2015) and others may exist all of which should be considered in the comparisons unless a compelling reason they did not can be convincingly stated.
If the tools cannot be run due to resource limitations, authors should at least make this clear by benchmarking on smaller datasets and show how resource usage scales or at the very least state the algorithm does not run given their resources (explaining possible reasons why it does not scale). Unless the authors can definitively prove that these tools are not worth benchmarking as they have already shown performance less than the current state-of-the-art tools (to which TGS-GapCloser is compared to) in other publications in almost all dimensions, it doesn't make sense to me that these tools should be omitted from the comparisons.
5. Installation was very easy but might be helpful to add the tool to conda and linuxbrew but this is a very minor point as there are very few dependencies.

Level of Interest

Please indicate how interesting you found the manuscript: Choose an item.

Quality of Written English

Please indicate the quality of language in the manuscript: Choose an item.

Declaration of Competing Interests

Please complete a declaration of competing interests, considering the following questions:

- Have you in the past five years received reimbursements, fees, funding, or salary from an organisation that may in any way gain or lose financially from the publication of this manuscript, either now or in the future?
- Do you hold any stocks or shares in an organisation that may in any way gain or lose financially from the publication of this manuscript, either now or in the future?
- Do you hold or are you currently applying for any patents relating to the content of the manuscript?
- Have you received reimbursements, fees, funding, or salary from an organization that holds or has applied for patents relating to the content of the manuscript?
- Do you have any other financial competing interests?
- Do you have any non-financial competing interests in relation to this paper?

If you can answer no to all of the above, write 'I declare that I have no competing interests' below. If your reply is yes to any, please give details below.

I declare that I have no competing interests

I agree to the open peer review policy of the journal. I understand that my name will be included on my report to the authors and, if the manuscript is accepted for publication, my named report including any attachments I upload will be posted on the website along with the authors' responses. I agree for my report to be made available under an Open Access Creative Commons CC-BY license (http://creativecommons.org/licenses/by/4.0/). I understand that any comments which I do not wish to be included in my named report can be included as confidential comments to the editors, which will not be published.

Choose an item.

To further support our reviewers, we have joined with Publons, where you can gain additional credit to further highlight your hard work (see: https://publons.com/journal/530/gigascience). On publication of this paper, your review will be automatically added to Publons, you can then choose whether or not to claim your Publons credit. I understand this statement.

Yes Choose an item.
