# Supplementary material for: TGS-GapCloser: A fast and accurate gap closer for large genomes with low coverage of error-prone long reads
Source: Gigascience. 2020 Sep 7;9(9):giaa094. doi: 10.1093/gigascience/giaa094 (PMC7476103; doi:10.1093/gigascience/giaa094)
Supplement: giaa094_Reviewer_2_Report_Revision_1 — Justin Chu -- 6/10/2020 Reviewed [file giaa094_reviewer_2_report_revision_1.pdf]

Reviewer Report

Title: TGS-GapCloser: A fast and accurate gap closer for large genomes with low coverage of error-prone long reads

Version: Revision 1 **Date:** 6/10/2020

Reviewer name: Justin Chu

Reviewer Comments to Author:

Most of my technical concerns were addressed well and the authors have done a good job in this regard. At this stage, consider the paper in a state of minor revision but it is on the borderline because I cannot overlook the number of issues I have found in the writing.
Though some of the revisions to the paper have improved the writing, the paper still has some issues that indicate more proof-reading is needed. Again, the paper is written well enough that most readers who have an understanding of assembly algorithms can figure out the intent of what you are trying to say in most cases, but it overall comes off as far too sloppy for publication. To be fair, I have seen many other manuscripts with much worse writing, but there are too many errors to overlook. Based on the type of errors I see, there are hallmarks of correction via grammar checking software, albeit almost a blind acceptance of what the program was spitting out. Grammar checking software is a good tool but not a substitute for proper proofreading, as I imagine is the case for any language. Here are some examples of erroneous or poor writing as well as the possible correction (sequentially from the start of the paper):

- continuity, completeness -> contiguity, completeness (substitutions of contiguity for continuity occur multiple times in the paper, most grammar checking programs would think the use of "contiguity" is an error as it is generally an assembly specific term)
- The development of genome sequencing techniques has been reducing the cost and improving the throughput at a speed beyond the Moore's Law over the last decade -> Genome sequencing techniques have been reducing in cost and improving in throughput at a speed beyond the Moore's Law over the last decade ("the cost/throughput" of what? The use of the preposition "in" ties the subject with these terms)
- progressively increasing focuses move from small bacterial and fungal genomes to large eukaryotes. -> progressively increasing a focus from smaller bacterial and fungal genomes to larger eukaryotes genomes. (this was just wrong, but I do understand the intent)
- BioNano physical map[10], provides -> BioNano physical maps [10], provide
- relative to the NGS-based assembly -> relative to pure NGS-based assemblies.
- the limitation of sequencing platform -> limitations of sequencing platforms
- and the trade-off of algorithms -> and algorithms trade-offs
- The first effort to finish gaps in draft genome assemblies was made -> The first efforts to finish gaps in draft genome assemblies were made
- The NGS technologies -> NGS technologies
- overcame the financial problem -> overcame this financial problem

- of large CPU and memory consuming -> of large CPU and memory consumption
I have only provided only corrections for the first few pages of the paper (up to page 4). Given the number of errors in such a short span of the manuscript, I think you can see why I am concerned. Reviewers are not copy-editors but these errors are quite minor and if they only occurred a few times I would have accepted this paper and have just provided corrections for all of them. Please consider having someone with a good grasp of the English language (ideally with an understanding of assembly) edit the work. Structurally the organization of ideas of the paper is done well; the authors clearly have an understanding of how to communicate science but it is unfortunate English can be such a frustrating language to use, yet is also the de-facto language of science.

Level of Interest

Please indicate how interesting you found the manuscript: Choose an item.

Quality of Written English

Please indicate the quality of language in the manuscript: Choose an item.

Declaration of Competing Interests

Please complete a declaration of competing interests, considering the following questions:

- Have you in the past five years received reimbursements, fees, funding, or salary from an organisation that may in any way gain or lose financially from the publication of this manuscript, either now or in the future?
- Do you hold any stocks or shares in an organisation that may in any way gain or lose financially from the publication of this manuscript, either now or in the future?
- Do you hold or are you currently applying for any patents relating to the content of the manuscript?
- Have you received reimbursements, fees, funding, or salary from an organization that holds or has applied for patents relating to the content of the manuscript?
- Do you have any other financial competing interests?
- Do you have any non-financial competing interests in relation to this paper?

If you can answer no to all of the above, write 'I declare that I have no competing interests' below. If your reply is yes to any, please give details below.

I declare that I have no competing interests

I agree to the open peer review policy of the journal. I understand that my name will be included on my report to the authors and, if the manuscript is accepted for publication, my named report including any

attachments I upload will be posted on the website along with the authors' responses. I agree for my report to be made available under an Open Access Creative Commons CC-BY license (http://creativecommons.org/licenses/by/4.0/). I understand that any comments which I do not wish to be included in my named report can be included as confidential comments to the editors, which will not be published.

Choose an item.

To further support our reviewers, we have joined with Publons, where you can gain additional credit to further highlight your hard work (see: https://publons.com/journal/530/gigascience). On publication of this paper, your review will be automatically added to Publons, you can then choose whether or not to claim your Publons credit. I understand this statement.

Yes Choose an item.
